# A Gene Regulatory Network for Root Epidermis Cell Differentiation in Arabidopsis

**DOI:** 10.1371/journal.pgen.1002446

**Published:** 2012-01-12

**Authors:** Angela Bruex, Raghunandan M. Kainkaryam, Yana Wieckowski, Yeon Hee Kang, Christine Bernhardt, Yang Xia, Xiaohua Zheng, Jean Y. Wang, Myeong Min Lee, Philip Benfey, Peter J. Woolf, John Schiefelbein

**Affiliations:** 1 Department of Molecular, Cellular, and Developmental Biology, University of Michigan, Ann Arbor, Michigan, United States of America; 2 Department of Chemical Engineering, University of Michigan, Ann Arbor, Michigan, United States of America; 3 Department of Biology, Yonsei University, Seoul, Korea; 4 Department of Biology and IGSP Center for Systems Biology, Duke University, Durham, North Carolina, United States of America; Stanford University, United States of America

## Abstract

The root epidermis of Arabidopsis provides an exceptional model for studying the molecular basis of cell fate and differentiation. To obtain a systems-level view of root epidermal cell differentiation, we used a genome-wide transcriptome approach to define and organize a large set of genes into a transcriptional regulatory network. Using cell fate mutants that produce only one of the two epidermal cell types, together with fluorescence-activated cell-sorting to preferentially analyze the root epidermis transcriptome, we identified 1,582 genes differentially expressed in the root-hair or non-hair cell types, including a set of 208 “core” root epidermal genes. The organization of the core genes into a network was accomplished by using 17 distinct root epidermis mutants and 2 hormone treatments to perturb the system and assess the effects on each gene's transcript accumulation. In addition, temporal gene expression information from a developmental time series dataset and predicted gene associations derived from a Bayesian modeling approach were used to aid the positioning of genes within the network. Further, a detailed functional analysis of likely bHLH regulatory genes within the network, including *MYC1*, *bHLH54*, *bHLH66*, and *bHLH82*, showed that three distinct subfamilies of bHLH proteins participate in root epidermis development in a stage-specific manner. The integration of genetic, genomic, and computational analyses provides a new view of the composition, architecture, and logic of the root epidermal transcriptional network, and it demonstrates the utility of a comprehensive systems approach for dissecting a complex regulatory network.

## Author Summary

A current challenge in the field of developmental biology is to define the composition and organization of gene networks that direct the pattern and differentiation of cells, tissues, and organs. In this study, we address this problem using Arabidopsis root epidermis development, a relatively simple model for studies of cell pattern formation and differentiation in plants. We used a tissue-specific cell sorting approach to define more than 1,500 genes whose transcripts differentially accumulate in the developing root epidermis. A series of transcriptome analyses were performed with 17 root epidermal mutants and 2 plant hormone treatments to dissect the regulatory relationships between 208 core genes. In addition, gene expression information from a developmental time series dataset was used to organize genes temporally. The results provide insight into the composition, organization, and logic of a developmental gene regulatory network. Furthermore, this work demonstrates the utility of an integrated analysis in gene regulatory network construction using genetic, genomic, and computational approaches.

## Introduction

A current goal in molecular biology research is to understand the organization and logic of complex gene regulatory networks. To this end, genome-scale approaches have been used to generate large datasets concerning the identity and expression of genes in time and space. Although there is great interest in using expression datasets to understand transcriptional regulation of gene pathways [1], [2], we currently have only a rudimentary understanding of the way genes are organized into and coordinately function in complex networks to generate the flexibility and stability inherent in many biological processes.

**Figure 1:**
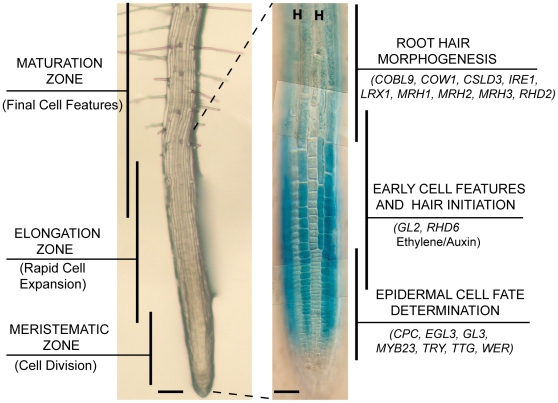
Development of the Arabidopsis root epidermis. Low-magnification images of root tips from Arabidopsis seedling roots showing the series of developmental events that occur from undifferentiated cells (bottom) to mature cells (top). Left: The three major zones of developmental activities are indicated. Scale bar: 100 µm. Right: A root expressing the non-hair cell marker *GL2::GUS* illustrates the file-specific pattern of developing hair cell files (unstained; indicated as “H”) and non-hair cell files (blue-stained cells). Major epidermal differentiation events are indicated, together with a list of genes known to be involved in each event. Scale bar: 50 µm.

The Arabidopsis root epidermis provides a potentially useful model for studying gene networks, due to its developmental simplicity and abundant molecular genetic resources [3], [4], [5]. The root epidermis is composed of a single layer of cells organized into rows (or files) whose entire developmental history has been defined from embryonic origin to mature cell types [6], [7], [8] (Figure 1). Continuous transverse divisions in the root meristematic region generate new epidermal cells that become progressively more differentiated as they age, ultimately becoming root hair cells or non-hair cells. These two cell types arise in a position-dependent pattern, with root-hair cells specified over the intercellular space between underlying cortical cells (the “H” cell position) and non-hair cells developing over a single cortical cell (the “N” position), implying that positional cues play a role in cell fate determination [9], [10] (Figure 1). In addition to differing in the formation of a root hair (a long tubular extension that extends via polarized unicellular (tip) growth), the root hair cells and non-hair cells exhibit differences in their rate of cell division [11], cell length [9], [12], cytoplasmic density [9], [10], vacuolation rate [10], cell surface features [9], [13] and chromatin organization [14], which indicate that these cell types undergo distinct cell differentiation programs.

A large collection of genes influencing root epidermal cell differentiation has been identified using forward and reverse genetic approaches (Figure 1) [5], [15]. Five genes, *TRANSPARENT TESTA GLABRA* (*TTG*), *GLABRA3* (*GL3*), *ENHANCER OF GLABRA3* (*EGL3*), *WEREWOLF* (*WER*), and *MYB23* encode transcription factors that act at an early stage to specify the non-hair fate, because mutations in these (alone or in combination) lead to the formation of root-hair cells in place of non-hair cells (“hairy” mutants) [10], [12], [16], [17], [18]. Three genes, *CAPRICE* (*CPC*), *TRIPTYCHON* (*TRY*), and *ENHANCER OF TRY AND CPC* (*ETC1*), help specify the hair cell fate and mutations in them (alone or in combination) cause non-hair cells to develop in place of hair cells (“hairless” mutants) [19], [20], [21]. Current models suggest that TTG (a small WD-repeat protein [22]), GL3 and EGL3 (bHLH transcription factors [23], [24], [25]), and WER and MYB23 (MYB-type transcription factors [17], [18]) act in a central transcriptional complex in the N cells to promote the non-hair cell fate. This central complex also mediates lateral inhibition by promoting transcription of *CPC*, *TRY*, and *ETC1*, which are small one-repeat MYB proteins able to move to adjacent H cells and inhibit WER/MYB23-GL3/EGL3-TTG complex formation [19], [20], [21], [26], [27], [28], [29], [30], [31], [32], [33]. In addition, the central complex represses expression of the *GL3/EGL3* bHLH genes, and as a result, bHLH proteins are thought to move from the H to the N cells [24]. The appropriate hair/non-hair cell-type pattern is proposed to be initiated by positional cues acting through the SCRAMBLED LRR receptor-like kinase to influence the relative abundance of the transcription complexes in the H and N cell positions [34], [35], [36].

The early-acting root epidermis transcription factors are thought to control the expression of numerous genes encoding transcriptional regulators, signaling molecules, enzymes, and structural proteins responsible for cell-type-specific morphogenetic and biochemical events. One of these genes, *GLABRA2* (*GL2*), encodes a homeodomain-leucine-zipper (HD-Zip) transcription factor protein [37] required for non-hair cell differentiation [12], [38]. The *ROOT HAIR DEFECTIVE6* (*RHD6*) gene is likely to be negatively regulated by GL2, and it encodes a basic helix-loop-helix (bHLH) transcription factor that, together with a related bHLH protein (RSL1), is required for root-hair cell differentiation [39], [40], [41]. Later morphogenesis events in the epidermal cells are influenced by proteins controlling cell wall biosynthesis, cytoskeletal activities, and production/trafficking of extracellular materials, such as COW1, COBL9, IRE1, LRX1, and the MRH proteins (reviewed in [5], [15]) (Figure 1). Root hair cell differentiation is also influenced by the plant hormones auxin and ethylene, which promote hair initiation and morphogenesis, although the precise molecular basis is unclear [40], .

To expand our understanding of the composition, organization, and function of the gene network that governs root epidermis differentiation, we conducted a large-scale comparative transcriptome analysis using root epidermis mutants and plant hormone treatments. We reasoned that perturbing the network with specific mutations and treatments would elicit transcriptional changes that could be used to dissect the network and define the relative positions of the member genes. Further, we combined this approach with temporal gene expression information from a developmental time series dataset as well as the molecular genetic analyses of selected newly identified transcription factor genes. Together, the results provide new insights into the gene regulatory network controlling root epidermis development, including the kinds of component genes, their organization into distinct transcriptional branches, and their response to external factors. These results provide a resource for future studies and demonstrate the utility of a mutant-based transcriptome analysis for dissecting the architecture of a regulatory network.

## Results

### Identification of Genes in the Root Epidermal Cell Differentiation Pathway

**Figure 2:**
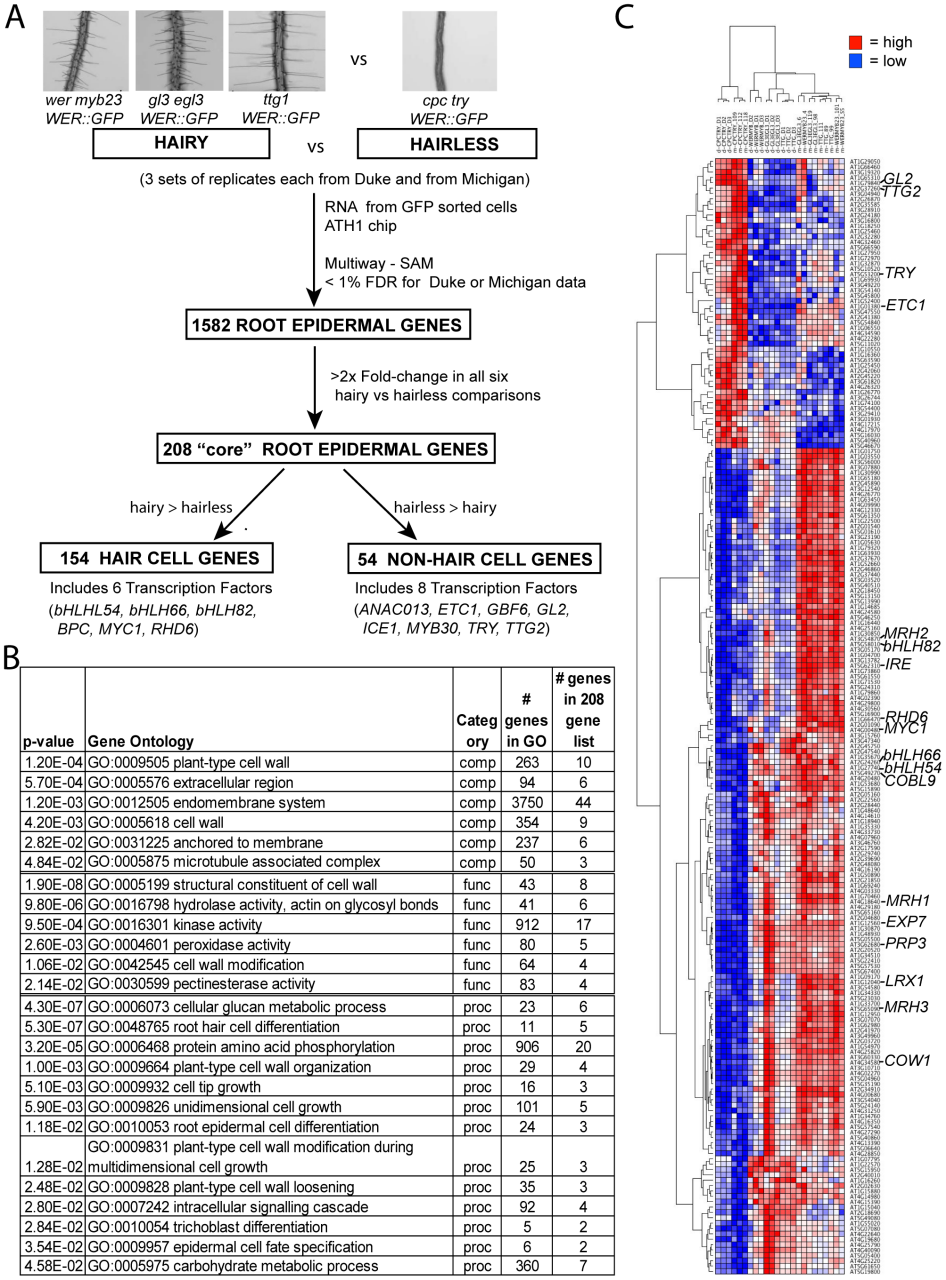
Identification of genes in the root epidermis differentiation pathway. (A) Flow chart of the steps used to define 154 root-hair cell genes and 54 non-hair cell genes used to build the root epidermal gene network. (B) List of gene ontology (GO) categories that are significantly (p<0.05) overrepresented among the core 208 root epidermal genes. (C) Hierarchical clustering of the 208 core root epidermal genes, based on their relative transcript accumulation in Affymetrix ATH1 microarrays using *WER::GFP*-expressing cells from three replicates of the *wer myb23*, *gl3 egl3*, *ttg*, and *cpc try* mutants. Red = high transcript level; Blue = low transcript level. The order of microarray samples along the x-axis is as follows: Columns 1–3, *cpc try* (Duke); Columns 4–6, *cpc try* (Mich); Columns 7–9, *wer myb23* (Duke); Columns 10–12, *gl3 egl3* (Duke); Columns 13–15, *ttg* (Duke), Column 16, *gl3 egl3* (Mich); Column 17, *wer myb23* (Mich); Columns 18–19, *gl3 egl3* (Mich); Columns 20–22, *ttg* (Mich); Columns 23–24, *wer myb23* (Mich). On the right side, specific gene names represent the genes analyzed in the mutant microarrays or genes previously known to be regulated by the WER/MYB23-GL3/EGL3-TTG pathway.

As a first step toward generating a gene regulatory network for root epidermis development, we defined a collection of genes involved in this process. Given that the WER/MYB23, GL3/EGL3, TTG, and CPC/TRY are the earliest-known transcriptional regulators of the root-hair/non-hair cell fates, we conducted a microarray-based comparison of lines homozygous for mutations in these to identify genes under their transcriptional control and preferentially expressed in one or the other cell type (Figure 2A). Three of the mutant lines produce excessive root-hair cells (“hairy lines”; *wer myb23*, *gl3 egl3*, and *ttg*) and one line produces only non-hair cells (‘hairless line”; *cpc try*) (Figure S1C; Table S1). We reasoned that employing multiple independent hairy mutant lines would maximize the robustness of our analysis by eliminating potential false positives caused by effects on processes unrelated to epidermis development.

To focus on gene expression in the developing root epidermis, the *WER::GFP* transgene [17] was incorporated into each of the four mutant backgrounds and *WER::GFP*-expressing cells and their RNA were obtained via a fluorescence-activated cell sorting (FACS) approach [44], [45]. The *WER::GFP* marker was selected for this purpose because (1) *WER* acts at the top of the epidermal specification hierarchy and its spatial expression is not significantly affected by alterations in other components in the network (Figure S2), (2) *WER::GFP* is expressed in all stages of differentiating root epidermal cells, from the initial cells through the final differentiation zone [17], and (3) *WER::GFP* is expressed in both differentiating hair and non-hair cell types, although it is preferentially expressed in the non-hair cells [17] (Figure S2).

Triplicate microarray experiments were conducted, using ATH1 Affymetrix arrays, for each of the four lines (*wer myb23*, *gl3 egl3*, *ttg*, and *cpc try*) at each of two different facilities (at the University of Michigan and at Duke University) to maximize recovery of genotype-dependent transcript differences (six biological replicates for each genotype; 24 total microarrays). A total of 1,582 genes exhibited a significant difference in transcript abundance (q<0.01) between the hairy and hairless mutant lines in at least one of the two comparisons (Michigan or Duke; Figure 2A; Table S2). The proteins encoded by these genes are overrepresented (p<0.05) in gene ontology (GO) classes consistent with root epidermis activities, including the general categories of cell wall biosynthesis, cellular secretion, and expansion, as well as the specific processes of root hair cell differentiation, epidermal cell fate specification, and root hair elongation (Table S3).

To focus on a smaller set of genes strongly influenced by these transcriptional regulators, we subjected the 1,582 genes to a secondary filter, requiring at least 2.0-fold change in transcript level in each individual comparison between the hairless line (*cpc try*) and the three hairy lines (*wer myb23*, *gl3 egl3*, *ttg*) and in the same direction in both the Duke and Michigan data (a total of six comparisons). We identified 208 genes that satisfied these criteria (referred to as “core root epidermal genes”); including 154 genes with transcripts more abundant in each of the three hairy lines (referred to as “hair genes”), and 54 genes with transcripts more abundant in the *cpc try* line (referred to as “non-hair genes”) (Figure 2; Table S4).

Validation of this root epidermal gene set was provided by data from several independent (non-microarray based) sources. First, this 208 gene set includes all six genes that had previously been shown to be transcriptionally regulated by one or more of the WER/MYB23, GL3/EGL3, TTG, or CPC/TRY transcription factors in the root epidermis: *GL2* [12], [46], *ETC1* [47], [48], *RHD6* [41], *PRP3* [49], *TTG2* [50], and *EXP7* [51]. Also, this list included eight additional genes that have been functionally linked to the process of root epidermis development, via mutational or misexpression studies (*COBL9*, *COW1*, *IRE1*, *LRX1*, *MRH1*, *MRH2*, *MRH3*, *MRH6*; Table S4). In addition, GO analyses demonstrated that genes associated with root epidermis-related categories, such as “root hair cell differentiation”, “cell tip growth”, root epidermal cell differentiation”, and “trichoblast differentiation” are significantly overrepresented (p<0.05) in the 208 gene set (Figure 2B). Finally, a high proportion of genes from this gene set (34/208) contains the consensus sequence for a putative “root hair element” (RHE; [52]) within 1 kb of their predicted translation start sites (Table S4).

### Analysis of New Transcription Factor Genes in the Root Epidermis Pathway

**Figure 3:**
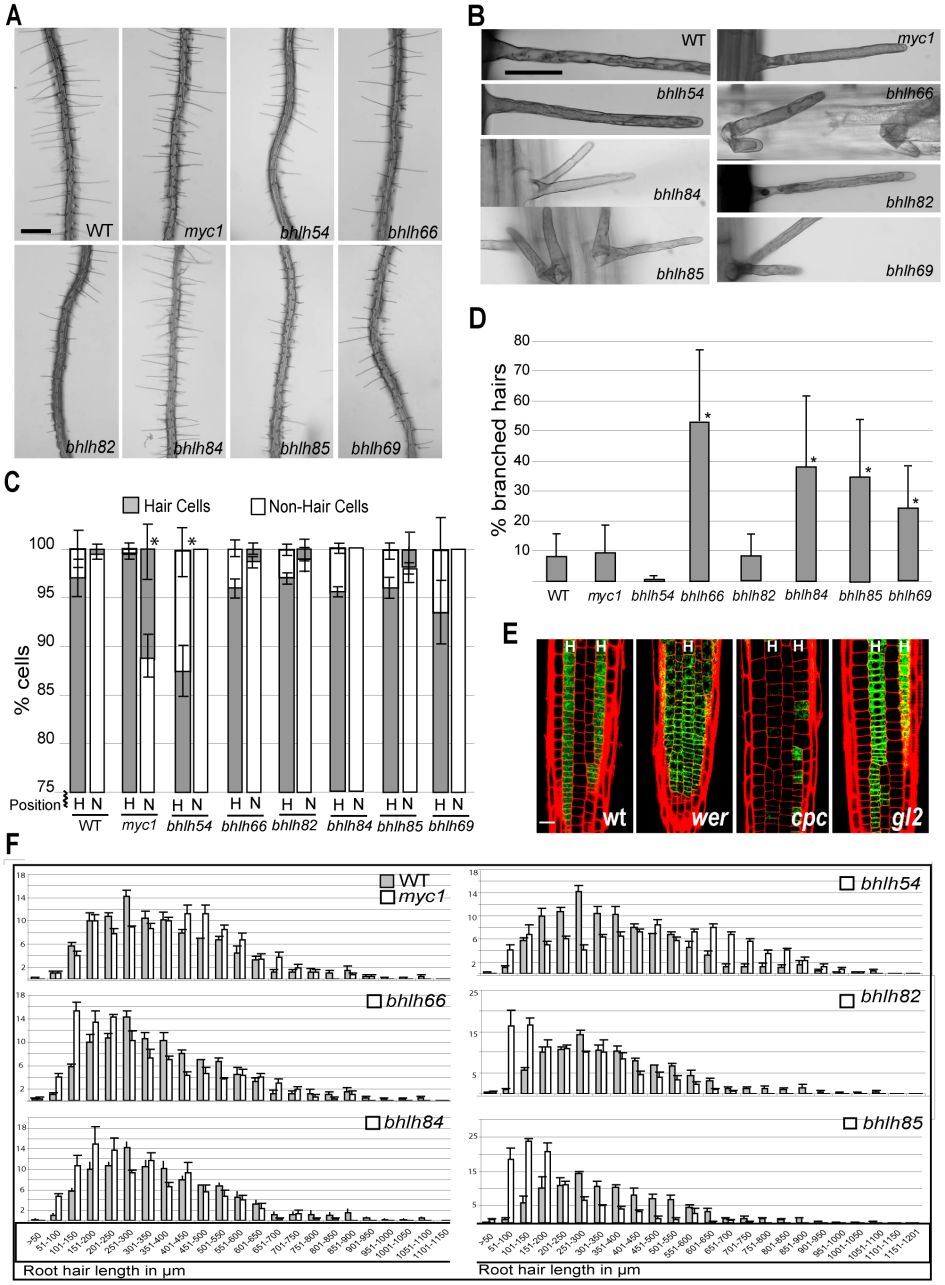
Analysis of bHLH transcription factor genes involved in root epidermis development. (A) Low magnification view of roots from wild-type and homozygous bHLH mutants. Scale bar: 200 µm. (B) High magnification view of individual root hairs from wild-type and each mutant. Scale bar: 30 µm. (C) Cell-type pattern analysis, showing the fraction of root-hair cells and non-hair cells that lie in the H and N cell positions, respectively, of the root epidermis. Mutants which differ significantly from the wild type (p<0.05) are indicated with an asterisk. Some columns lack error bars because all values were identical. (D) Analysis of root hair branching. Mutants which display a significantly greater proportion of branched root hairs than the wild type (p<0.005) are indicated with an asterisk. (E) Expression of the *MYC1::GFP* transcriptional reporter fusion in the root epidermis of wild-type and mutants. The location of H-cell files is designated by “H”. Scale bar: 20 µm. (F) Root hair length in wild-type and bHLH mutants. The length of full-grown root hairs was measured and the number of hairs in each 50 µm class was determined for each mutant line (white bars) and compared to the wild type (gray bars). Each of the six mutants shown here displayed a significantly different distribution of root hair lengths from wild type (p<0.005). The *bHLH69* mutant did not exhibit a significant difference in root hair length distribution and is not shown. In panels (C), (D), and (F), error bars indicate standard deviation.

Using GO analysis and Arabidopsis genome annotation information, 14 of the 208 core root epidermal genes were predicted to encode transcription factors (Table S4; Figure 2A). At the time of this analysis, only five of these 14 were known to be associated with root epidermal development (*TRY*, *ETC1*, *GL2*, *TTG2*, and *RHD6*; Table S4). Among the nine others, four encode bHLH transcription factors preferentially expressed in root hair cells: *MYC1*, *bHLH54*, *bHLH66*, and *bHLH82* (Table 1; Figure 2C; Table S4). Given the importance of other bHLH proteins in the transcriptional control of root epidermis development (e.g. *GL3*, *EGL3*, and *RHD6*), we selected these four genes for detailed study, and multiple homozygous insertion mutants were identified and analyzed for each gene (Table S1; Figure 3).

**Table 1:** Three subfamilies of bHLH genes in Arabidopsis involved in root epidermis development.

bHLH Sub-family*	AGI Gene ID	bHLH Gene Name	Relative Transcript Accumulation in Root Epidermis Cell Types** (ATH1 signal from hairy / non-hairy lines)	
			Michigan Dataset	Duke Dataset
IIIf	*AT1G63650*	*EGL3*	3.8 (0.1)	7.4 (26)
IIIf	*AT4G00480*	*MYC1*	30 (<0.1)	12.5 (2)
IIIf	*AT4G09820*	*TT8*	1.1 (65)	1.0 (79)
IIIf	*AT5G41315*	*GL3*	Not represented on ATH1 chip	
VIIIc	*AT1G27740*	*bHLH54*	37 (<0.1)	11.7 (<0.1)
VIIIc	*AT1G66470*	*RHD6*	37 (<0.1)	10.3 (1.2)
VIIIc	*AT2G14760*	*bHLH84*	Not represented on ATH1 chip	
VIIIc	*AT4G33880*	*bHLH85*	Not represented on ATH1 chip	
VIIIc	*AT5G37800*	*bHLH86*	Not represented on ATH1 chip	
XI	*AT1G03040*	*bHLH7*	0.8 (22)	0.7 (4.8)
XI	*AT2G24260*	*bHLH66*	15.7 (<0.1)	7.7 (<0.1)
XI	*AT4G02590*	*bHLH59*	0.5 (2.2)	0.8 (3.8)
XI	*AT4G30980*	*bHLH69*	Not represented on ATH1 chip	
XI	*AT5G58010*	*bHLH82*	49 (<0.1)	10 (1.5)

*The bHLH gene numbering and subfamily organization (based on structural similarities and bHLH domain sequence) have been previously defined [53].

**Values represent the average fold-change from a multi-way SAM of *wer myb23*, *ttg*, and *gl3 egl3* versus *cpc try*. False discovery rate (q-value) is shown in parentheses.

#### MYC1

The *myc1-1* mutant exhibited a greater proportion of ectopic root-hair cells than wild-type, with approximately 12% of the cells in the non-hair position differentiating as root hair cells (Figure 3A, 3C). Two independent *myc1* mutant lines (SALK_056899c and SALK_006354c) exhibited similar phenotypes (data not shown). This suggests that MYC1 helps specify the non-hair fate, which was unexpected, given that the microarray expression data indicate preferential *MYC1* transcript accumulation in the developing hair cells (Figure 2; Table 1). To examine this further, we generated a *MYC1::GFP* transcriptional reporter fusion and discovered that *MYC1::GFP* plants exhibit preferential GFP accumulation in the differentiating root hair cells (Figure 3E). Further, *MYC1::GFP* expression is present in all epidermal cells in the *wer* mutant, is nearly absent in the *cpc* mutant, and is unaltered in the *gl2* mutant (Figure 3E). These results confirm the microarray results and indicate that *MYC1* is preferentially transcribed in the developing hair cells, is negatively regulated by WER, and is positively regulated by CPC. Because these features of *MYC1* are similar to *GL3* and *EGL3* [23], [24], we analyzed possible genetic interactions between these bHLH genes. We discovered that *myc1-1* is able to enhance the effect of the *gl3-1* and the *egl3-1* mutations on ectopic root hair formation (Figure S3), suggesting that the MYC1 bHLH protein acts redundantly with GL3 and EGL3 in root epidermal patterning.

#### bHLH54

In contrast to *myc1*, the major effect of the *bhlh54-1* mutant is a reduction in the frequency of root-hair cells (and a corresponding increase in non-hair cells), relative to the wild type (Figure 3A, 3C). This suggests that *bHLH54* is required for specification of the hair cell fate and/or for root hair initiation.

#### bHLH66

The *bhlh66-1* mutant exhibits a normal pattern of root epidermal cell types, but its root hairs possess an abnormal morphology (Figure 3). In particular, a high frequency (approximately 50%) of the hairs form branches (Figure 3B, 3D), implying a role for *bHLH66* in regulating root hair elongation.

#### bHLH82

The *bhlh82-1* mutant possessed significantly shorter hairs than the wild type (Figure 3A, 3F), indicating that *bHLH82* is necessary for sustaining root hair elongation. However, no defect in hair branching or cell type pattern formation was observed (Figure 3).

#### bHLH84, bHLH85, and bHLH69

The *MYC1*, *bHLH54*, *bHLH66*, and *bHLH82* genes are members of three subfamilies of bHLHs in Arabidopsis, designated IIIf, VIIIc, and XI [53] (Table 1). We considered the possibility that other members of these bHLH gene subfamilies that are not represented on the ATH1 microarray chip might also be involved in root epidermis development. Indeed, one bHLH gene not represented on the ATH1 chip, *AT5G37800* (also known as *RSL1* and *bHLH86*; [53]) a member of the bHLH VIIIc subfamily (Table 1), had previously been shown to be involved in root hair formation by acting partially redundantly with *RHD6* [41]. To assess other bHLHs in these subfamilies not represented on the ATH1 chip, we analyzed T-DNA insertion lines for the two remaining genes from bHLH subfamily VIIIc (*AT2G14760* = *bHLH84* and *AT4G33880* = *bHLH85*) and the one gene in bHLH subfamily XI not represented on the ATH1 chip (*AT4G30980* = *bHLH69*). We discovered that each of these three bHLH mutant lines has defects in root hair formation (Figure 3). Specifically, each exhibits a significant increase in the percentage of branched root hairs (Figure 3D), and the *bhlh84-1* and *bhlh85-1* mutants showed a significant reduction in root hair length. This suggests that these three bHLH genes are also necessary for appropriate root hair elongation.

Taken together, the presence of mutant phenotypes for each of these bHLH genes indicates broad participation of members of the bHLH subfamilies IIIf, VIIIc, and XI in regulating transcriptional events during many stages of root epidermis development.

### Transcriptome Analysis of Mutants Affecting Root Epidermis Development

To further analyze the 208 root epidermal genes and their organization in a transcriptional regulatory network, we assessed their transcript profiles in lines containing knockout mutations for genes acting at later stages of root epidermal development. A total of 13 diverse root epidermal mutants were selected for this purpose, and the *WER::GFP* marker was incorporated into each mutant background to assess the root epidermis transcriptome.

#### Transcription Factor Gene Knockouts

We first analyzed the effect of mutations in transcription factor genes that are essential for root epidermis development, and thereby most likely to affect transcript accumulation of genes in this network.

**Figure 4:**
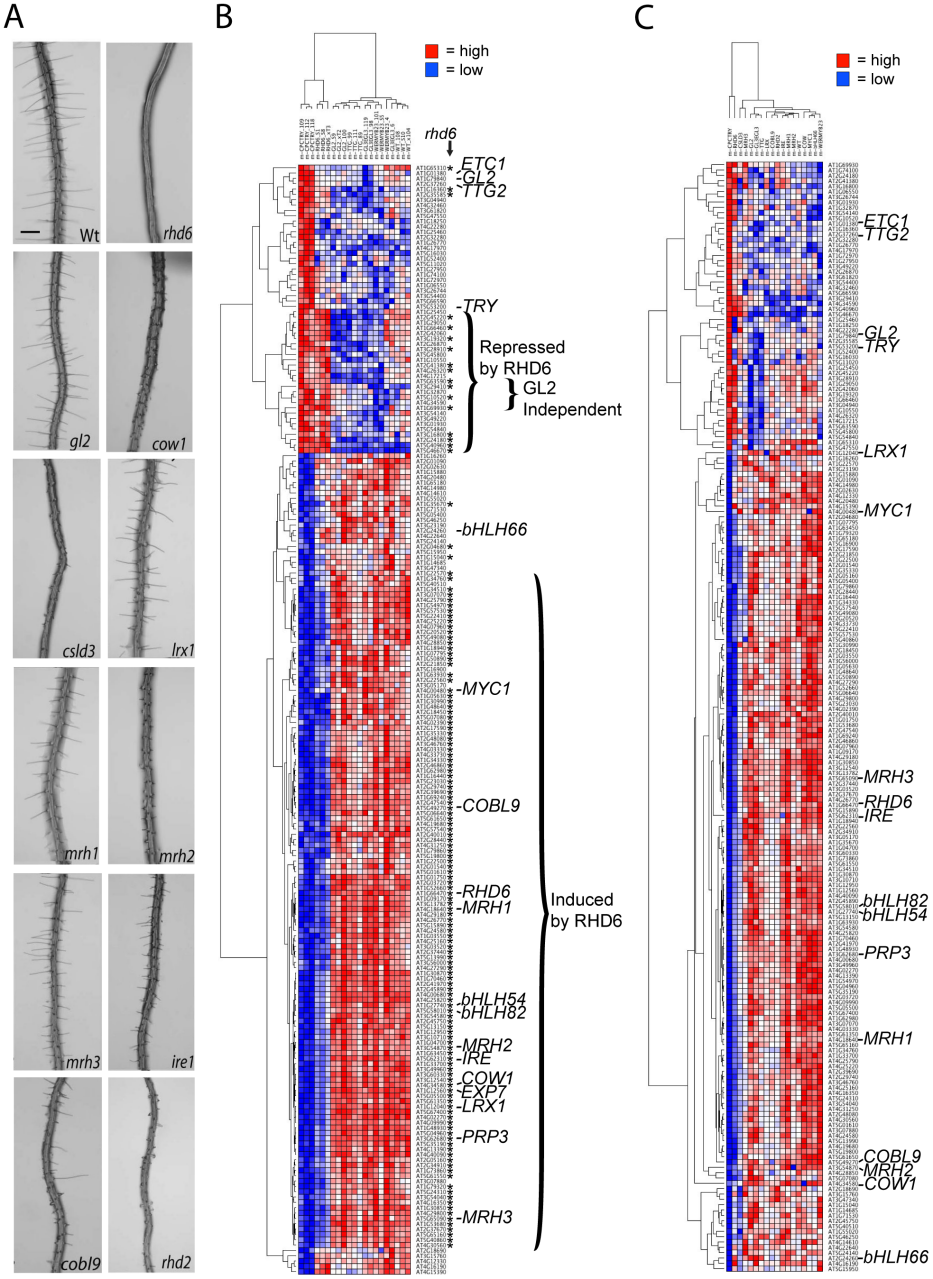
Effect of mutations on expression of the 208 core root epidermal genes. (A) Low magnification view of roots from wild-type and homozygous root epidermis mutants. Scale bar: 250 µm. (B) Hierarchical clustering of the 208 core root epidermal genes, based on their relative transcript accumulation in Affymetrix ATH1 microarrays using *WER::GFP*-expressing cells from three replicates of (left to right) the *cpc try*, *rhd6*, *gl2*, *ttg*, *gl3 egl3*, *wer myb23* mutants and the wild type Columbia. Red = high transcript level; Blue = low transcript level. Asterisks indicate genes significantly affected in the *rhd6* mutant background. (C) Hierarchical clustering of the 208 core root epidermal genes, based on their relative transcript accumulation (averaged from three replicates) in Affymetrix ATH1 microarrays using *WER::GFP*-expressing cells from (left to right) *cpc try*, *rhd6*, *csld3*, *mrh3*, *gl2*, *gl3 egl3*, *ttg*, *lrx1*, *cobl9*, *rhd2*, *ire*, *mrh1*, *mrh2*, *wild type*, *cow1*, *myc1*, *bhlh66*, and *wer myb23*. Red = high transcript level; Blue = low transcript level. Heirarchical clustering of the complete set of three replicates for each line is presented as Figure S5.

The *RHD6* bHLH gene is known to be required for root hair initiation, to be preferentially expressed in developing hair cells, and to be positively regulated by CPC and negatively regulated by TTG, WER, and GL2 [39], [41](Figure 4; Figure S1). We compared transcript levels for the 208 root epidermal genes in *rhd6* versus wild type, by analyzing RNA from sorted cells marked with *WER::GFP*. As expected, most (126) of the 154 root-hair gene transcripts are negatively affected in the developing root epidermis of the *rhd6* mutant (FC>2.0; Figure 4B), including *LRX1* and *PRP3* which had previously been shown to be RHD6 dependent [49], [54]. Among the 28 root-hair gene transcripts that are not substantially altered in the *rhd6* root epidermis, a significance analysis of microarrays (SAM) comparison between the *rhd6* and *cpc try* datasets showed that 24 of these display a significant difference in transcript abundance (FC>2.0; q<0.02), indicating that RHD6 does not regulate transcription of all of the hair cell differentiation genes. Unexpectedly, the *rhd6* mutation also altered transcript accumulation for 17 of the non-hair genes (Figure 4B). Sixteen of these 17 genes exhibit increased transcript levels in *rhd6*, relative to wild-type (FC>2.0), yet 14 of the 16 do not significantly differ between *rhd6* and *cpc try* (FC<2.0, q>0.1). This indicates that the role of RHD6 in promoting root hair formation is not limited to inducing root-hair cell differentiation genes, but it also acts (directly or indirectly) to inhibit transcription of non-hair genes.

The *GL2* homeobox gene is also likely to represent a key node in the root epidermis network, because it is essential for non-hair cell differentiation [12] and it is positively regulated by the WER/MYB23-GL3/EGL3-TTG regulatory complex via direct binding by WER to its promoter [30]. Although non-hair cells are replaced by root-hair cells in the *gl2* mutants (Figure S1, Figure 4A) [12], these ectopic hair cells still display some characteristics of non-hair cells, indicating aspects of non-hair cell differentiation are GL2-independent [10], [12], [23]. Our transcriptome analysis showed that *gl2* alters most of the 208 core epidermal genes in a similar manner as the *wer myb23*, *gl3 egl3*, and *ttg* mutants (204/208 genes exhibit FC>2 in *gl2* versus *cpc try*; Figure 4). This is consistent with the view that *GL2* plays a major role in non-hair cell transcriptional regulation. As an independent test of the effect of GL2 on one of these genes, we used RT-PCR to analyze the *MYB30* RNA level in wild-type and *gl2* mutant roots. In support of the microarray results, a reduction in *MYB30* RNA was observed in the *gl2* background (Figure S4). In contrast to the large fraction of genes exhibiting a similar response to *gl2*, *wer myb23*, *gl3 egl3*, and *ttg*, one cluster of six non-hair genes exhibits higher transcript levels in *gl2*, relative to *wer myb23*, *gl3 egl3* and *ttg* (labeled “GL2 independent” in Figure 4B), and this is supported by a three-way SAM (for each gene, FC>1.3 for *gl2* versus *wer myb23*, *gl3 egl3*, and *ttg*). Thus, these six genes likely represent a group of GL2-independent non-hair cell genes that are positively regulated by WER/MYB23, GL3/EGL3, and TTG, but not by GL2.

In addition to *rhd6* and *gl2*, we selected two of the new bHLH transcription factor gene mutants (*myc1* and *bhlh66*) for *WER::GFP*-based transcriptome analysis. In the root epidermal tissue of each of these mutants, we identified a relatively small number of significant transcript alterations among the 208 core epidermal genes, including 16 genes affected in *myc1* and 10 genes affected in *bhlh66* (FC>2.0; q<0.05) (Figure 4C; Figure S5; Table 2; Table S5; Table S6), which implies that each of these genes affects only a small portion of the root epidermal network and/or that they act in a partially redundant manner with other regulators. Many of these significantly affected genes are non-hair genes (8/16 for *myc1* and 8/10 for *bhlh66*), which is consistent with the phenotypic effect of *myc1* on non-hair cell fate (Figure 3) and indicates that, despite its expression in root-hair cells, bHLH66 has a role in regulating non-hair genes.

**Table 2:** Core root epidermis genes significantly affected in mutant lines.*

Mutant	Root Hair Genes Repressed	Root Hair Genes Induced	Non-Hair Genes Repressed	Non-Hair Genes Induced
*bhlh66*	2	0	4	4
*cobl9*	13	2	6	3
*cow1*	2	6	0	5
*csld3*	70	0	7	9
*ire1*	8	0	3	4
*lrx1*	13	0	3	2
*mrh1*	1	0	1	1
*mrh2*	1	0	0	1
*mrh3*	28	1	2	9
*myc1*	2	6	4	4
*rhd2*	17	1	1	4

*Significant genes display FC>2 and q<0.05. A list of the gene IDs is given in Table S5.

#### Non-Transcription-Factor Gene Knockouts within the Root Epidermal Network

To further probe the gene regulatory pathway, we conducted *WER::GFP*-based FACS and transcriptome analyses with seven mutants defective in non-transcription-factor genes in the 208-gene network. We reasoned that this group of genes (including *COBL9*, *COW1*, *IRE1*, *LRX1*, *MRH1*, *MRH2*, and *MRH3*) may directly or indirectly affect gene transcription in the root hair cells because they encode potential regulatory proteins, such as kinases, phosphatases, or membrane proteins (Table S1; Table S4), they are expressed preferentially in root hair cells (Figure 2), and they cause abnormal root hair growth when mutated (Figure 4A; Figure S1). Our results showed that most of these mutants affect a small number of root epidermal gene transcripts (Figure 4C; Figure S5; Table 2; Table S5) suggesting a minor role for these in transcriptional regulation. The exception was the *mrh3* mutant, which significantly affected 40 root epidermal genes (including a decrease in 28 root-hair expressed gene transcripts), implying that the *MRH3*-encoded phosphatase is more closely linked to transcriptional regulation. Using GO analysis, we found that the genes affected in these seven mutants tend to be associated with cell wall biosynthesis (Table S6), which is consistent with their hair morphogenesis defects.

#### Non-Transcription Factor Gene Knockouts Outside the Network

Mutations in some genes not included among the 208 core genes are known to cause defects in root epidermis development. We used mutants affecting two well-characterized genes of this type, *RHD2* and *CSLD3*, to conduct *WER::GFP* FACS-based transcript profiling. *RHD2* encodes a protein with NADPH oxidase activity that influences accumulation of reactive oxygen species necessary for normal root hair tip growth [55], [56] (Figure S1 and Figure 4A). We found that, relative to the wild type, the *rhd2* mutant significantly alters the root epidermal transcript level of 23 of the 208 core genes (Figure 4C; Figure S5; Table S5) and these are overrepresented in the GO classes related to cell wall biosynthesis (Table S6). The *CSLD3* gene encodes a cellulose synthase-related protein and *csld3* mutants exhibit a reduction in epidermal cell growth and hair bursting (Figure S1 and Figure 4A), likely due to weakened cell wall structure [57], [58]. We observed a large number of gene transcripts affected in the *csld3* mutant (86/208; Figure 4C; Figure S5; Table S5), perhaps due to a general indirect effect of cell bursting on root hair RNA levels. Consistent with this, a majority of the affected genes are root hair genes and affect GO categories related to cell wall and cell expansion (Table S6).

Comparing our overall results from the nine non-transcription factor (downstream) mutant microarrays, we noticed an exceptional group of six root-hair genes that are affected in a majority (≥6) of these mutants. In particular, the transcript level for each of these six genes (AT1G34510, AT2G20520, AT4G28850, AT5G22410, AT5G57530, and AT5G57540) was significantly reduced in the *cobl9*, *ire1*, *lrx1*, *mrh3*, *rhd2*, and *csld3* mutants. It may be that transcription of this group of genes, which encode three cell-wall modifying xyloglucan endotransglucosylase/hydrolases, a fascilin-like arabinogalactan cell wall protein, and two peroxidases involved in oxidative stress response, is particularly sensitive to root hair growth disruption.

### Transcriptome Analysis of Plant Hormones Affecting Root Epidermis Development

**Figure 5:**
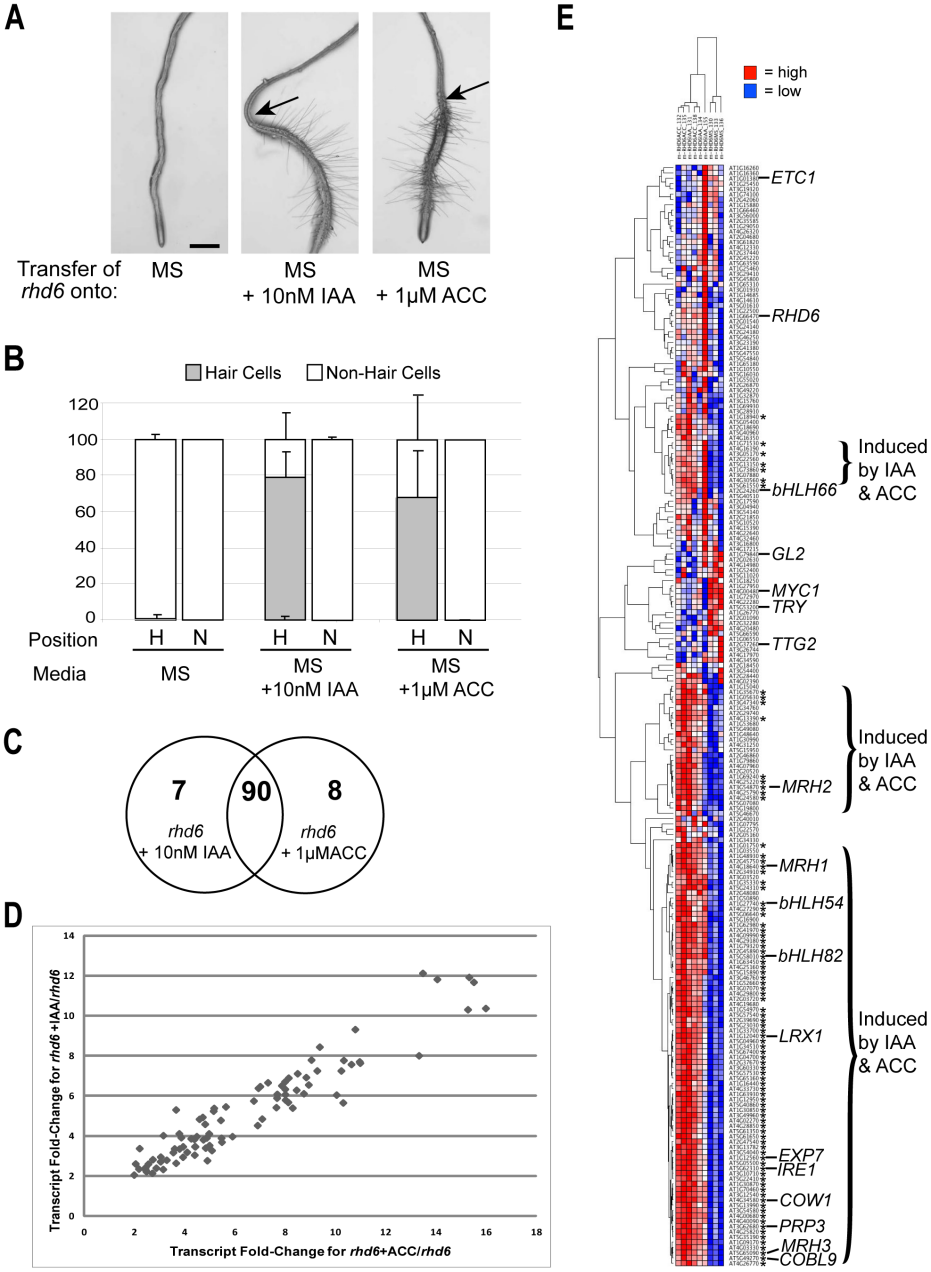
Molecular genetic analysis of root-hair differentiation induced by auxin and ethylene. (A) Roots of *rhd6* seedlings grown for three days on unsupplemented (MS) media, and then transferred to either MS, MS+10 nM IAA, or MS+1 µM ACC and grown for two additional days. Arrows indicate the position of root tip at time of transfer. Scale bar: 200 µm (B) Quantitative analysis of root epidermal cell specification in *rhd6* seedlings grown for three days on unsupplemented (MS) media, and then transferred to either MS, MS+10 nM IAA, or MS+1 µM ACC and grown for two additional days. The root-hair and non-hair cell types were determined from the portion of the root produced in the last two days. (C) Core root epidermal genes significantly affected (>2-fold change; <0.5% FDR) by transfer of *rhd6 WER::GFP* seedlings to either MS+10 nM IAA, or MS+1 µM ACC (relative to transfer to MS). After two days of seedling growth on the transferred media, root epidermal cells were collected by GFP-based cell sorting and the RNA used for ATH1 microarray analysis. (D) Plot of the fold-change for the 90 root epidermal genes induced by IAA and by ACC following transfer of *rhd6 WER::GFP* seedlings to either MS+10 nM IAA, or MS+1 µM ACC. (E) Hierarchical clustering of 208 core root epidermal genes based on their transcript levels on ATH1 chips (triplicate biological replicates) using RNA from developing root epidermal cells in *rhd6 WER::GFP* seedlings grown for three days on unsupplemented (MS) media, and then transferred to either MS, MS+10 nM IAA, or MS+1 µM ACC and grown for two additional days. Red = high transcript level; Blue = low transcript level. Asterisks indicate genes significantly affected by the hormone treatments.

The differentiation of root hair cells is promoted by the plant hormones auxin and ethylene, although the molecular mechanism is unclear [4], [42], [51], [59], [60]. The most robust effect of these hormones is observed in the *rhd6* mutant background, where the block in root hair formation caused by *rhd6* is overcome by addition of 1-amino-cyclopropane-1-carboxylic acid (ACC; an ethylene precursor) or indole-3-acetic acid (IAA; an auxin) to the growth medium, implying that these hormones activate the hair differentiation network at some point downstream or independent of RHD6 [39], [40] (Figure 5A, 5B). Here we exploited this robust effect of auxin and ethylene in the *rhd6* background to further probe the organization of the root epidermal regulatory network.

We discovered that treatment of *rhd6 WER::GFP* seedlings with 10 µM IAA or 1 µM ACC elicited similar transcriptional effects on the 208 genes during root epidermis development, with more than 90% of the genes affected by one treatment also affected by the other (FC>2.0) (Figure 5C) and the relative effect on each gene was similar in each treatment (Figure 5D). This suggests that these two hormone treatments act in a similar molecular manner to induce root hair formation in the *rhd6* mutant, and accordingly, we considered the results of these two treatments together for this study. Among the 90 genes affected by both IAA and ACC treatments, all are root hair genes and all of them exhibited an increase in transcript abundance (Figure 5E), which strongly indicates that these hormones exert their effect on root hair formation (at least in the *rhd6* background) through promoting transcription of genes normally expressed in root hair cells.

### Visualizing and Modeling the Transcriptome Data

To obtain a large-scale view of the relationships among the mutations/treatments across the 208 gene set, we applied multidimensional scaling (MDS) to reduce the high-dimensional gene expression patterns to a two-dimensional representation (Figure S6). This visualization highlights two qualitatively different groups of gene expression patterns. One pattern includes the *rhd6*, *rhd6+MS*, and *cpc try* mutants, which are all phenotypically hairless lines and located farthest from the wild type toward the right in Figure S6. The second pattern, less well isolated, includes the *gl2*, *gl3 egl3*, *ttg*, and *wer myb23* mutants, which all produce excess root-hair cells, and these cluster in the upper left corner in Figure S6. This plot helps validate the transcriptome data as a large-scale reflection of the root epidermis phenotypes in these lines.

Next, we used a computational modeling strategy to obtain another view of the transcriptional relationships between the 208 core genes from the microarray data. A Bayesian network approach was applied, which exploits conditional independence relationships in gene expression levels measured under various conditions to generate a directed acyclic graph of nodes that best predict downstream nodes [61], [62]. An advantage of our dataset for this approach was the use of the 17 knock-out mutants, because they served to fix the direction of parent-to-offspring nodes in the Bayesian-derived relationships. Further, we added two phenotypic nodes to this analysis, representing the characters of “root hair length” and “root hair branching”, so that the gene(s) that best predict each of these characters could be identified. To enable this additional analysis, we conducted a detailed phenotypic characterization of root hair length and degree of root hair branching in each of the wild type and mutant lines (Figure S1, Figure 3D and 3F, and Figure S7; Table S7).

**Figure 6:**
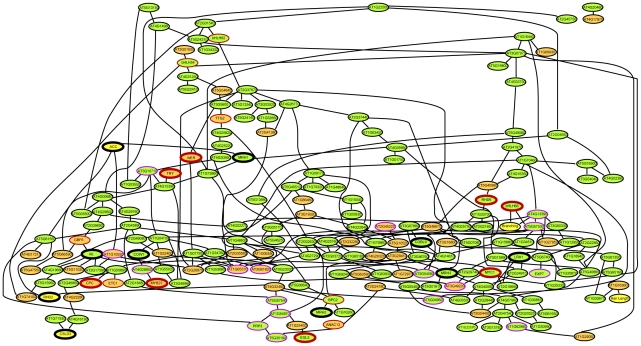
Bayesian modeling from root epidermal transcriptome data. Consensus Bayesian network showing the connections (edges) between 219 possible nodes (208 core root epidermal genes, 7 genes used in microarray mutants, 2 hormone treatments, and 2 root hair phenotypes) that appear in at least 40% of the 42,000 high-scoring networks from among more than 10^9^ total networks analyzed using microarray transcript data from 66 datasets. The directionality of the edges is indicated by arrowheads and by the hierarchy (the higher-positioned node predicts the lower node). Nodes represent core root-hair genes (green fill), core non-hair genes (orange fill), other genes or factors not in the 208 gene list (yellow fill), gene knockouts used for transcriptome analysis (thick outline), genes encoding predicted transcription factors (red-colored outline), and genes encoding predicted cell wall proteins (purple-colored outline). Note that this consensus model illustrates less than 219 nodes because some nodes did not appear in any frequently occurring edges.

Given the large number of nodes in our network (>200), the analysis of all possible Bayesian networks was not computationally practical. As an alternative, we scored a limited number of candidate networks (approximately 10^9^), identified specific edges common among the highest scoring 42,000 networks, and used these to generate a composite Bayesian network that links the individual high-scoring edges (Figure 6). The resulting network predicts transcriptional relationships between particular gene pairs, including ones with known regulatory interactions and groups of similar genes, such as cell wall genes (outlined in purple in Figure 6). In particular, this analysis was useful in defining the relationship between nodes of different types. Specifically, the resulting model identified *AT1G16360* expression as the best predictor of root hair length (via an inverse correlation). Because *AT1G16360* encodes a LEM3/CDC50-like protein related to regulators of polarized secretory activity in yeast [63], this gene may normally act to inhibit root hair tip growth. With respect to the root hair branching character, the composite Bayesian network identified two root-hair genes, *bHLH66* and *AT4G13390* (encoding a proline-rich extensin-like wall protein), as the best predictors of the degree of root hair branching (in an inverse correlation). This suggests a role for these genes in the maintenance of unidirectional tip growth during root hair formation, and it shows how a modeling approach of this type can generate insights not apparent from simple transcript profile comparisons.

### Relative Developmental Timing of Root Epidermal Gene Expression

**Figure 7:**
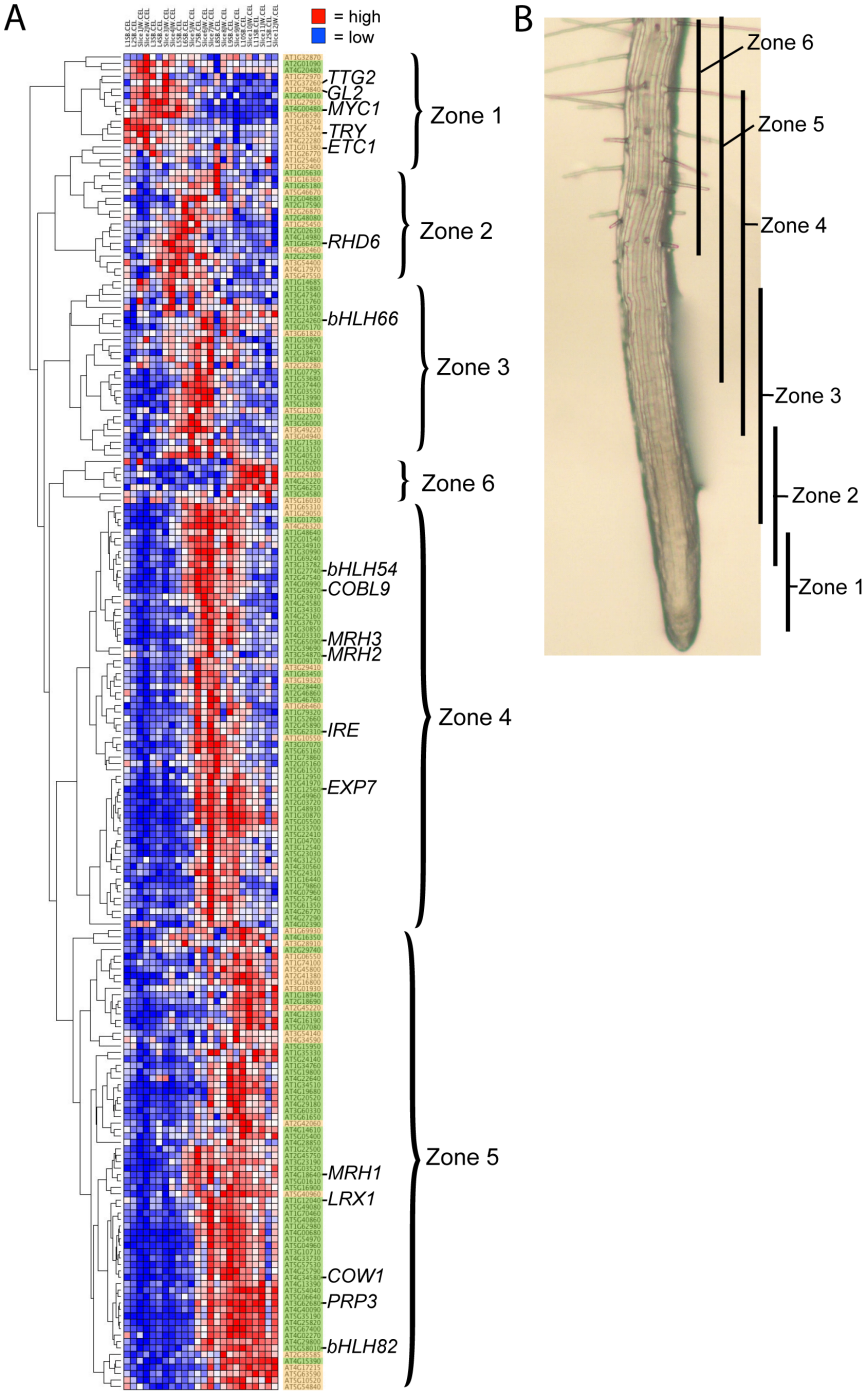
Developmental time-course of transcript accumulation for the 208 core root epidermal genes. (A) Heirarchical clustering of the 208 core root epidermal genes according to transcript accumulation in transverse sections along the longitudinal axis of wild-type Arabidopsis root tips. Clusters of genes with similar developmental expression profiles define six major developmental zones. The root section data were obtained from two independent roots [64], and the 12 sections from each root (numbered 1–12) are organized according to their developmental position in this figure from left to right (along the x-axis). The 208 genes are highlighted in green (for root-hair genes) or yellow (for non-hair genes). (B) The approximate location of cells along the root axis of the zones showing maximal transcript accumulation for the six major clusters of root epidermal genes shown in (A). The position of the bars along the root axis was estimated from the data in panel (A) and reference [64].

To assist the organization of the genes in the epidermal network, we analyzed the relative developmental timing of transcript accumulation for each of the 208 core root epidermal genes. In a previous study, gene transcript levels along the length of the Arabidopsis root tip were profiled by microdissection of two independent roots into 12 sections (approximately 3–5 cells per section) [64]. Using these temporal transcriptome datasets, we defined the developmental profiles for the 208 core genes and used hierarchical clustering to place each within a group, based on the timing of their maximal transcript accumulation (Figure 7). This analysis revealed six major zones of temporal gene activity (named Zone 1–6) for root epidermis development, and it was validated by comparing previously determined non-microarray-based expression profiles for known genes (e.g. *GL2*, *RHD6*, *PRP3*). Interestingly, we noticed that a relatively large number (22) of the genes in Zones 1 or 2 (35) are non-hair genes, whereas the root hair genes tend to be located in Zones 4 and 5 (Figure 7), which may reflect the importance of defining the non-hair cell fate at an early stage.

### Construction of a Gene Regulatory Network

**Figure 8:**
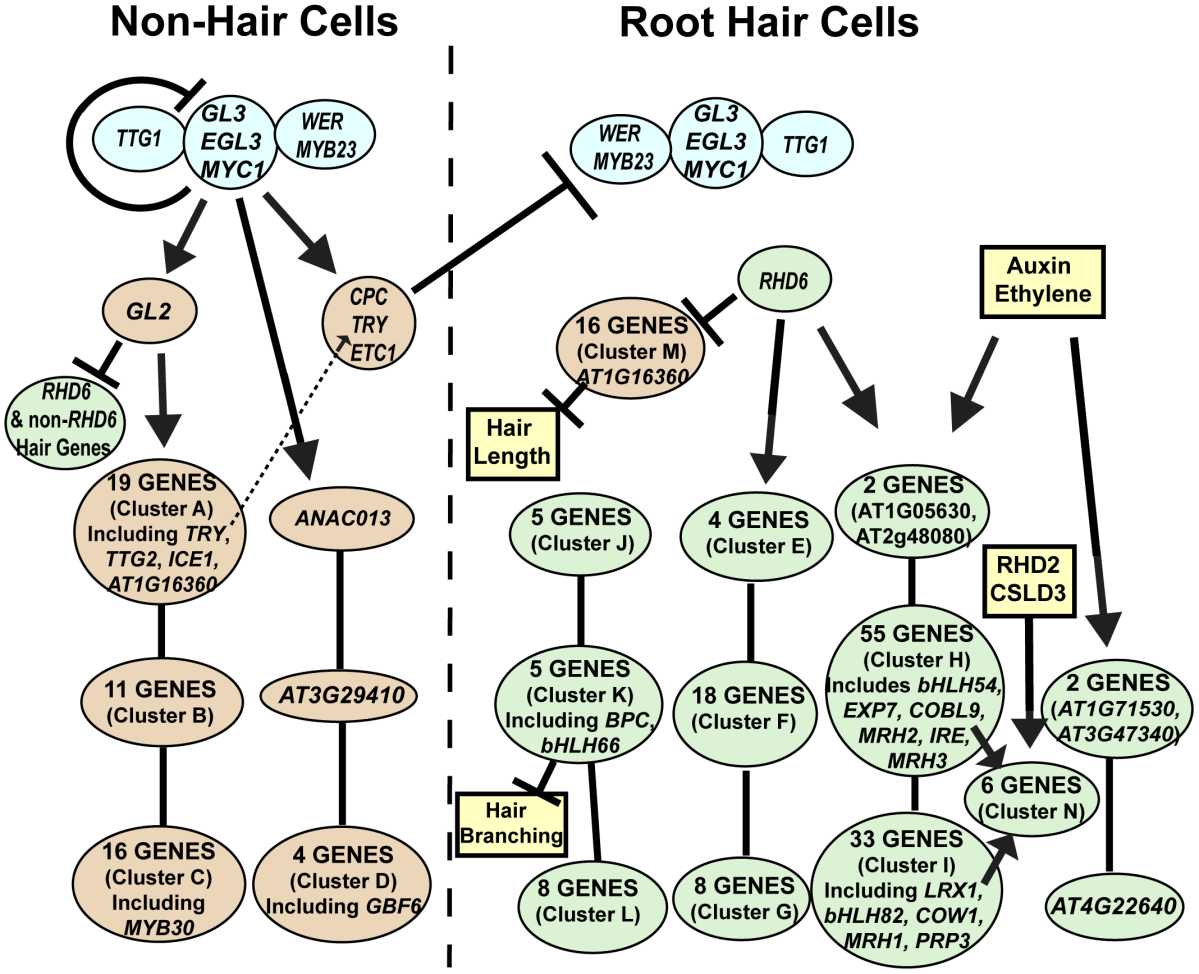
Model of the root epidermal gene network. The predicted transcriptional relationships are shown for the 154 core root hair genes (green), the 54 core non-hair genes (orange), the early acting transcription factors (blue), and other factors not formally part of the network (yellow). The location of genes along the y axis of the figure indicates the relative timing of maximal gene expression during root epidermis development. Genes or gene clusters connected by lines without arrowheads represent genes at a common transcriptional regulatory position but differing in their temporal expression (Zones 1/2, 3/4, and 5/6, from top to bottom). The lists of specific genes in each cluster (A–N) are provided in Table S8, and the GO classes overrepresented in each cluster is given in Table S9.

The root epidermal transcript information obtained from the mutants, hormone treatments, and developmental zone analysis were integrated with known molecular genetic information to construct a gene regulatory network (Figure 8, Tables S8 and S9). This structure was built principally from transcriptional effects (i.e. genes exhibiting the same dependence relationships in knockout or hormone experiments were grouped), and developmental timing information was used to resolve discordant results or to predict likely transcriptional relationships. This depiction emphasizes that, rather than a simple linear pathway, root epidermis differentiation is controlled by branching transcriptional pathways that act coordinately to generate the two cell types.

## Discussion

### The Organization and Logic of the Root Epidermis Gene Regulatory Network

During the past twenty years, traditional molecular genetic analyses have led to the identification and functional characterization of many individual genes that influence root epidermis development in Arabidopsis, making it one of the best characterized cell specification and differentiation processes [65]. Here we exploited these available resources and conducted a comprehensive genome-wide analysis to expand our understanding of the genes involved in this process and to assemble them into a transcriptional regulatory network (Figure 8). The results from this study provide a new view of the composition of the root epidermis gene network, the organization of genes within the network, and the role of plant hormones in modulating root hair formation.

First, this work significantly expands our knowledge of the genes that participate in root epidermis development. Less than one-half of the 1582 genes identified in our basic transcript profiling analysis had been previously associated with root epidermis development in any other genetic or genomic screens. Comparing our 208 core root epidermal genes with other microarray-based root epidermis gene sets [52], [64], [66], [67], [68] shows that our gene set includes a greater fraction of genes expressed at early developmental stages as well as non-hair cell genes (Figure S8; Table S10). This likely reflects the ability of our *WER::GFP*-based FACS method to acquire both types of epidermal cells at all stages of differentiation, as opposed to other approaches that focus on the root hair cell type and at later stages of development.

A general observation from our gene list and the resulting network is that the differentiating non-hair cells and hair cells exhibit distinct gene expression patterns (Figure 8). This is particularly evident in the several instances of gene family member-specific expression; for example, each cell type expresses distinct expansin genes (*EXPA10* in non-hair cells vs *EXPA7* and *EXPA18* in hair cells) and arabinogalactan cell wall proteins (*AGP13* in non-hair cells vs *AGP3* in hair cells). This observation suggests that non-hair cells should not be considered as merely developmentally-arrested hair cells or hair cells that lack a root hair, rather, they arise from a specific gene expression program.

We have also uncovered fundamental features of the organization of the root epidermis network. As expected from their strong mutant phenotypes, the RHD6 and GL2 transcription factors act at pivotal positions in the network, positively regulating a large fraction of genes involved in root hair and non-hair cell differentiation, respectively. However, they do not control all of the genes associated with each of these processes, which leads to multiple branches in the epidermal gene network (Figure 8). In particular, our finding of GL2-dependent and GL2-independent branches of non-hair cell differentiation is consistent with prior speculation, principally derived from observations that *gl2* mutants do not completely convert non-hair cells to hair cells [12], [17], [69]. An unexpected feature of RHD6 and GL2 action in this network is their transcriptional repression of genes associated with the alternate cell types; the *rhd6* mutant alters some non-hair cell transcripts and the *gl2* mutant alters some hair cell transcripts (Figure 8). The strong inverse association between *AT1G16360* expression (a *LEM3*/*CDC50* related non-hair gene in Cluster M) and root hair length in our Bayesian network analysis (Figure 6) provides further support for the importance of RHD6-mediated inhibition of non-hair genes in the differentiating hair cells. Together, these findings suggest a previously unrecognized level of coordinated gene regulation that may be important to ensure robust adoption/differentiation of distinct cell types.

Another general feature of the root epidermal network relates to the lack of evidence for transcriptional feedback from the downstream genes to the early acting transcriptional regulators. Specifically, none of the nine downstream mutants (*cobl9, cow1, csld3, ire1, lrx1, mrh1, mrh2, mrh3, rhd2*) significantly alter the transcript level of any of the *GL2*, *RHD6*, *MYC1*, *TTG2*, *ETC1*, or *TRY* genes. This suggests that, once cell fate is established by the early regulators, there is little feedback regulation (at least at the transcriptional level) by genes acting at late stages. Interestingly, we did discover an exceptional group of six root-hair genes whose transcripts are significantly affected by at least six of the nine downstream gene mutants we examined (shown as Cluster N in Figure 8). Given the robust transcriptional response of these genes to perturbations in root hair morphogenesis genes, they may define a core group of root hair genes that are particularly sensitive to root hair growth abnormalities.

Our analysis of auxin and ethylene treatments provides insight into the molecular basis for their well-known ability to promote root hair formation [39], [42], [43], [51]. We discovered substantial overlap in the root epidermal genes affected by these two distinct hormones (>90% overlap), which suggests a common molecular response of the root epidermal network to each of these, perhaps due to the known linkage between auxin and ethylene biosynthesis in roots (reviewed in [70]). Interestingly, we find that these hormones exclusively affected root hair genes, demonstrating the sensitivity of the root-hair cell type to auxin/ethylene. Given that auxin/ethylene largely affect RHD6-dependent genes (Figure 8), it is possible that RHD6 makes root hair cells more sensitive to these hormones, which might explain why a high exogenous concentration of these hormones induces hair initiation in *rhd6*. Further, it is notable that all of these hormone-responsive genes act downstream of the early transcriptional regulators (Figure 8), which provides support for the view that these plant hormones do not drive cell fate decisions, but they modify the cell differentiation processes that are initially directed by the early regulators.

### A Suite of bHLH Genes Regulates Root Epidermis Development

Among our collection of root epidermal genes, we have shown that a set of bHLH genes participate in distinct phases of root epidermis development. Specifically, we identified genes in three subfamilies of the Arabidopsis bHLH family (subfamilies IIIf, VIIIc, and XI [53]) that are required for epidermal cell fate and/or root hair cell differentiation.

With respect to the bHLH subfamily IIIf, we discovered a new role for one of its members, *MYC1*, in the specification of root epidermal cells. The ectopic hair formation in the *myc1* mutant indicates that MYC1 is required to specify the non-hair cell fate. Our expression and promoter-reporter analyses show that *MYC1* is expressed in the differentiating hair cells and is negatively regulated by WER and positively regulated by CPC/TRY. These characteristics are similar to the *GL3* and *EGL3* genes, two other known genes in the subfamily IIIf [23], [24], and we discovered that *myc1* can enhance the effect of *gl3* or *egl3* on root epidermal patterning. Furthermore, a prior study showed that, like the GL3 and EGL3 proteins, MYC1 can interact with WER and other R2R3 MYB proteins in yeast [71]. Altogether, these findings suggest that MYC1 acts redundantly with GL3 and EGL3 to direct the non-hair cell fate via its expression in the hair cell, and possible movement to the non-hair cells, which may be important for mutual support of neighbor cell fates for robust cell pattern formation [72]. Interestingly, GL3, EGL3, and MYC1 also regulate trichome spacing, though in this process, they are expressed in the same cells (trichomes) that they help to specify [73].

For the bHLH subfamily VIIIc, which includes the root hair initiation gene *RHD6*, we identified *bHLH54*, *bHLH84*, and *bHLH85* as additional genes required for normal root hair formation. Mutations affecting any one of these cause abnormal root hair morphogenesis, suggesting that these genes regulate at least a subset of hair genes. Although we cannot place *bHLH84* and *bHLH85* within our network (because they are not represented on the microarray chip), the *bHLH54* gene appears to encode a relatively late-acting transcriptional regulator of hair cell differentiation (Figure 8). Our findings are consistent with recent work suggesting *bHLH54*/*RSL4* is a regulator of root hair elongation, because it generates longer hairs when overexpressed [66]. Furthermore, transcription from *bHLH54* and *bHLH85* (also known as *RSL2*/*AT4G33880*) was shown to be controlled by RHD6 and the RHD6-related RSL1 [66].

In the bHLH subfamily XI, we discovered that *bHLH66*, *bHLH69*, and *bHLH82* are each involved in root hair formation. The *bHLH66* gene is particularly interesting because its expression is RHD6-independent, and it may therefore control its own branch of the hair differentiation process. Furthermore, this *bHLH66*-dependent pathway appears to have a strong connection to root hair branching, because our Bayesian network analysis showed *bHLH66* expression is the strongest (negative) predictor of hair branching. Interesting, the *bHLH66*, *bHLH69*, and *bHLH82* bHLH genes were found to be functionally similar to a *Lotus japonicus* bHLH (called *Ljrhl1*) that is required for root hair formation [74]. Furthermore, multiple mutants containing at least two of these three caused defects in root hair elongation in Arabidopsis, although single mutants of each of these lacked a visible phenotype [74]. Our ability to observe mutant phenotypes for these single mutant lines in this study may be due to our more quantitative analysis of root hair development (Figure 3).

It is notable that members of the bHLH superfamily are now known to participate at many stages of epidermis development, not only at the early stages of cell specification and hair initiation as previously known. The apparent evolution of the root epidermis differentiation pathway to utilize many different bHLH transcription factors is similar to recent findings in Arabidopsis stomatal development, where a series of bHLHs coordinate cell state transitions during guard cell specification and differentiation [75]. Although the specific bHLH genes employed in these two systems are distinct, the general similarity in the use of bHLHs in root and leaf epidermal development suggests that the sequential action of bHLH proteins to trigger specific cell differentiation events may represent a common strategy for regulating the progression of plant cell differentiation.

### Lessons from the Construction of the Arabidopsis Root Epidermal Gene Network

There are many challenges in the construction of gene regulatory networks. One challenge is to ensure that the correct genes are included as members of the network. In our case, we used two strategies to constrain our gene set to help ensure the included genes are involved in root epidermis cell differentiation. First, we used RNA from *WER::GFP* marked cells via a FACS-based method, which limited the number of cells to those in the developing epidermis and lateral root cap. Although the *WER::GFP* marker is preferentially expressed in the differentiating non-hair cells, it is significantly expressed in the differentiating hair cells as well [17], and our ability to robustly identify previously defined root-hair cell genes in our datasets suggest that GFP accumulation in the root hair cells is sufficient for capture using our cell sorting approach. Further, we required that all of the included genes be regulated by the known early cell fate transcription factors, by demanding significant differential transcript accumulation in all three non-hair fate mutants (*wer myb23*, *gl3 egl3*, and *ttg*) relative to the hair fate mutant (*cpc try*) in two independent labs. Still, it is likely that some genes involved in root epidermal differentiation are not included in our network, due to non-transcriptional regulation, transcript instability during protoplast isolation, or the absence of the gene from the ATH1 gene chip. Indeed, we showed that three genes (*bHLH69*, *bHLH84*, and *bHLH85*) not represented on the ATH1 chip are involved in root epidermis development.

Another difficulty in gene network construction is to properly organize the genes in a manner that reflects their regulatory relationships. A unique aspect of our approach was to use an array of 17 different root epidermal mutant lines with defects in distinct stages of root epidermis development to perturb the network and define the regulatory relationships between the genes. One advantage of this approach is its likely low level of false positives; that is, if a gene's transcript is significantly altered in a given mutant background, it is very likely to be a (direct or indirect) target. In contrast, indirect methods to assess effects on gene transcription (e.g. promoter binding assays or genome-wide ChIP methods) have been reported to yield a high proportion of false positives ([76], [77]. A drawback of the reliance on a mutant-based approach is genetic redundancy, because the presence of a redundantly acting gene prevents the full effect of a mutated gene to be observed. We have some indication that this was problematic in our analysis; the likely redundancy between *MYC1* and the *GL3*/*EGL3* genes probably prevented a complete understanding of the role of MYC1 by analyzing *myc1*-related transcript changes.

A strategy we used to reduce the complexity of the network construction problem was to incorporate temporal gene expression data into the analysis. The principle behind this strategy is that, if expression of gene X generally precedes expression of gene Y during development, then an edge from gene X to gene Y is assumed to be more likely. As a specific example in support of this strategy, we know from biological experiments that the maximal RNA levels for *WER*, *GL3*, and *EGL3* occur earlier [17], [23] than for *RHD6* [41] which itself is earlier than the maximal RNA level for *PRP3* [49], and, indeed, this gene order is consistent with the positions of these genes in the network hierarchy (Figure 8).

The root epidermis system appears to provide a relatively simple gene network that may serve as a model for the construction and analysis of more complex networks in Arabidopsis. In principle, the general approach used here, to define the components and organization of a network via genetic and hormonal perturbation, and incorporating temporal gene expression data, may be generally applicable for other developmental or metabolic networks in Arabidopsis.

## Materials and Methods

### Plant Material and Growth Conditions

Information concerning the *Arabidopsis thaliana* mutant lines used in this study is listed in Table S1. Mutant and transgenic T-DNA lines were obtained from the Arabidopsis Biological Resource Center (ABRC) at Ohio State University. The *WER::GFP* construct and transgenic line have been previously reported [17]. The same *WER::GFP* transgene was incorporated into each of the mutant lines by crossing with a specific *WER::GFP* transgenic line, which contains a single transgene on chromosome five. The bHLH gene designations used here follows the naming convention initially established for this gene family [78]. At least two independent mutants were analyzed for each gene; one each was selected for detailed characterization and is presented here. Each insertion mutant line was verified as homozygous using PCR with primers specific for each gene.

Seeds were surface-sterilized, incubated for two days at 4°C, and sown on MS media solidified with 0.6% agarose under sterile conditions as previously reported [56]. Seedling roots were analyzed for phenotypic characters or used for gene expression analyses after four or five days of growth at 22°C under continuous illumination. Plants were grown to maturity under a photoperiod of 14 hours of light (22°C) and 10 hours of dark (18°C).

### Root Epidermis Analysis

The characterization of root epidermal pattern, root hair length, and root hair branching in the wild-type and mutant lines essentially followed established protocols [36], [47], [69]. Briefly, to characterize the distribution of root-hair cells and non-hair cells, 10 cells in the H position and 10 cells in the N position were assessed in each of 15–20 4-day-old seedling roots for each line. Root hair length was determined by measuring 10 individual hairs from the mature region of each of 30–40 4-day-old seedling roots. Photographs of roots, and a length standard, were used to make measurements using the Scion Image software (www.scioncorp.com). For the analysis of root hair branching, 50 root hairs were examined per root in each of 9 seedlings (450 total hairs). If a root hair possessed a branch, no matter its length or location, it was classified as a branched hair. Each of these analyses was carried out three independent times to minimize the impact of environmental variation.

### Confocal Microscopy

To examine GFP accumulation in seedling roots, 4- or 5-day old seedlings were counterstained with propidium iodide for 5 min and examined with a Zeiss LSM510 Meta confocal microscope with excitation at 488 nm and detection with a 500–530 nm band-path filter for GFP, and with excitation at 543 nm and detection with a 560 nm long-path filter for propidium iodide [17].

### Gene Constructs and Plant Transformation

To assess the expression pattern of *MYC1*, an ER-tagged version of the GFP coding region (mGFP5-ER) was inserted between a 2.7 kb 5′ flanking genomic DNA fragment of *MYC1* and the NOS terminator sequence [79]. Plant transformation was performed by the floral dip method as previously described [80] using the plasmid pCB302 as the binary vector [81].

### Reverse Transcriptase PCR

Total RNA was isolated using TRIzol reagent (Invitrogen, Inc.) from the roots of 4-day-old wild-type (Columbia) and *gl2* mutant seedlings. The RNA was treated with RQ1 DNase (Promega, Inc.), and 2 µg RNA was used for cDNA synthesis with Superscript II-reverse transcriptase (Invitrogen, Inc.) and an oligo (dT)12–18 primer (Invitrogen, Inc.). Equal amounts of cDNA were subjected to standard PCR reactions using primers specific for *ACTIN4* (forward: 5′-GCCGATGGTGAGGATATTCAT-3′; reverse: 5′-CATACCCCTCGTAGATTGGC-3′) and *MYB30* (forward: 5′-ACCAAGAGGGGTCAGCAAATTTCTC-3′; reverse: 5′-CATGTTCTGTGAAATTGCCTCTTTTG-3′) and using a variable number of cycles to establish linear range of product.

### Microarray and Bioinformatics Analyses

Three biological replicates were conducted for each plant line used for microarray gene expression analysis. The four foundational lines (*wer myb23*, *ttg*, *gl3 egl3*, and *cpc try*) were analyzed at both the University of Michigan and Duke University (total of six biological replicates for each line). RNA for the microarrays were obtained from protoplasts derived from differentiating root epidermal cells using a fluorescence-based cell sorting procedure essentially as previously described [45], [82]. Briefly, for each replicate, root tips from approximately 1000 *WER::GFP*-expressing seedlings were pooled, then subjected to cell wall degrading enzymes and GFP-based cell sorting on a BD FACS machine. RNA was extracted using the Qiagen RNeasy Micro Kit and amplified using the Affymetrix Small Sample Labeling Protocol VII and its quality was verified by capillary electrophoresis using a Bioanalyzer 2100 (Agilent). Sample preparation for cDNA preparation, hybridization, and detection to the Arabidopsis ATH1 GeneChip were according to Affymetrix (Santa Clara, CA) protocols. GeneChips were scanned using the Affymetrix 3000 7G GeneChip Scanner with Autoloader. Raw images (CEL format) were generated with Affymetrix GeneChip Operating Software.

The probeset summaries from the Affymetrix ATH1 Arabidopsis microarray data were computed using the RMA (Robust Multichip Average) method with quantile normalization [83]. Custom probeset definitions were used for data preprocessing [84]. All values subsequently analyzed were log_2_ scale expression levels. The multi-way significance analysis of microarrays (SAM) test was arranged to consider the *cpc try* dataset as one (hairless) class and the *gl3 egl3*, *wer myb23*, and *ttg* datasets as another (hairy) class [85]. Scores were analyzed in terms of False Discovery Rate (FDR) q-values [86]. For comparison of microarray datasets from individual genotypes, gene transcript levels were considered to be significantly different at a 5% FDR level and/or p<0.01.

Hierarchical clustering of the genes and samples was performed using the GenePattern suite of tools [87], [88]. GO term enrichment analysis was performed using an online tool at: http://amigo.geneontoloty.org/cgi-bin/amigo/term_enrichment with the TAIR GO annotations (http://www.arabidopsis.org/tools/bulk/go/index.jsp) and application of Fisher's exact test (http://en.wikipedia.org/wiki/Fishers_exact_test). Multidimensional scaling (MDS) of the triplicate sets of microarray data was performed using the MATLAB Statistics Toolbox (http://www.mathworks.com/help/toolbox/stats/mdscale.html) using data from the 208 core root epidermal genes.

### Bayesian Network Analysis

The Bayesian network analysis was performed using the Pebl software environment datasets (http://jmlr.csail.mit.edu/papers/v10/shah09a.html) [89], [90]. All of the microarray datasets from wild-type and knockout mutants (n = 66) were used to define interactions in a 219 node network, which included the 208 core root epidermal genes, 7 genes representing knockout mutants but not part of the core genes, 2 hormone treatments (ACC and IAA), and 2 morphological characters (hair length and degree of hair branching). The gene expression data was discretized to three states (low, medium, and high), the hormone and branching nodes were discretized into two states, and the hair length node was discretized into four states. The genes (nodes) that were knocked out in a particular sample were made explicit to the network learner. The phenotype nodes were constrained to be “child” nodes and the hormone treatments as “parent” nodes in the network; the remaining nodes were unconstrained. A combination of “greedy” and “simulated annealing” (SA) learners was used to learn the network structure from local environments. Several thousand hours of computation on an Apple G5 machine were used to examine more than 10^9^ random networks and find edges appearing in at least 40% of the 42,000 highest scoring networks. This required percentage was chosen because a higher percentage led to a large number of small networks, whereas a lower percentage led to a highly connected network. The connections in the final networks were identified using a consensus approach across all the top scoring networks.

The integrated regulatory network (presented in Figure 8) was primarily generated from the transcriptome perturbation experiments (using mutants and hormone treatments) and the temporal expression data (using sections along the longitudinal axis of the root). Gene clusters were defined by genes that shared a common transcriptional regulatory position (based on their response in the perturbation experiments) and a common zone of temporal expression. To simplify this model, only three developmental zones were used, by combining genes expressed in zones 1 and 2, zones 3 and 4, and zones 5 and 6 (as defined in Figure 7). The lists of specific genes in each cluster (A–N) are provided in Table S8, and the GO classes overrepresented in each cluster is given in Table S9.

### Microarray Data Accession Numbers

The microarray-based data generated in this study has been deposited in the Gene Expression Omnibus (http://www.ncbi.nim.nih.gov/geo/) under the series record GSE30547 and include accession numbers GSM757819 through GSM757893 containing data from 75 individual samples.

## Supporting Information

Figure S1 Phenotypic characterization of Arabidopsis mutant lines used for microarray gene expression analysis. (A) Images of individual root hairs from wild-type and mutant lines. The hairless *cpc try* and *rhd6* mutants are not included. Bar = 20 µm. (B) Percent of branched root hairs produced in mutant roots. For each line, 450–800 root hairs were examined. Mutants marked with an asterisk exhibited a significantly greater fraction of branched hairs than the wild type (p<0.005). The hairless *cpc try* mutant was not analyzed. (C) Pattern of root epidermal cell types in the wild-type and mutant lines. The percent of root-hair cells (gray bars) and the percent of non-hair cells (open bars) located in the H cell position and the N cell position are indicated. The *bHLH66* line is not included in panels B and C, because these data are presented in Figure 3.
(TIF)

Figure S2 Expression of the *WER::GFP* transgene in the root epidermis of the Arabidopsis wild-type and mutant lines used in this study. Confocal microscope images at a median longitudinal view (left) and at an epidermal surface view (right) show that each of these exhibit a similar level and pattern of GFP accumulation. All images are at the same magnification. Bar = 50 µm.
(TIF)

Figure S3 Cell type pattern in the root epidermis of bHLH mutants. The percent root hair cells and non-hair cells in the H and the N cell files was determined. Asterisks indicate lines with statistically significant differences (p<0.05).
(TIF)

Figure S4 Effect of *gl2* on *MYB30* RNA accumulation. RT-PCR was used to assess the relative level of *MYB30* RNA in the seedling root of WT and *gl2* mutants. *ACTIN4* was used as a loading control (left lanes) and control samples lacking reverse transcriptase were also analyzed (right lanes). This image is a representative result from three separate trials.
(TIF)

Figure S5 Heirarchical clustering of 208 core root epidermal genes from three replicates of ATH1 microarray assays for each of 18 mutant and wild-type lines (54 chips total). This represents an expansion of the data presented in Figure 4C, which depicts the mean expression values. The specific microarray sample numbers are indicated with the genotypes.
(TIF)

Figure S6 Multidimensional scaling analysis of the 21 microarray datasets. Transcript accumulation data for the 208 core epidermal genes was analyzed from the 21 mutants, wild-type, and hormone treated lines. For each dataset, the round symbols indicate each of the three replicates and the square symbol represents the mean of the three replicates.
(TIF)

Figure S7 Root hair length in root epidermis mutant lines. The number of root hairs measured within each length interval is indicated. For both A and B, the wild-type is shown by the blue bars.
(TIF)

Figure S8 Genes present in the 208 gene list and five other root transcriptome gene lists. For each list, the genes overlapping with this study's 208 gene list is indicated. Green = hair genes. Yellow = non-hair genes. W = Won et al. [52], Y = Yi et al. [66], J = Jones et al. [67], D = Deal et al. [68], B = Brady et al. [64].
(TIF)

Table S1 Arabidopsis root epidermis mutants analyzed in this study.
(DOC)

Table S2 List of 1,582 root epidermis genes differentially expressed in the root epidermis of hairy versus hairless mutant lines.
(DOC)

Table S3 List of significant Gene Ontology classes among 1,582 genes differentially expressed in the hairy versus hairless mutant lines.
(DOCX)

Table S4 List of 208 core root epidermal genes.
(DOCX)

Table S5 Genes significantly affected by mutations in downstream root epidermal genes.
(XLSX)

Table S6 Gene Ontology (GO) terms overrepresented among the genes significantly affected by mutations in downstream genes.
(DOCX)

Table S7 Mean root hair length in root epidermis mutants and wild type.
(DOCX)

Table S8 Composition of gene clusters in the root epidermal network.
(XLSX)

Table S9 Gene Ontology (GO) terms overrepresented among genes in the gene clusters in Figure 8.
(DOCX)

Table S10 Overlap in gene lists identified in this study and other microarray studies. (A) Overlap with Won et al List [52]. (B) Overlap with the Yi et al List [66]. (C) Overlap with Jones et al List [67]. (D) Overlap with Deal et al List [68]. (E) Overlap with Brady et al List [64].
(XLSX)
