# Supplementary figures and images for: A Gene Regulatory Network for Root Epidermis Cell Differentiation in Arabidopsis
Source: PLoS Genet. 2012 Jan 12;8(1):e1002446. doi: 10.1371/journal.pgen.1002446 (PMC3257299; doi:10.1371/journal.pgen.1002446)

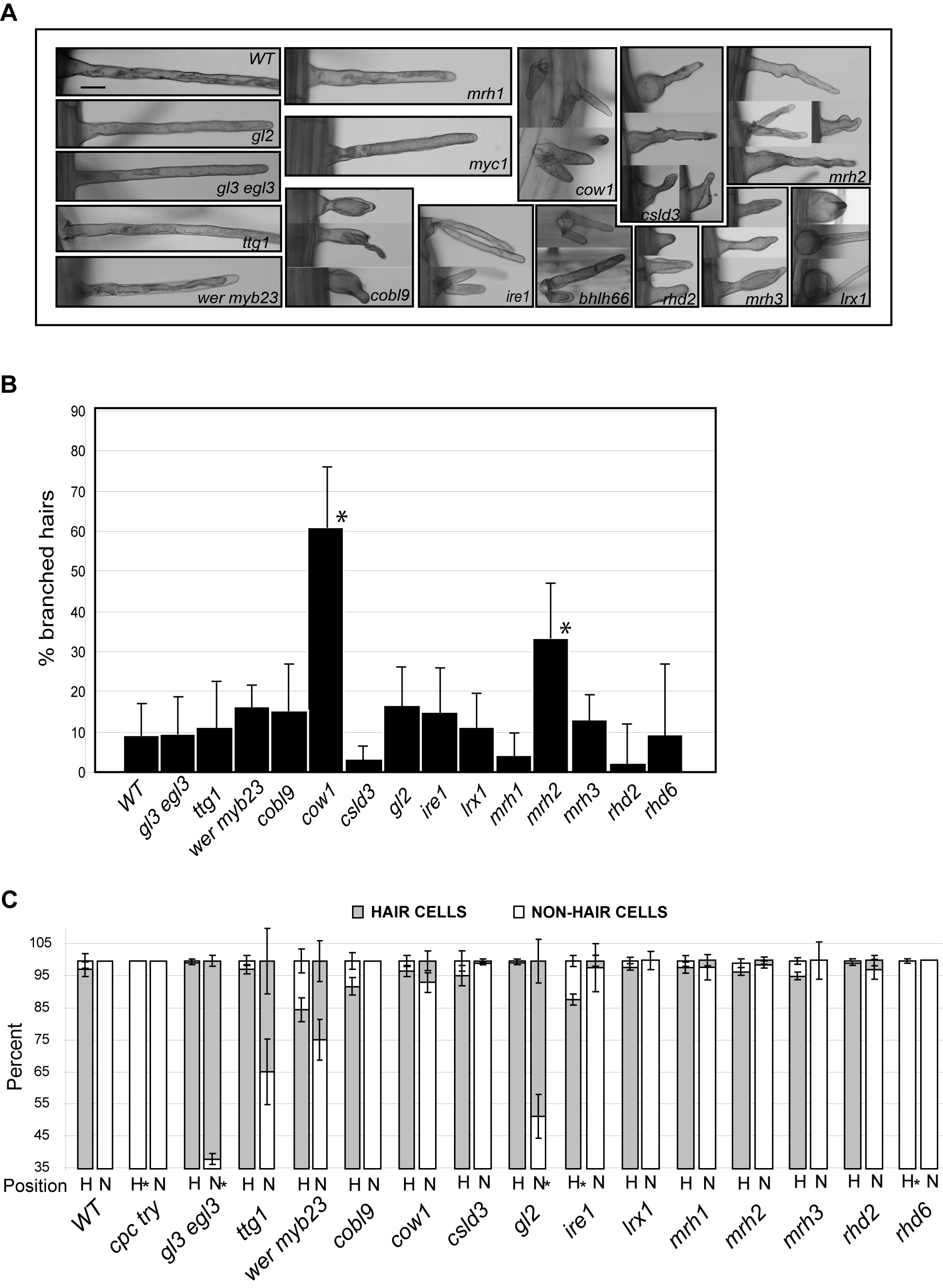

Supplement: Figure S1 — Phenotypic characterization of Arabidopsis mutant lines used for microarray gene expression analysis. (A) Images of individual root hairs from wild-type and mutant lines. The hairless cpc try and rhd6 mutants are not included. Bar = 20 µm. (B) Percent of branched root hairs produced in mutant roots. For each line, 450–800 root hairs were examined. Mutants marked with an asterisk exhibited a significantly greater fraction of branched hairs than the wild type (p<0.005). The hairless cpc try mutant was not analyzed. (C) Pattern of root epidermal cell types in the wild-type and mutant lines. The percent of root-hair cells (gray bars) and the percent of non-hair cells (open bars) located in the H cell position and the N cell position are indicated. The bHLH66 line is not included in panels B and C, because these data are presented in Figure 3. (TIF) [file pgen.1002446.s001.tif]

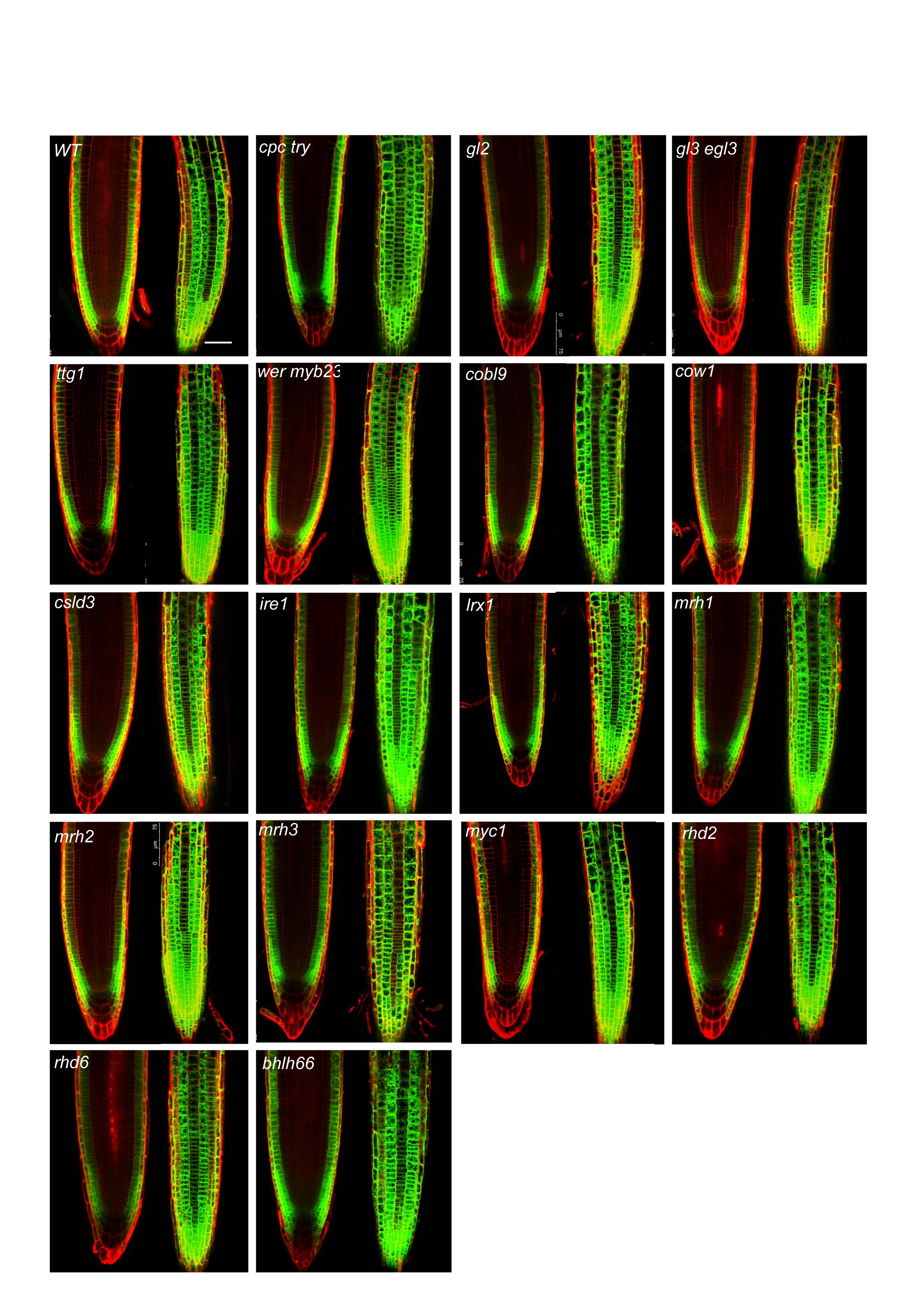

Supplement: Figure S2 — Expression of the WER::GFP transgene in the root epidermis of the Arabidopsis wild-type and mutant lines used in this study. Confocal microscope images at a median longitudinal view (left) and at an epidermal surface view (right) show that each of these exhibit a similar level and pattern of GFP accumulation. All images are at the same magnification. Bar = 50 µm. (TIF) [file pgen.1002446.s002.tif]

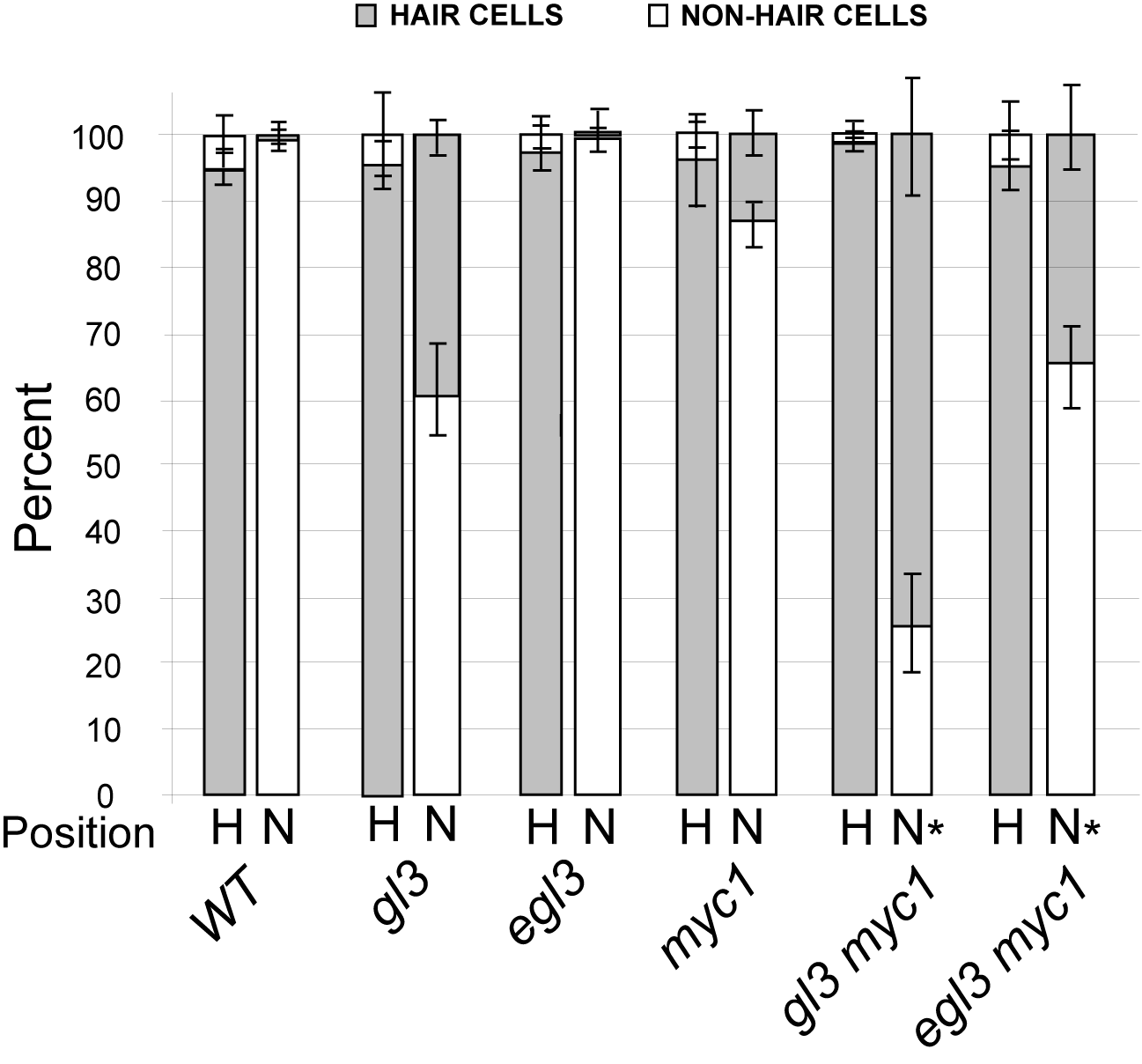

Supplement: Figure S3 — Cell type pattern in the root epidermis of bHLH mutants. The percent root hair cells and non-hair cells in the H and the N cell files was determined. Asterisks indicate lines with statistically significant differences (p<0.05). (TIF) [file pgen.1002446.s003.tif]

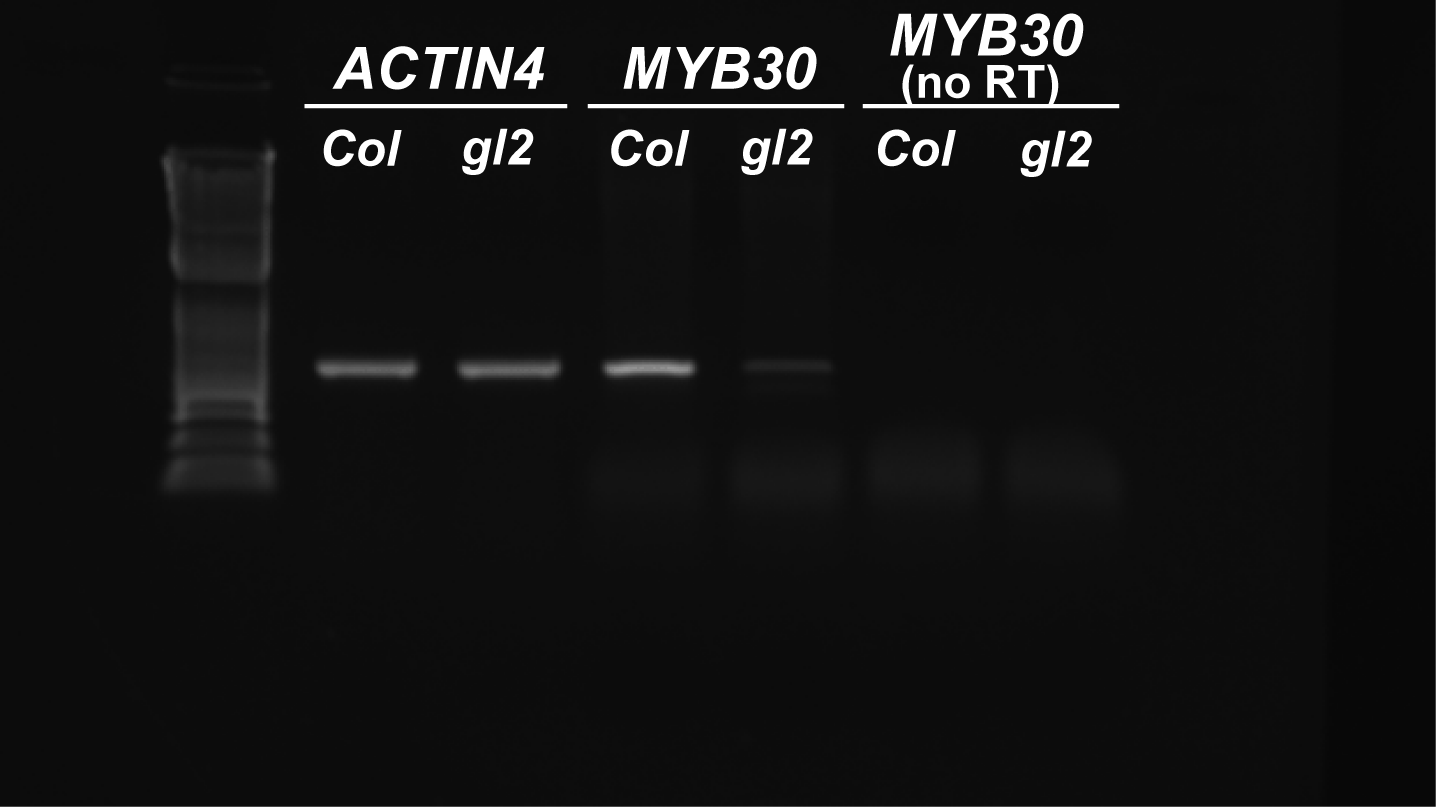

Supplement: Figure S4 — Effect of gl2 on MYB30 RNA accumulation. RT-PCR was used to assess the relative level of MYB30 RNA in the seedling root of WT and gl2 mutants. ACTIN4 was used as a loading control (left lanes) and control samples lacking reverse transcriptase were also analyzed (right lanes). This image is a representative result from three separate trials. (TIF) [file pgen.1002446.s004.tif]

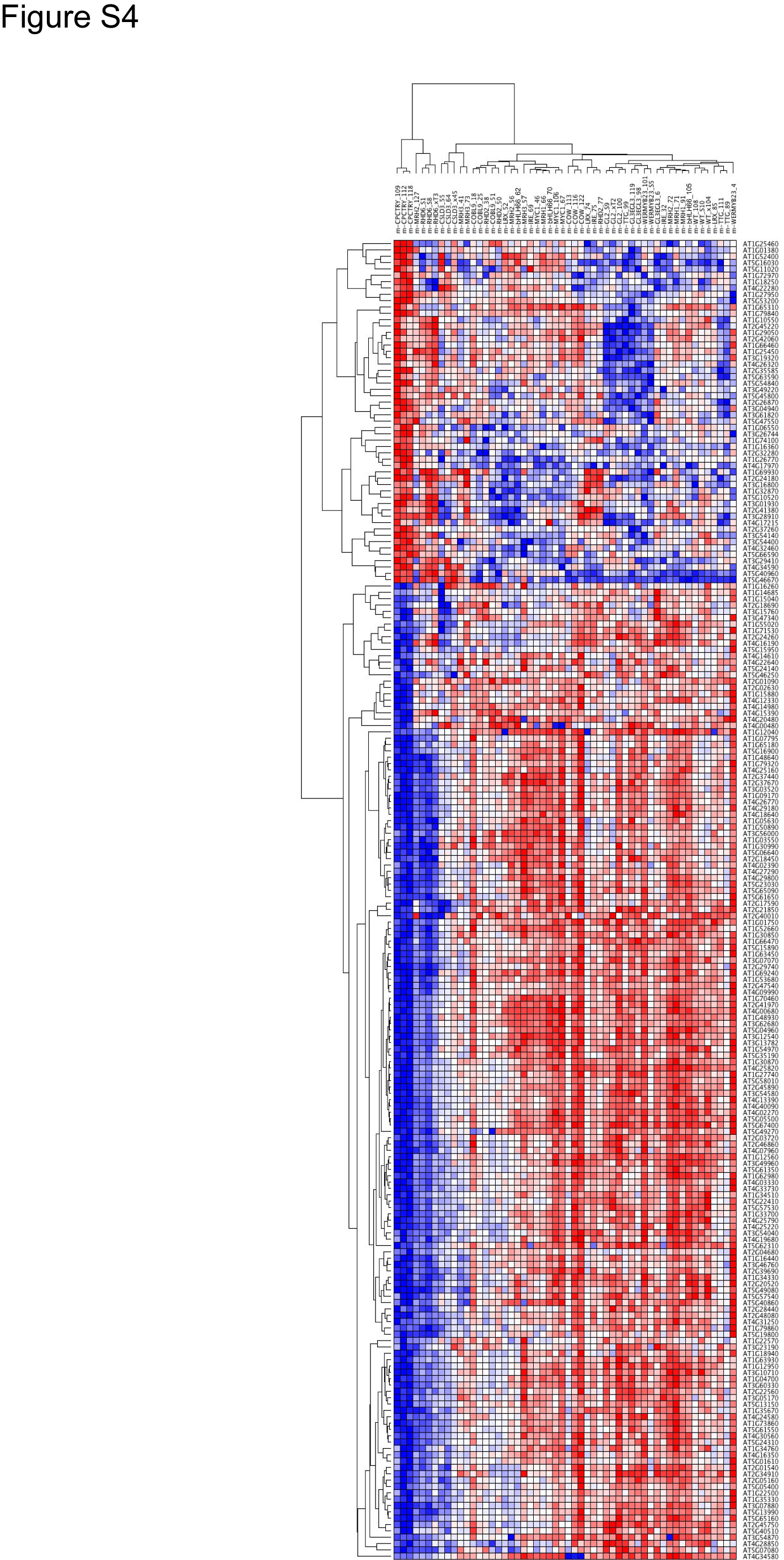

Supplement: Figure S5 — Heirarchical clustering of 208 core root epidermal genes from three replicates of ATH1 microarray assays for each of 18 mutant and wild-type lines (54 chips total). This represents an expansion of the data presented in Figure 4C, which depicts the mean expression values. The specific microarray sample numbers are indicated with the genotypes. (TIF) [file pgen.1002446.s005.tif]

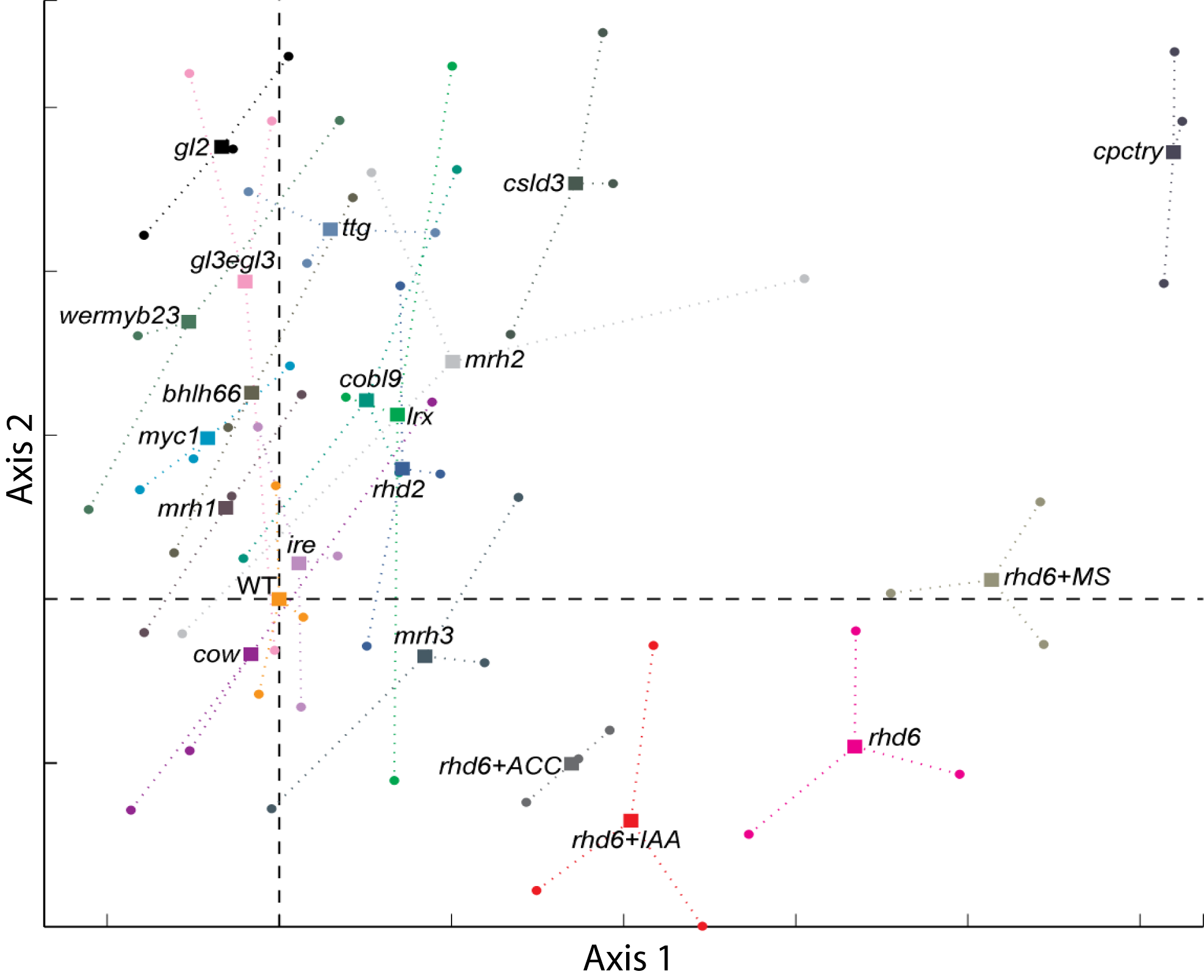

Supplement: Figure S6 — Multidimensional scaling analysis of the 21 microarray datasets. Transcript accumulation data for the 208 core epidermal genes was analyzed from the 21 mutants, wild-type, and hormone treated lines. For each dataset, the round symbols indicate each of the three replicates and the square symbol represents the mean of the three replicates. (TIF) [file pgen.1002446.s006.tif]

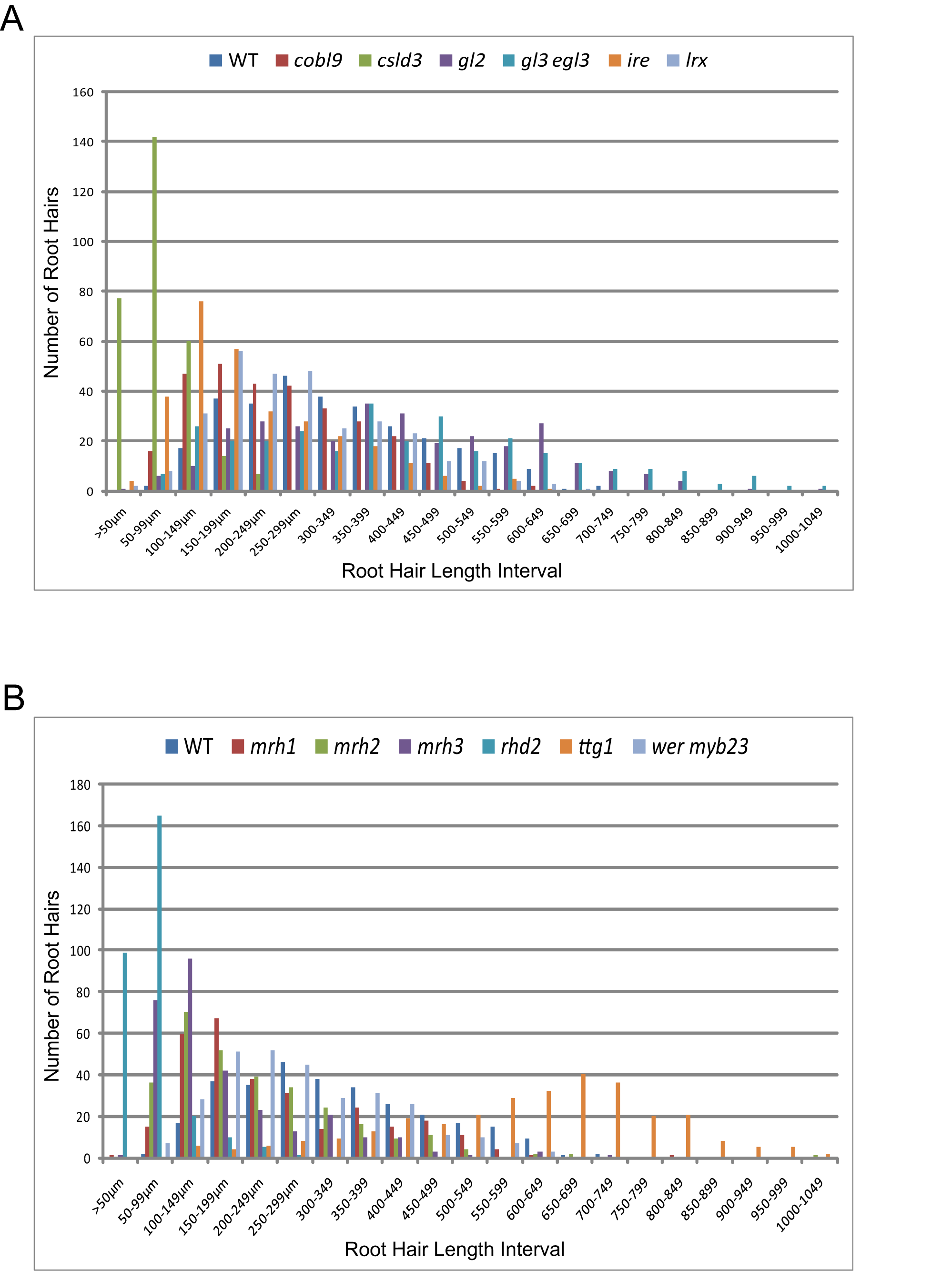

Supplement: Figure S7 — Root hair length in root epidermis mutant lines. The number of root hairs measured within each length interval is indicated. For both A and B, the wild-type is shown by the blue bars. (TIF) [file pgen.1002446.s007.tif]

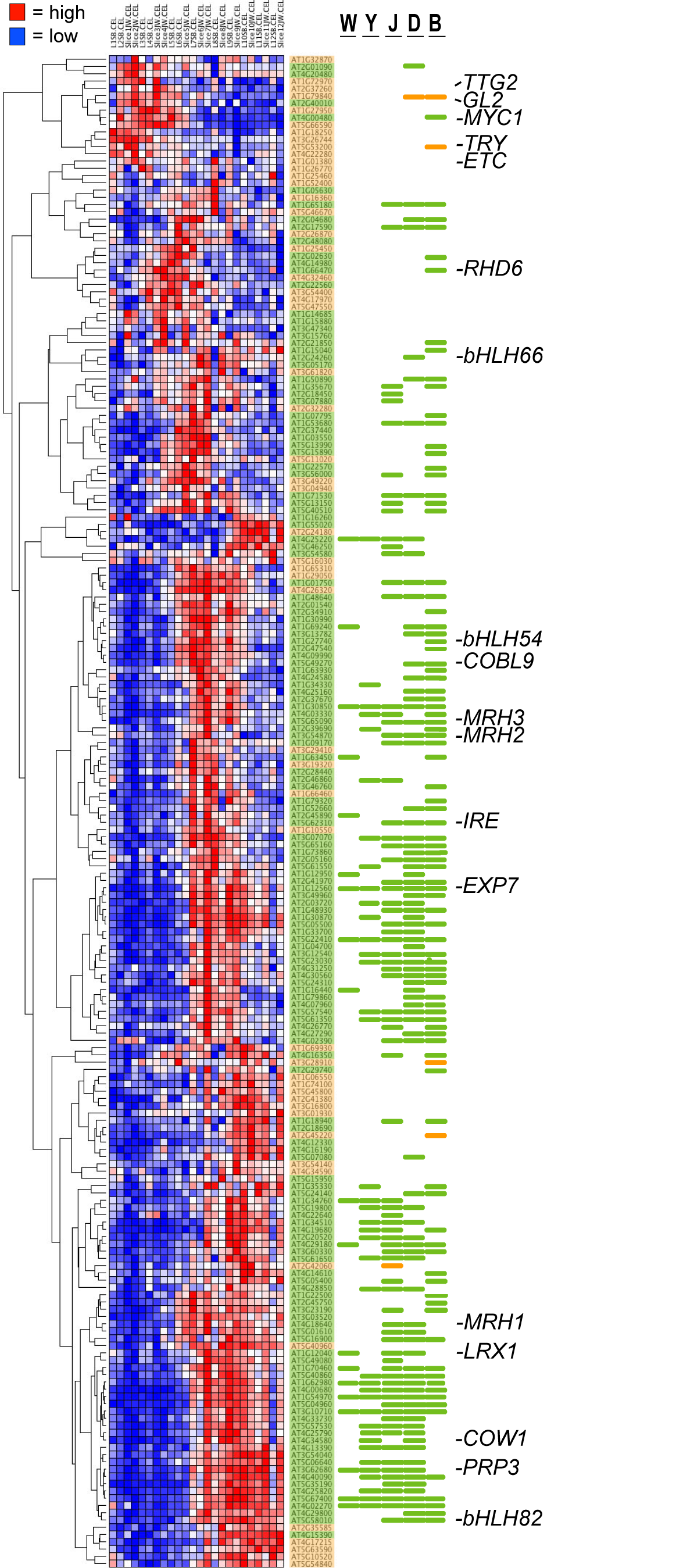

Supplement: Figure S8 — Genes present in the 208 gene list and five other root transcriptome gene lists. For each list, the genes overlapping with this study's 208 gene list is indicated. Green = hair genes. Yellow = non-hair genes. W = Won et al. [52], Y = Yi et al. [66], J = Jones et al. [67], D = Deal et al. [68], B = Brady et al. [64]. (TIF) [file pgen.1002446.s008.tif]
