# Supplementary material for: A Gene Regulatory Network for Root Epidermis Cell Differentiation in Arabidopsis
Source: PLoS Genet. 2012 Jan 12;8(1):e1002446. doi: 10.1371/journal.pgen.1002446 (PMC3257299; doi:10.1371/journal.pgen.1002446)
Supplement: Table S1 — Arabidopsis root epidermis mutants analyzed in this study. (DOC) [file pgen.1002446.s009.doc]

**Table S1.** Arabidopsis Root Epidermis Mutants Analyzed in this Study

| **Mutant Line** | **AGI**  **(Gene Name)** | **Nature of Mutation** * | **Reference** |
| --- | --- | --- | --- |
| *bhlh54-1* | At1g27740  (*bHLH54*) | T-DNA insertion in transcribed region (CS168205) | [1] |
| *bhlh66-1* | At2g24260  (*bHLH66*) | T-DNA insertion in transcribed region (SALK 006430) | [2] |
| *bhlh84-1* | At2g14760  (*bHLH84*) | T-DNA insertion in transcribed region (SALK 064296c) | This Study |
| *bhlh85-1* | At4g33880  (*bHLH85*) | T-DNA insertion in transcribed region (CS874457) | This Study |
| *bhlh69-1* | At4g30980  (*bHLH69*) | T-DNA insertion in transcribed region (SALK 029317c) | This Study |
| *bhlh82-1* | At5g58010  (*bHLH82*) | T-DNA insertion near 5’ end of transcribed region (SALK 015021) | This Study |
| *cobl9-1* | At5g49270 (*COBL9*) | T-DNA insertion in transcribed region (SALK 099933c) | [3] |
| *cow1-3* | At4g34580 (*COW1*) | T-DNA insertion in transcribed region (SALK 002124) | [4] |
| *cpc-1*  *try-82* | At2g46410  (*CPC*)  At5g53200  (*TRY*) | *cpc-1*: T-DNA insertion in transcribed region (CS6399)  *try-82*: base substitution causing nonsense mutation | *cpc-1* : [5]  *try-82* : [6,7] |
| *csld3-1* | At3g03050 (*CSLD3*) | T-DNA insertion in transcribed region (CS899) | [8] |
| *gl2-1* | At1g79840 (*GL2*) | Small deletion causing frameshift mutation (CS65) | [9,10] |
| *gl3-1*  *egl3-1* | At5g41315(*GL3*)  At1g63650 (*EGL3*) | *gl3-1*: base substitution causing truncated protein  *egl3-1*: base substitution causing nonsense mutation | *gl3-1*: [9] [11] *egl3-1*: [12] |
| *ire1-2* | At5g62310  (*IRE1*) | T-DNA insertion in transcribed region (SALK 044925c) | [13] |
| *lrx1-4* | At1g12040  (*LRX1*) | T-DNA insertion near 5’ end of the transcribed region (SALK 057038) | [14] |
| *mrh1-1* | At4g18640  (*MRH1*) | T-DNA insertion in transcribed region (SALK 004879) | [3]Jones et al Plant Journal 2006 |
| *mrh2-1* | At3g54870  (*MRH2*) | T-DNA insertion in transcribed region (SALK 035063c) | [3] |
| *mrh3-3* | At5g65090  (*MRH3*) | EMS-induced mutation (CS25249) | [3]; [15] |
| *myc1-1* | At4g00480  (*MYC1*) | T-DNA insertion in transcribed region (SALK 057388) | This Study |
| *rhd2-1* | At5g51060  (*RHD2*) | Base substitution causing nonsense mutation (CS2259) | [16] |
| *rhd6-1* | At1g66470  (*RHD6*) | T-DNA insertion (CS6347) | [17] |
| *ttg1-13* | At5g24520  (*TTG1*) | Fast neutron-induced deletion | [18] |
| *wer-1*  *myb23-1* | At5g14750  (*WER*)  At5g40330  (*MYB23*) | *wer-1*: EMS-induced nonsense mutation (CS6349)  *myb23-1*: T-DNA insertion in transcribed region (SALK 048592) | *wer-1*: [19]  *myb23-1*:[20] |

* The Arabidopsis stock center number or SALK number is indicated in parentheses.

REFERENCES

1. Yi K, Menand B, Bell E, Dolan L (2010) A basic helix-loop-helix transcription factor controls cell growth and size in root hairs. Nat Genet 42: 264-267.

2. Karas B, Amyot L, Johansen C, Sato S, Tabata S, et al. (2009) Conservation of lotus and Arabidopsis basic helix-loop-helix proteins reveals new players in root hair development. Plant Physiol 151: 1175-1185.

3. Jones MA, Raymond MJ, Smirnoff N (2006) Analysis of the root-hair morphogenesis transcriptome reveals the molecular identity of six genes with roles in root-hair development in Arabidopsis. Plant J 45: 83-100.

4. Grierson CS, Roberts K, Feldmann KA, Dolan L (1997) The COW1 locus of arabidopsis acts after RHD2, and in parallel with RHD3 and TIP1, to determine the shape, rate of elongation, and number of root hairs produced from each site of hair formation. Plant Physiol 115: 981-990.

5. Wada T, Tachibana T, Shimura Y, Okada K (1997) Epidermal cell differentiation in Arabidopsis determined by a Myb homolog, CPC. Science 277: 1113-1116.

6. Hulskamp M, Misra S, Jurgens G (1994) Genetic dissection of trichome cell development in Arabidopsis. Cell 76: 555-566.

7. Schellmann S, Schnittger A, Kirik V, Wada T, Okada K, et al. (2002) TRIPTYCHON and CAPRICE mediate lateral inhibition during trichome and root hair patterning in Arabidopsis. Embo J 21: 5036-5046.

8. Wang X, Cnops G, Vanderhaeghen R, De Block S, Van Montagu M, et al. (2001) AtCSLD3, a cellulose synthase-like gene important for root hair growth in arabidopsis. Plant Physiol 126: 575-586.

9. Koornneef M, Dellaert LW, van der Veen JH (1982) EMS- and radiation-induced mutation frequencies at individual loci in Arabidopsis thaliana (L.) Heynh. Mutat Res 93: 109-123.

10. Rerie WG, Feldmann KA, Marks MD (1994) The GLABRA2 gene encodes a homeo domain protein required for normal trichome development in Arabidopsis. Genes Dev 8: 1388-1399.

11. Payne CT, Zhang F, Lloyd AM (2000) GL3 encodes a bHLH protein that regulates trichome development in arabidopsis through interaction with GL1 and TTG1. Genetics 156: 1349-1362.

12. Zhang F, Gonzalez A, Zhao M, Payne CT, Lloyd A (2003) A network of redundant bHLH proteins functions in all TTG1-dependent pathways of Arabidopsis. Development 130: 4859-4869.

13. Oyama T, Shimura Y, Okada K (2002) The IRE gene encodes a protein kinase homologue and modulates root hair growth in Arabidopsis. Plant J 30: 289-299.

14. Baumberger N, Ringli C, Keller B (2001) The chimeric leucine-rich repeat/extensin cell wall protein LRX1 is required for root hair morphogenesis in Arabidopsis thaliana. Genes Dev 15: 1128-1139.

15. Ringli C, Baumberger N, Keller B (2005) The Arabidopsis root hair mutants der2-der9 are affected at different stages of root hair development. Plant Cell Physiol 46: 1046-1053.

16. Schiefelbein JW, Somerville C (1990) Genetic Control of Root Hair Development in Arabidopsis thaliana. Plant Cell 2: 235-243.

17. Masucci JD, Schiefelbein JW (1994) The rhd6 Mutation of Arabidopsis thaliana Alters Root-Hair Initiation through an Auxin- and Ethylene-Associated Process. Plant Physiol 106: 1335-1346.

18. Larkin JC, Walker JD, Bolognesi-Winfield AC, Gray JC, Walker AR (1999) Allele-specific interactions between ttg and gl1 during trichome development in Arabidopsis thaliana. Genetics 151: 1591-1604.

19. Lee MM, Schiefelbein J (1999) WEREWOLF, a MYB-related protein in Arabidopsis, is a position-dependent regulator of epidermal cell patterning. Cell 99: 473-483.

20. Kirik V, Lee MM, Wester K, Herrmann U, Zheng Z, et al. (2005) Functional diversification of MYB23 and GL1 genes in trichome morphogenesis and initiation. Development 132: 1477-1485.
