# Supplementary material for: A Gene Regulatory Network for Root Epidermis Cell Differentiation in Arabidopsis
Source: PLoS Genet. 2012 Jan 12;8(1):e1002446. doi: 10.1371/journal.pgen.1002446 (PMC3257299; doi:10.1371/journal.pgen.1002446)
Supplement: Table S2 — List of 1,582 root epidermis genes differentially expressed in the root epidermis of hairy versus hairless mutant lines. (DOC) [file pgen.1002446.s010.doc]

**Table S2.** List of 1582 Root Epidermis Genes Differentially Expressed in the Root Epidermis of Hairy vs. Hairless Mutant Lines

| AT1G01220 |
| --- |
| AT1G01380 |
| AT1G01660 |
| AT1G01750 |
| AT1G01780 |
| AT1G02280 |
| AT1G02330 |
| AT1G03550 |
| AT1G03750 |
| AT1G03790 |
| AT1G03840 |
| AT1G03950 |
| AT1G04040 |
| AT1G04120 |
| AT1G04150 |
| AT1G04160 |
| AT1G04200 |
| AT1G04520 |
| AT1G04550 |
| AT1G04680 |
| AT1G04700 |
| AT1G05020 |
| AT1G05030 |
| AT1G05200 |
| AT1G05210 |
| AT1G05260 |
| AT1G05320 |
| AT1G05360 |
| AT1G05510 |
| AT1G05630 |
| AT1G05690 |
| AT1G05710 |
| AT1G05790 |
| AT1G05810 |
| AT1G05990 |
| AT1G06010 |
| AT1G06020 |
| AT1G06550 |
| AT1G07040 |
| AT1G07485 |
| AT1G07610 |
| AT1G07690 |
| AT1G07750 |
| AT1G07795 |
| AT1G07960 |
| AT1G08190 |
| AT1G08230 |
| AT1G08250 |
| AT1G08340 |
| AT1G08350 |
| AT1G08460 |
| AT1G08650 |
| AT1G08800 |
| AT1G08880 |
| AT1G08920 |
| AT1G08990 |
| AT1G09090 |
| AT1G09155 |
| AT1G09170 |
| AT1G09370 |
| AT1G09570 |
| AT1G09575 |
| AT1G09600 |
| AT1G09750 |
| AT1G09850 |
| AT1G09910 |
| AT1G10050 |
| AT1G10150 |
| AT1G10400 |
| AT1G10550 |
| AT1G10730 |
| AT1G10880 |
| AT1G10900 |
| AT1G10940 |
| AT1G10990 |
| AT1G11160 |
| AT1G11540 |
| AT1G11580 |
| AT1G11660 |
| AT1G12040 |
| AT1G12150 |
| AT1G12400 |
| AT1G12470 |
| AT1G12530 |
| AT1G12550 |
| AT1G12560 |
| AT1G12640 |
| AT1G12845 |
| AT1G12950 |
| AT1G12960 |
| AT1G13180 |
| AT1G13195 |
| AT1G13830 |
| AT1G13950 |
| AT1G14040 |
| AT1G14220 |
| AT1G14280 |
| AT1G14310 |
| AT1G14340 |
| AT1G14360 |
| AT1G14685 |
| AT1G15020 |
| AT1G15040 |
| AT1G15100 |
| AT1G15290 |
| AT1G15460 |
| AT1G15490 |
| AT1G15530 |
| AT1G15710 |
| AT1G15880 |
| AT1G16210 |
| AT1G16260 |
| AT1G16360 |
| AT1G16440 |
| AT1G16520 |
| AT1G16560 |
| AT1G16570 |
| AT1G16590 |
| AT1G16850 |
| AT1G17020 |
| AT1G17140 |
| AT1G17160 |
| AT1G17170 |
| AT1G17210 |
| AT1G17330 |
| AT1G17340 |
| AT1G17360 |
| AT1G17430 |
| AT1G17890 |
| AT1G18250 |
| AT1G18260 |
| AT1G18360 |
| AT1G18410 |
| AT1G18590 |
| AT1G18650 |
| AT1G18830 |
| AT1G18890 |
| AT1G18940 |
| AT1G19010 |
| AT1G19200 |
| AT1G19230 |
| AT1G19250 |
| AT1G19290 |
| AT1G19360 |
| AT1G19440 |
| AT1G19650 |
| AT1G19700 |
| AT1G19900 |
| AT1G20090 |
| AT1G20770 |
| AT1G20870 |
| AT1G21360 |
| AT1G21540 |
| AT1G21550 |
| AT1G21590 |
| AT1G21640 |
| AT1G21730 |
| AT1G21980 |
| AT1G22500 |
| AT1G22570 |
| AT1G22610 |
| AT1G22740 |
| AT1G22910 |
| AT1G23040 |
| AT1G23180 |
| AT1G23190 |
| AT1G23340 |
| AT1G23440 |
| AT1G23540 |
| AT1G23700 |
| AT1G23750 |
| AT1G23870 |
| AT1G23880 |
| AT1G23960 |
| AT1G24320 |
| AT1G24340 |
| AT1G24390 |
| AT1G24560 |
| AT1G24600 |
| AT1G24620 |
| AT1G24735 |
| AT1G25240 |
| AT1G25320 |
| AT1G25450 |
| AT1G25460 |
| AT1G25500 |
| AT1G26270 |
| AT1G26300 |
| AT1G26770 |
| AT1G26900 |
| AT1G27140 |
| AT1G27300 |
| AT1G27570 |
| AT1G27740 |
| AT1G27910 |
| AT1G27950 |
| AT1G27980 |
| AT1G28090 |
| AT1G28330 |
| AT1G28360 |
| AT1G28400 |
| AT1G28440 |
| AT1G29050 |
| AT1G29120 |
| AT1G29180 |
| AT1G29230 |
| AT1G29395 |
| AT1G30455 |
| AT1G30640 |
| AT1G30850 |
| AT1G30870 |
| AT1G30900 |
| AT1G30990 |
| AT1G31650 |
| AT1G31750 |
| AT1G31930 |
| AT1G32260 |
| AT1G32340 |
| AT1G32870 |
| AT1G32950 |
| AT1G33250 |
| AT1G33670 |
| AT1G33700 |
| AT1G33790 |
| AT1G33800 |
| AT1G34120 |
| AT1G34330 |
| AT1G34510 |
| AT1G34540 |
| AT1G34760 |
| AT1G35220 |
| AT1G35330 |
| AT1G35670 |
| AT1G35730 |
| AT1G36060 |
| AT1G43670 |
| AT1G44090 |
| AT1G44100 |
| AT1G44510 |
| AT1G44830 |
| AT1G45130 |
| AT1G45545 |
| AT1G46264 |
| AT1G47270 |
| AT1G47290 |
| AT1G47410 |
| AT1G47710 |
| AT1G48280 |
| AT1G48480 |
| AT1G48500 |
| AT1G48598 |
| AT1G48600 |
| AT1G48640 |
| AT1G48780 |
| AT1G48930 |
| AT1G49030 |
| AT1G49110 |
| AT1G49560 |
| AT1G49780 |
| AT1G50360 |
| AT1G50420 |
| AT1G50460 |
| AT1G50890 |
| AT1G50900 |
| AT1G50930 |
| AT1G51420 |
| AT1G51830 |
| AT1G51840 |
| AT1G51860 |
| AT1G51880 |
| AT1G51940 |
| AT1G52080 |
| AT1G52240 |
| AT1G52400 |
| AT1G52410 |
| AT1G52580 |
| AT1G52660 |
| AT1G53050 |
| AT1G53070 |
| AT1G53165 |
| AT1G53490 |
| AT1G53680 |
| AT1G53860 |
| AT1G53920 |
| AT1G53980 |
| AT1G54450 |
| AT1G54820 |
| AT1G54940 |
| AT1G54970 |
| AT1G55020 |
| AT1G55290 |
| AT1G55360 |
| AT1G55430 |
| AT1G56020 |
| AT1G56080 |
| AT1G56170 |
| AT1G56190 |
| AT1G56230 |
| AT1G56540 |
| AT1G56580 |
| AT1G56660 |
| AT1G56680 |
| AT1G58200 |
| AT1G59700 |
| AT1G59725 |
| AT1G59850 |
| AT1G60010 |
| AT1G60130 |
| AT1G60420 |
| AT1G60610 |
| AT1G60710 |
| AT1G60890 |
| AT1G60950 |
| AT1G61170 |
| AT1G61240 |
| AT1G61260 |
| AT1G61390 |
| AT1G61740 |
| AT1G61840 |
| AT1G61850 |
| AT1G61950 |
| AT1G62320 |
| AT1G62510 |
| AT1G62520 |
| AT1G62660 |
| AT1G62870 |
| AT1G62980 |
| AT1G62990 |
| AT1G63010 |
| AT1G63120 |
| AT1G63450 |
| AT1G63480 |
| AT1G63580 |
| AT1G63600 |
| AT1G63650 |
| AT1G63930 |
| AT1G64250 |
| AT1G64330 |
| AT1G64390 |
| AT1G64670 |
| AT1G64690 |
| AT1G64850 |
| AT1G65180 |
| AT1G65310 |
| AT1G65540 |
| AT1G65580 |
| AT1G65610 |
| AT1G66080 |
| AT1G66150 |
| AT1G66460 |
| AT1G66470 |
| AT1G66880 |
| AT1G67330 |
| AT1G67360 |
| AT1G67410 |
| AT1G67480 |
| AT1G67750 |
| AT1G67960 |
| AT1G68070 |
| AT1G68160 |
| AT1G68400 |
| AT1G68470 |
| AT1G68640 |
| AT1G69240 |
| AT1G69295 |
| AT1G69740 |
| AT1G69870 |
| AT1G69930 |
| AT1G70290 |
| AT1G70370 |
| AT1G70450 |
| AT1G70460 |
| AT1G70470 |
| AT1G70660 |
| AT1G70770 |
| AT1G70940 |
| AT1G70990 |
| AT1G71030 |
| AT1G71090 |
| AT1G71530 |
| AT1G71696 |
| AT1G71710 |
| AT1G71780 |
| AT1G71880 |
| AT1G72125 |
| AT1G72140 |
| AT1G72200 |
| AT1G72220 |
| AT1G72280 |
| AT1G72870 |
| AT1G72920 |
| AT1G72970 |
| AT1G73170 |
| AT1G73200 |
| AT1G73250 |
| AT1G73360 |
| AT1G73430 |
| AT1G73590 |
| AT1G73680 |
| AT1G73740 |
| AT1G73860 |
| AT1G74100 |
| AT1G74210 |
| AT1G74340 |
| AT1G74540 |
| AT1G74640 |
| AT1G74740 |
| AT1G74780 |
| AT1G75160 |
| AT1G75220 |
| AT1G75840 |
| AT1G75850 |
| AT1G76090 |
| AT1G76260 |
| AT1G76270 |
| AT1G76470 |
| AT1G76550 |
| AT1G76590 |
| AT1G76850 |
| AT1G77280 |
| AT1G77810 |
| AT1G77920 |
| AT1G78050 |
| AT1G78210 |
| AT1G78280 |
| AT1G78900 |
| AT1G79320 |
| AT1G79340 |
| AT1G79450 |
| AT1G79790 |
| AT1G79840 |
| AT1G79860 |
| AT1G79970 |
| AT1G80280 |
| AT1G80350 |
| AT1G80690 |
| AT1G80850 |
| AT1G80900 |
| AT1G80910 |
| AT1G80960 |
| AT2G01090 |
| AT2G01150 |
| AT2G01540 |
| AT2G01670 |
| AT2G01820 |
| AT2G02300 |
| AT2G02360 |
| AT2G02480 |
| AT2G02510 |
| AT2G02620 |
| AT2G02630 |
| AT2G02690 |
| AT2G02810 |
| AT2G03240 |
| AT2G03340 |
| AT2G03720 |
| AT2G03760 |
| AT2G03850 |
| AT2G04050 |
| AT2G04480 |
| AT2G04550 |
| AT2G04650 |
| AT2G04680 |
| AT2G04800 |
| AT2G05160 |
| AT2G05210 |
| AT2G05520 |
| AT2G06850 |
| AT2G06925 |
| AT2G07667 |
| AT2G07671 |
| AT2G15090 |
| AT2G15320 |
| AT2G15440 |
| AT2G15960 |
| AT2G16230 |
| AT2G16660 |
| AT2G16870 |
| AT2G16920 |
| AT2G17080 |
| AT2G17130 |
| AT2G17430 |
| AT2G17440 |
| AT2G17590 |
| AT2G17650 |
| AT2G17720 |
| AT2G17790 |
| AT2G17840 |
| AT2G17890 |
| AT2G18450 |
| AT2G18470 |
| AT2G18620 |
| AT2G18690 |
| AT2G18720 |
| AT2G19060 |
| AT2G19580 |
| AT2G19590 |
| AT2G20030 |
| AT2G20440 |
| AT2G20520 |
| AT2G20610 |
| AT2G20625 |
| AT2G20650 |
| AT2G20680 |
| AT2G20720 |
| AT2G20750 |
| AT2G20790 |
| AT2G20800 |
| AT2G21045 |
| AT2G21060 |
| AT2G21130 |
| AT2G21140 |
| AT2G21180 |
| AT2G21510 |
| AT2G21520 |
| AT2G21640 |
| AT2G21850 |
| AT2G21860 |
| AT2G22290 |
| AT2G22330 |
| AT2G22560 |
| AT2G22670 |
| AT2G22690 |
| AT2G22740 |
| AT2G22760 |
| AT2G22910 |
| AT2G22970 |
| AT2G23270 |
| AT2G23960 |
| AT2G23980 |
| AT2G24070 |
| AT2G24150 |
| AT2G24180 |
| AT2G24190 |
| AT2G24260 |
| AT2G24280 |
| AT2G24310 |
| AT2G24330 |
| AT2G24800 |
| AT2G25240 |
| AT2G25250 |
| AT2G25310 |
| AT2G25350 |
| AT2G25520 |
| AT2G25940 |
| AT2G25980 |
| AT2G26110 |
| AT2G26410 |
| AT2G26470 |
| AT2G26480 |
| AT2G26540 |
| AT2G26640 |
| AT2G26690 |
| AT2G26730 |
| AT2G26820 |
| AT2G26870 |
| AT2G26890 |
| AT2G27000 |
| AT2G27080 |
| AT2G27440 |
| AT2G27480 |
| AT2G27790 |
| AT2G28080 |
| AT2G28440 |
| AT2G28690 |
| AT2G28710 |
| AT2G28720 |
| AT2G28950 |
| AT2G29460 |
| AT2G29620 |
| AT2G29670 |
| AT2G29740 |
| AT2G30140 |
| AT2G31250 |
| AT2G31350 |
| AT2G31400 |
| AT2G31490 |
| AT2G32235 |
| AT2G32240 |
| AT2G32280 |
| AT2G32380 |
| AT2G32810 |
| AT2G32850 |
| AT2G32910 |
| AT2G32950 |
| AT2G32980 |
| AT2G32990 |
| AT2G33220 |
| AT2G33255 |
| AT2G33390 |
| AT2G33460 |
| AT2G33850 |
| AT2G34070 |
| AT2G34140 |
| AT2G34660 |
| AT2G34690 |
| AT2G34730 |
| AT2G34810 |
| AT2G34850 |
| AT2G34910 |
| AT2G34940 |
| AT2G35060 |
| AT2G35585 |
| AT2G35610 |
| AT2G35670 |
| AT2G35890 |
| AT2G36130 |
| AT2G36470 |
| AT2G36490 |
| AT2G36810 |
| AT2G36900 |
| AT2G37260 |
| AT2G37290 |
| AT2G37390 |
| AT2G37440 |
| AT2G37670 |
| AT2G37760 |
| AT2G37820 |
| AT2G37840 |
| AT2G38090 |
| AT2G38100 |
| AT2G38320 |
| AT2G38490 |
| AT2G38500 |
| AT2G38710 |
| AT2G38750 |
| AT2G38760 |
| AT2G38780 |
| AT2G38860 |
| AT2G38950 |
| AT2G38960 |
| AT2G39010 |
| AT2G39050 |
| AT2G39100 |
| AT2G39360 |
| AT2G39560 |
| AT2G39690 |
| AT2G39870 |
| AT2G39900 |
| AT2G39950 |
| AT2G40010 |
| AT2G40070 |
| AT2G40400 |
| AT2G40475 |
| AT2G41090 |
| AT2G41380 |
| AT2G41445 |
| AT2G41490 |
| AT2G41890 |
| AT2G41970 |
| AT2G42060 |
| AT2G42160 |
| AT2G42190 |
| AT2G42280 |
| AT2G42400 |
| AT2G42490 |
| AT2G42600 |
| AT2G43040 |
| AT2G43350 |
| AT2G43640 |
| AT2G43850 |
| AT2G43890 |
| AT2G43900 |
| AT2G44110 |
| AT2G44200 |
| AT2G44210 |
| AT2G44230 |
| AT2G44420 |
| AT2G45180 |
| AT2G45220 |
| AT2G45320 |
| AT2G45750 |
| AT2G45830 |
| AT2G45870 |
| AT2G45890 |
| AT2G46030 |
| AT2G46170 |
| AT2G46180 |
| AT2G46310 |
| AT2G46370 |
| AT2G46410 |
| AT2G46490 |
| AT2G46860 |
| AT2G47160 |
| AT2G47190 |
| AT2G47240 |
| AT2G47540 |
| AT2G47800 |
| AT2G47830 |
| AT2G48080 |
| AT3G01070 |
| AT3G01170 |
| AT3G01310 |
| AT3G01720 |
| AT3G01730 |
| AT3G01850 |
| AT3G01930 |
| AT3G01980 |
| AT3G02240 |
| AT3G02250 |
| AT3G02570 |
| AT3G02590 |
| AT3G02875 |
| AT3G02885 |
| AT3G02990 |
| AT3G03290 |
| AT3G03520 |
| AT3G03790 |
| AT3G04090 |
| AT3G04360 |
| AT3G04530 |
| AT3G04630 |
| AT3G04870 |
| AT3G04940 |
| AT3G05010 |
| AT3G05170 |
| AT3G05230 |
| AT3G05710 |
| AT3G05800 |
| AT3G05820 |
| AT3G05990 |
| AT3G06035 |
| AT3G06070 |
| AT3G06150 |
| AT3G06300 |
| AT3G06450 |
| AT3G06470 |
| AT3G06870 |
| AT3G07070 |
| AT3G07190 |
| AT3G07360 |
| AT3G07490 |
| AT3G07760 |
| AT3G07880 |
| AT3G07900 |
| AT3G07960 |
| AT3G07990 |
| AT3G08730 |
| AT3G08770 |
| AT3G09240 |
| AT3G09300 |
| AT3G09540 |
| AT3G09710 |
| AT3G09810 |
| AT3G09970 |
| AT3G10190 |
| AT3G10200 |
| AT3G10330 |
| AT3G10520 |
| AT3G10660 |
| AT3G10710 |
| AT3G10920 |
| AT3G11210 |
| AT3G11330 |
| AT3G11580 |
| AT3G11900 |
| AT3G12500 |
| AT3G12510 |
| AT3G12540 |
| AT3G13200 |
| AT3G13360 |
| AT3G13650 |
| AT3G13772 |
| AT3G13782 |
| AT3G14270 |
| AT3G14280 |
| AT3G14410 |
| AT3G14660 |
| AT3G14850 |
| AT3G14960 |
| AT3G15020 |
| AT3G15360 |
| AT3G15370 |
| AT3G15410 |
| AT3G15530 |
| AT3G15710 |
| AT3G15730 |
| AT3G15760 |
| AT3G15950 |
| AT3G15980 |
| AT3G15990 |
| AT3G16170 |
| AT3G16180 |
| AT3G16320 |
| AT3G16370 |
| AT3G16460 |
| AT3G16490 |
| AT3G16690 |
| AT3G16750 |
| AT3G16800 |
| AT3G17120 |
| AT3G17600 |
| AT3G17780 |
| AT3G17800 |
| AT3G17900 |
| AT3G18040 |
| AT3G18270 |
| AT3G18440 |
| AT3G18780 |
| AT3G19100 |
| AT3G19210 |
| AT3G19290 |
| AT3G19320 |
| AT3G19370 |
| AT3G19390 |
| AT3G19450 |
| AT3G19553 |
| AT3G19770 |
| AT3G20370 |
| AT3G20410 |
| AT3G20460 |
| AT3G20560 |
| AT3G20720 |
| AT3G20830 |
| AT3G20860 |
| AT3G20870 |
| AT3G20960 |
| AT3G21070 |
| AT3G21180 |
| AT3G21310 |
| AT3G21340 |
| AT3G21420 |
| AT3G21600 |
| AT3G21750 |
| AT3G22120 |
| AT3G22570 |
| AT3G22968 |
| AT3G22970 |
| AT3G23190 |
| AT3G23540 |
| AT3G23610 |
| AT3G23800 |
| AT3G23870 |
| AT3G24530 |
| AT3G24630 |
| AT3G24670 |
| AT3G24760 |
| AT3G24810 |
| AT3G24820 |
| AT3G25800 |
| AT3G26744 |
| AT3G26760 |
| AT3G26770 |
| AT3G27170 |
| AT3G27270 |
| AT3G27300 |
| AT3G27470 |
| AT3G27650 |
| AT3G27920 |
| AT3G28050 |
| AT3G28360 |
| AT3G28910 |
| AT3G29400 |
| AT3G29410 |
| AT3G29770 |
| AT3G43670 |
| AT3G43960 |
| AT3G44070 |
| AT3G44340 |
| AT3G44735 |
| AT3G44940 |
| AT3G45090 |
| AT3G45230 |
| AT3G45620 |
| AT3G46270 |
| AT3G46540 |
| AT3G46590 |
| AT3G46760 |
| AT3G47210 |
| AT3G47340 |
| AT3G47480 |
| AT3G47740 |
| AT3G47810 |
| AT3G48090 |
| AT3G48350 |
| AT3G48460 |
| AT3G48760 |
| AT3G48850 |
| AT3G49220 |
| AT3G49490 |
| AT3G49590 |
| AT3G49650 |
| AT3G49960 |
| AT3G50110 |
| AT3G50120 |
| AT3G50130 |
| AT3G50140 |
| AT3G50150 |
| AT3G50700 |
| AT3G50870 |
| AT3G51160 |
| AT3G51470 |
| AT3G51540 |
| AT3G51960 |
| AT3G51990 |
| AT3G52190 |
| AT3G52200 |
| AT3G52460 |
| AT3G52540 |
| AT3G52760 |
| AT3G52870 |
| AT3G53100 |
| AT3G53150 |
| AT3G53240 |
| AT3G53260 |
| AT3G53350 |
| AT3G53370 |
| AT3G53410 |
| AT3G53540 |
| AT3G54040 |
| AT3G54110 |
| AT3G54140 |
| AT3G54400 |
| AT3G54480 |
| AT3G54500 |
| AT3G54580 |
| AT3G54590 |
| AT3G54870 |
| AT3G54960 |
| AT3G54990 |
| AT3G55550 |
| AT3G55700 |
| AT3G55710 |
| AT3G56000 |
| AT3G56500 |
| AT3G56840 |
| AT3G56930 |
| AT3G56940 |
| AT3G57020 |
| AT3G57040 |
| AT3G57480 |
| AT3G57540 |
| AT3G57800 |
| AT3G58000 |
| AT3G58570 |
| AT3G58710 |
| AT3G58790 |
| AT3G58860 |
| AT3G59290 |
| AT3G59830 |
| AT3G60260 |
| AT3G60280 |
| AT3G60330 |
| AT3G60380 |
| AT3G60440 |
| AT3G60540 |
| AT3G60900 |
| AT3G61300 |
| AT3G61570 |
| AT3G61630 |
| AT3G61760 |
| AT3G61820 |
| AT3G61990 |
| AT3G62010 |
| AT3G62370 |
| AT3G62680 |
| AT3G62780 |
| AT3G62800 |
| AT3G63150 |
| AT3G63330 |
| AT4G00240 |
| AT4G00335 |
| AT4G00440 |
| AT4G00460 |
| AT4G00480 |
| AT4G00650 |
| AT4G00680 |
| AT4G00700 |
| AT4G01050 |
| AT4G01110 |
| AT4G01470 |
| AT4G01480 |
| AT4G01660 |
| AT4G01890 |
| AT4G02270 |
| AT4G02280 |
| AT4G02390 |
| AT4G02680 |
| AT4G03140 |
| AT4G03210 |
| AT4G03330 |
| AT4G03480 |
| AT4G03550 |
| AT4G03560 |
| AT4G04700 |
| AT4G04750 |
| AT4G04760 |
| AT4G04900 |
| AT4G05330 |
| AT4G07960 |
| AT4G08250 |
| AT4G08280 |
| AT4G08410 |
| AT4G08450 |
| AT4G08620 |
| AT4G08685 |
| AT4G08950 |
| AT4G09160 |
| AT4G09500 |
| AT4G09760 |
| AT4G09990 |
| AT4G10370 |
| AT4G10770 |
| AT4G10840 |
| AT4G10925 |
| AT4G10955 |
| AT4G10960 |
| AT4G11080 |
| AT4G11090 |
| AT4G11170 |
| AT4G11230 |
| AT4G11260 |
| AT4G11270 |
| AT4G11410 |
| AT4G11550 |
| AT4G11570 |
| AT4G11590 |
| AT4G11860 |
| AT4G12010 |
| AT4G12020 |
| AT4G12030 |
| AT4G12120 |
| AT4G12300 |
| AT4G12330 |
| AT4G12470 |
| AT4G12990 |
| AT4G13020 |
| AT4G13120 |
| AT4G13270 |
| AT4G13320 |
| AT4G13390 |
| AT4G13420 |
| AT4G13440 |
| AT4G13660 |
| AT4G13670 |
| AT4G13690 |
| AT4G13830 |
| AT4G13860 |
| AT4G14070 |
| AT4G14280 |
| AT4G14360 |
| AT4G14390 |
| AT4G14480 |
| AT4G14490 |
| AT4G14500 |
| AT4G14610 |
| AT4G14760 |
| AT4G14965 |
| AT4G14980 |
| AT4G15093 |
| AT4G15230 |
| AT4G15290 |
| AT4G15390 |
| AT4G15475 |
| AT4G15550 |
| AT4G15740 |
| AT4G15780 |
| AT4G15910 |
| AT4G15920 |
| AT4G16120 |
| AT4G16130 |
| AT4G16190 |
| AT4G16330 |
| AT4G16350 |
| AT4G16400 |
| AT4G16420 |
| AT4G16444 |
| AT4G16450 |
| AT4G16600 |
| AT4G16670 |
| AT4G16710 |
| AT4G16760 |
| AT4G16770 |
| AT4G16845 |
| AT4G16850 |
| AT4G16890 |
| AT4G16920 |
| AT4G17080 |
| AT4G17100 |
| AT4G17215 |
| AT4G17483 |
| AT4G17570 |
| AT4G17970 |
| AT4G17990 |
| AT4G18300 |
| AT4G18460 |
| AT4G18580 |
| AT4G18640 |
| AT4G18700 |
| AT4G18980 |
| AT4G19160 |
| AT4G19490 |
| AT4G19500 |
| AT4G19660 |
| AT4G19680 |
| AT4G19830 |
| AT4G19865 |
| AT4G19960 |
| AT4G20110 |
| AT4G20170 |
| AT4G20330 |
| AT4G20410 |
| AT4G20450 |
| AT4G20480 |
| AT4G20760 |
| AT4G21120 |
| AT4G21230 |
| AT4G21480 |
| AT4G21700 |
| AT4G21720 |
| AT4G21750 |
| AT4G21810 |
| AT4G21850 |
| AT4G22070 |
| AT4G22280 |
| AT4G22460 |
| AT4G22640 |
| AT4G22770 |
| AT4G22780 |
| AT4G22830 |
| AT4G22910 |
| AT4G22930 |
| AT4G22980 |
| AT4G23885 |
| AT4G23900 |
| AT4G24030 |
| AT4G24400 |
| AT4G24580 |
| AT4G24780 |
| AT4G24920 |
| AT4G24960 |
| AT4G25050 |
| AT4G25070 |
| AT4G25080 |
| AT4G25090 |
| AT4G25110 |
| AT4G25160 |
| AT4G25170 |
| AT4G25220 |
| AT4G25420 |
| AT4G25520 |
| AT4G25570 |
| AT4G25620 |
| AT4G25790 |
| AT4G25820 |
| AT4G25940 |
| AT4G26010 |
| AT4G26100 |
| AT4G26320 |
| AT4G26330 |
| AT4G26570 |
| AT4G26770 |
| AT4G26830 |
| AT4G26850 |
| AT4G26910 |
| AT4G27180 |
| AT4G27270 |
| AT4G27290 |
| AT4G27320 |
| AT4G27435 |
| AT4G27480 |
| AT4G27710 |
| AT4G27780 |
| AT4G27830 |
| AT4G27850 |
| AT4G28030 |
| AT4G28170 |
| AT4G28260 |
| AT4G28390 |
| AT4G28410 |
| AT4G28540 |
| AT4G28850 |
| AT4G29020 |
| AT4G29180 |
| AT4G29210 |
| AT4G29220 |
| AT4G29550 |
| AT4G29800 |
| AT4G29900 |
| AT4G30160 |
| AT4G30320 |
| AT4G30460 |
| AT4G30530 |
| AT4G30560 |
| AT4G30610 |
| AT4G30640 |
| AT4G30830 |
| AT4G30996 |
| AT4G31020 |
| AT4G31250 |
| AT4G31450 |
| AT4G31500 |
| AT4G31740 |
| AT4G31760 |
| AT4G31800 |
| AT4G32000 |
| AT4G32160 |
| AT4G32290 |
| AT4G32300 |
| AT4G32460 |
| AT4G32530 |
| AT4G32920 |
| AT4G33220 |
| AT4G33360 |
| AT4G33530 |
| AT4G33730 |
| AT4G33740 |
| AT4G33790 |
| AT4G33810 |
| AT4G34050 |
| AT4G34540 |
| AT4G34580 |
| AT4G34588 |
| AT4G34590 |
| AT4G34710 |
| AT4G34760 |
| AT4G34880 |
| AT4G34980 |
| AT4G34990 |
| AT4G35230 |
| AT4G35380 |
| AT4G35500 |
| AT4G35560 |
| AT4G36010 |
| AT4G36160 |
| AT4G36430 |
| AT4G36630 |
| AT4G37270 |
| AT4G37300 |
| AT4G37370 |
| AT4G37400 |
| AT4G37550 |
| AT4G37640 |
| AT4G37870 |
| AT4G37890 |
| AT4G37950 |
| AT4G38070 |
| AT4G38090 |
| AT4G38200 |
| AT4G38390 |
| AT4G38520 |
| AT4G38580 |
| AT4G38640 |
| AT4G38950 |
| AT4G38960 |
| AT4G38980 |
| AT4G39140 |
| AT4G39160 |
| AT4G39260 |
| AT4G39540 |
| AT4G39780 |
| AT4G39890 |
| AT4G40010 |
| AT4G40060 |
| AT4G40090 |
| AT5G01060 |
| AT5G01200 |
| AT5G01280 |
| AT5G01340 |
| AT5G01410 |
| AT5G01610 |
| AT5G01730 |
| AT5G01850 |
| AT5G02100 |
| AT5G02170 |
| AT5G02240 |
| AT5G02270 |
| AT5G02350 |
| AT5G02430 |
| AT5G02540 |
| AT5G02630 |
| AT5G03080 |
| AT5G03090 |
| AT5G03160 |
| AT5G03640 |
| AT5G04020 |
| AT5G04160 |
| AT5G04720 |
| AT5G04920 |
| AT5G04960 |
| AT5G05180 |
| AT5G05270 |
| AT5G05370 |
| AT5G05400 |
| AT5G05500 |
| AT5G05570 |
| AT5G05760 |
| AT5G05840 |
| AT5G05860 |
| AT5G06260 |
| AT5G06630 |
| AT5G06640 |
| AT5G06700 |
| AT5G06800 |
| AT5G06820 |
| AT5G06830 |
| AT5G06839 |
| AT5G07080 |
| AT5G07150 |
| AT5G07360 |
| AT5G07450 |
| AT5G07460 |
| AT5G07570 |
| AT5G07770 |
| AT5G07780 |
| AT5G07990 |
| AT5G08080 |
| AT5G08260 |
| AT5G09430 |
| AT5G09870 |
| AT5G10170 |
| AT5G10260 |
| AT5G10520 |
| AT5G10650 |
| AT5G10820 |
| AT5G10830 |
| AT5G10950 |
| AT5G11020 |
| AT5G11110 |
| AT5G11230 |
| AT5G12010 |
| AT5G12040 |
| AT5G12050 |
| AT5G13150 |
| AT5G13330 |
| AT5G13460 |
| AT5G13500 |
| AT5G13840 |
| AT5G13990 |
| AT5G14120 |
| AT5G14330 |
| AT5G14470 |
| AT5G14840 |
| AT5G14920 |
| AT5G15070 |
| AT5G15180 |
| AT5G15350 |
| AT5G15600 |
| AT5G15725 |
| AT5G15740 |
| AT5G15890 |
| AT5G15910 |
| AT5G15930 |
| AT5G15948 |
| AT5G15950 |
| AT5G16030 |
| AT5G16120 |
| AT5G16540 |
| AT5G16730 |
| AT5G16900 |
| AT5G16910 |
| AT5G17040 |
| AT5G17430 |
| AT5G18170 |
| AT5G18290 |
| AT5G18680 |
| AT5G18780 |
| AT5G18910 |
| AT5G19240 |
| AT5G19560 |
| AT5G19800 |
| AT5G19850 |
| AT5G19875 |
| AT5G20150 |
| AT5G20660 |
| AT5G20990 |
| AT5G21120 |
| AT5G21130 |
| AT5G21170 |
| AT5G22300 |
| AT5G22400 |
| AT5G22410 |
| AT5G23030 |
| AT5G23850 |
| AT5G24140 |
| AT5G24290 |
| AT5G24310 |
| AT5G24870 |
| AT5G24880 |
| AT5G25360 |
| AT5G25460 |
| AT5G25810 |
| AT5G25820 |
| AT5G25880 |
| AT5G25900 |
| AT5G26080 |
| AT5G26340 |
| AT5G27150 |
| AT5G27550 |
| AT5G27560 |
| AT5G27600 |
| AT5G27670 |
| AT5G27920 |
| AT5G27950 |
| AT5G28150 |
| AT5G28220 |
| AT5G28520 |
| AT5G28640 |
| AT5G34930 |
| AT5G35190 |
| AT5G35410 |
| AT5G35460 |
| AT5G35670 |
| AT5G35700 |
| AT5G36260 |
| AT5G36270 |
| AT5G37490 |
| AT5G38380 |
| AT5G38790 |
| AT5G38820 |
| AT5G38850 |
| AT5G39570 |
| AT5G40210 |
| AT5G40270 |
| AT5G40330 |
| AT5G40450 |
| AT5G40460 |
| AT5G40510 |
| AT5G40860 |
| AT5G40960 |
| AT5G41540 |
| AT5G41700 |
| AT5G41710 |
| AT5G41730 |
| AT5G42090 |
| AT5G42100 |
| AT5G42790 |
| AT5G42880 |
| AT5G43170 |
| AT5G43230 |
| AT5G43700 |
| AT5G43730 |
| AT5G43750 |
| AT5G43860 |
| AT5G44150 |
| AT5G44210 |
| AT5G44470 |
| AT5G44480 |
| AT5G45260 |
| AT5G45370 |
| AT5G45650 |
| AT5G45800 |
| AT5G46040 |
| AT5G46150 |
| AT5G46230 |
| AT5G46250 |
| AT5G46340 |
| AT5G46660 |
| AT5G46670 |
| AT5G47060 |
| AT5G47260 |
| AT5G47530 |
| AT5G47550 |
| AT5G47600 |
| AT5G47810 |
| AT5G47990 |
| AT5G48010 |
| AT5G48380 |
| AT5G48480 |
| AT5G49080 |
| AT5G49270 |
| AT5G49330 |
| AT5G49680 |
| AT5G49760 |
| AT5G49800 |
| AT5G49870 |
| AT5G50010 |
| AT5G50011 |
| AT5G50012 |
| AT5G50210 |
| AT5G50260 |
| AT5G50330 |
| AT5G50350 |
| AT5G50360 |
| AT5G51050 |
| AT5G51180 |
| AT5G51260 |
| AT5G51270 |
| AT5G51550 |
| AT5G51570 |
| AT5G51740 |
| AT5G52030 |
| AT5G52670 |
| AT5G52810 |
| AT5G53050 |
| AT5G53100 |
| AT5G53200 |
| AT5G53420 |
| AT5G53760 |
| AT5G53990 |
| AT5G54130 |
| AT5G54540 |
| AT5G54650 |
| AT5G54780 |
| AT5G54840 |
| AT5G54860 |
| AT5G55060 |
| AT5G55100 |
| AT5G55170 |
| AT5G55350 |
| AT5G55380 |
| AT5G55400 |
| AT5G55780 |
| AT5G55950 |
| AT5G56160 |
| AT5G56260 |
| AT5G56610 |
| AT5G56950 |
| AT5G57040 |
| AT5G57350 |
| AT5G57420 |
| AT5G57530 |
| AT5G57540 |
| AT5G57610 |
| AT5G57830 |
| AT5G57840 |
| AT5G58010 |
| AT5G58160 |
| AT5G58340 |
| AT5G58600 |
| AT5G58630 |
| AT5G59220 |
| AT5G59530 |
| AT5G59540 |
| AT5G59700 |
| AT5G59800 |
| AT5G60100 |
| AT5G60120 |
| AT5G60548 |
| AT5G60550 |
| AT5G60690 |
| AT5G61228 |
| AT5G61230 |
| AT5G61260 |
| AT5G61350 |
| AT5G61360 |
| AT5G61450 |
| AT5G61550 |
| AT5G61650 |
| AT5G61980 |
| AT5G62165 |
| AT5G62310 |
| AT5G62480 |
| AT5G62540 |
| AT5G62880 |
| AT5G62930 |
| AT5G63190 |
| AT5G63260 |
| AT5G63450 |
| AT5G63490 |
| AT5G63590 |
| AT5G63670 |
| AT5G63910 |
| AT5G63980 |
| AT5G63990 |
| AT5G64330 |
| AT5G64630 |
| AT5G64640 |
| AT5G64900 |
| AT5G65020 |
| AT5G65090 |
| AT5G65100 |
| AT5G65160 |
| AT5G65210 |
| AT5G65290 |
| AT5G65490 |
| AT5G65910 |
| AT5G65930 |
| AT5G65990 |
| AT5G66590 |
| AT5G66730 |
| AT5G66920 |
| AT5G66985 |
| AT5G67160 |
| AT5G67290 |
| AT5G67370 |
| AT5G67400 |
| AT5G67460 |
| AT5G67480 |
| AT5G67520 |
| ATMG00040 |
| ATMG01080 |
