# Supplementary material for: A Gene Regulatory Network for Root Epidermis Cell Differentiation in Arabidopsis
Source: PLoS Genet. 2012 Jan 12;8(1):e1002446. doi: 10.1371/journal.pgen.1002446 (PMC3257299; doi:10.1371/journal.pgen.1002446)
Supplement: Table S3 — List of significant Gene Ontology classes among 1,582 genes differentially expressed in the hairy versus hairless mutant lines. (DOCX) [file pgen.1002446.s011.docx]

**Table S3.** List of Significant Gene Ontology Classes among 1582 Genes Differentially Expressed in Hairy vs. Hairless Mutant Lines.

| **p-value** | **Gene Ontology (GO)** | **Category** | **# genes in GO** | **# genes from 1582 list** |  |
| --- | --- | --- | --- | --- | --- |
| 4.50E-05 | 0005783 endoplasmic reticulum | comp | 312 | 36 |  |
| 5.00E-05 | 0005886 plasma membrane | comp | 1895 | 144 |  |
| 0.0014 | 0016020 membrane | comp | 1676 | 121 |  |
| 1.50E-03 | 0009506 plasmodesma | comp | 6 | 4 |  |
| 0.0025 | 0009505 plant-type cell wall | comp | 263 | 27 |  |
| 5.00E-03 | 0005773 vacuole | comp | 522 | 44 |  |
| 6.30E-03 | 0005774 vacuolar membrane | comp | 42 | 8 |  |
| 0.0066 | 0016021 integral to membrane | comp | 325 | 30 |  |
| 7.40E-03 | 0009986 cell surface | comp | 9 | 4 |  |
| 0.0126 | 0005576 extracellular region | comp | 94 | 12 |  |
| 0.0142 | 0005737 cytoplasm | comp | 495 | 40 |  |
| 0.0197 | 0031225 anchored to membrane | comp | 237 | 22 |  |
| 3.87E-02 | 0017119 Golgi transport complex | comp | 7 | 3 |  |
| 3.20E-07 | 0016301 kinase activity | func | 912 | 88 |  |
| 3.60E-07 | 0005509 calcium ion binding | func | 177 | 29 |  |
| 1.60E-06 | 0005199 structural constituent of cell wall | func | 43 | 13 |  |
| 5.40E-05 | 0005089 Rho guanyl-nucleotide exchange factor activity | func | 15 | 7 |  |
| 3.50E-04 | 0016757 transferase activity, transferring glycosyl groups | func | 333 | 35 |  |
| 4.10E-04 | 0005516 calmodulin binding | func | 171 | 22 |  |
| 5.20E-04 | 0003779 actin binding | func | 54 | 11 |  |
| 1.10E-03 | 0016491 oxidoreductase activity | func | 275 | 29 |  |
| 1.80E-03 | 0004683 calmodulin-dependent protein kinase activity | func | 34 | 8 |  |
| 2.90E-03 | 0050660 FAD binding | func | 28 | 7 |  |
| 0.003 | 0016787 hydrolase activity | func | 174 | 20 |  |
| 0.0036 | 0005215 transporter activity | func | 256 | 26 |  |
| 4.50E-03 | 0017111 nucleoside-triphosphatase activity | func | 82 | 12 |  |
| 0.0055 | 0016798 hydrolase activity, acting on glycosyl bonds | func | 41 | 8 |  |
| 5.50E-03 | 0030246 carbohydrate binding | func | 84 | 12 |  |
| 5.70E-03 | 0016308 1-phosphatidylinositol-4-phosphate 5-kinase activity | func | 15 | 5 |  |
| 8.60E-03 | 0004445 inositol-polyphosphate 5-phosphatase activity | func | 4 | 3 |  |
| 1.07E-02 | 0008138 protein tyrosine/serine/threonine phosphatase activity | func | 10 | 4 |  |
| 0.0108 | 0004601 peroxidase activity | func | 80 | 11 |  |
| 1.08E-02 | 0005515 protein binding | func | 1505 | 104 |  |
| 1.67E-02 | 0005484 SNAP receptor activity | func | 29 | 6 |  |
| 1.78E-02 | 0004437 inositol or phosphatidylinositol phosphatase activity | func | 15 | 4 |  |
| 1.94E-02 | 0008194 UDP-glycosyltransferase activity | func | 100 | 12 |  |
| 2.52E-02 | 0005506 iron ion binding | func | 32 | 6 |  |
| 0.0255 | 0004674 protein serine/threonine kinase activity | func | 186 | 18 |  |
| 4.19E-02 | 0015171 amino acid transmembrane transporter activity | func | 48 | 7 |  |
| 4.50E-02 | 0030570 pectate lyase activity | func | 26 | 5 |  |
| 4.54E-02 | 0005388 calcium-transporting ATPase activity | func | 16 | 4 |  |
| 4.81E-02 | 0000166 nucleotide binding | func | 291 | 24 |  |
| 0.0498 | 0004364 gluthatione transferase activity | func | 50 | 7 |  |
| 1.80E-09 | 0006468 protein amino acid phosphorylation | proc | 906 | 95 |  |
| 1.10E-05 | 0009664 plant-type cell wall organization | proc | 29 | 10 |  |
| 7.00E-05 | 0008152 metabolic process | proc | 656 | 61 |  |
| 4.00E-04 | 0006499 N-terminal protein myristoylation | proc | 430 | 42 |  |
| 9.30E-04 | 0006073 cellular glucan metabolic process | proc | 23 | 7 |  |
| 0.0014 | 0048765 root hair cell differentiation | proc | 11 | 5 |  |
| 0.003 | 0003777 microtubule motor activity | proc | 67 | 11 |  |
| 3.50E-03 | 0010091 trichome branching | proc | 29 | 7 |  |
| 0.0051 | 0006944 membrane fusion | proc | 31 | 7 |  |
| 0.006 | 0009813 flavonoid biosynthetic process | proc | 32 | 7 |  |
| 8.10E-03 | 0006886 intracellular protein transport | proc | 138 | 16 |  |
| 1.02E-02 | 0006810 transport | proc | 380 | 33 |  |
| 1.47E-02 | 0006623 protein targeting to vacuole | proc | 19 | 5 |  |
| 1.79E-02 | 0009630 gravitropism | proc | 20 | 5 |  |
| 0.0268 | 0009957 epidermal cell fate specification | proc | 6 | 3 |  |
| 0.0269 | 0007242 intracellular signalling cascade | proc | 92 | 11 |  |
| 3.09E-02 | 0048767 root hair elongation | proc | 14 | 4 |  |
| 3.16E-02 | 0008643 carbohydrate transport | proc | 45 | 7 |  |
| 0.0361 | 0009828 plant-type cell wall loosening | proc | 35 | 6 |  |
| 3.71E-02 | 0009753 response to jasmonic acid stimulus | proc | 124 | 13 |  |
| 0.0383 | 0009407 toxin catabolic process | proc | 47 | 7 |  |
| 4.49E-02 | 0007165 signal transduction | proc | 304 | 25 |  |
| 0.0469 | 0009826 unidimensional cell growth | proc | 101 | 11 |  |
|  |  |  |  |  |  |
