# Supplementary material for: A Gene Regulatory Network for Root Epidermis Cell Differentiation in Arabidopsis
Source: PLoS Genet. 2012 Jan 12;8(1):e1002446. doi: 10.1371/journal.pgen.1002446 (PMC3257299; doi:10.1371/journal.pgen.1002446)
Supplement: Table S4 — List of 208 core root epidermal genes. (DOCX) [file pgen.1002446.s012.docx]

**Table S4.** List of 208 Core Root Epidermal Genes.

| **Gene ID** | **Gene Name** | **Hair (H) or Non-Hair (N) Gene*** | **Description of Gene Product (from TAIR)** | **Gene Ontology (GO) Categories** | **Root Hair Regulatory Element (RHE) in Promoter? **** |
| --- | --- | --- | --- | --- | --- |
| AT1G01380 | **ETC1** (ENHANCER OF TRY AND CPC1) | **N** | Transcription Factor, involved in trichome and root hair patterning in Arabidopsis | GO:0003677: DNA binding GO:0003700: transcription factor activity GO:0045449: regulation of transcription GO:0006355: regulation of transcription, DNA-dependent | No |
| AT1G01750 | **ADF11** (ACTIN DEPOLY-MERIZING FACTOR 11) | **H** | Functions in actin binding | GO:0005622: intracellular GO:0003779: actin binding GO:0008150: biological process unknown GO:0005622: intracellular | No |
| AT1G03550 |  | **H** | Secretory carrier membrane protein (SCAMP) family protein | GO:0022857: transmembrane transporter activity GO:0015031: protein transport GO:0016021: integral to membrane | Yes |
| AT1G04700 |  | **H** | Protein kinase family protein | GO:0006468: protein amino acid phosphorylation GO:0004672: protein kinase activity GO:0004712: protein serine/threonine/tyrosine kinase activity GO:0005829: cytosol GO:0006468: protein amino acid phosphorylation | Yes |
| AT1G05630 | **5PTASE13,** AT5PTASE13, | **H** | Inositol polyphosphate 5-phosphatase with phosphatase activity toward only Ins(1,4,5)P3 | GO:0004437: inositol or phosphatidylinositol phosphatase activity GO:0005634: nucleus GO:0010087: phloem or xylem histogenesis GO:0009737: response to abscisic acid stimulus GO:0009637: response to blue light GO:0048364: root development GO:0009743: response to carbohydrate stimulus GO:0010252: auxin homeostasis GO:0009611: response to wounding GO:0010182 : sugar mediated signaling pathway GO:0007584: response to nutrient | Yes |
| AT1G06550 |  | **N** | Enoyl-CoA hydratase/isomerase family protein; 3-hydroxyisobutyryl-CoA hydrolase activity; involved in fatty acid beta-oxidation | GO:0003860: 3-hydroxyisobutyryl-CoA hydrolase activity GO:0005575: cellular component unknown GO:0008152: metabolic process GO:0006635: fatty acid beta-oxidation GO:0003824: catalytic activity | No |
| AT1G07795 |  | **H** | Unknown protein | GO:0008150: biological process unknown GO:0003674: molecular function unknown GO:0005575: cellular component unknown | No |
| AT1G09170 |  | **H** | Kinesin motor protein-related | GO:0003777: microtubule motor activity GO:0005524: ATP binding GO:0005875: microtubule associated complex GO:0007018: microtubule-based movement | No |
| AT1G10550 | **XTH33**, XET | **N** | Xyloglucan:xyloglucosyl transferase; membrane-localized protein, predicted to function during cell wall modification | GO:0004553: hydrolase activity, hydrolyzing O-glycosyl compounds GO:0005887: integral to plasma membrane GO:0009831: plant-type cell wall modification during multidimensional cell growth GO:0016762: xyloglucan:xyloglucosyl transferase activity GO:0016798: hydrolase activity, acting on glycosyl bonds | No |
| AT1G12040 | **LRX1** (Leucin-rich repeat/ Extensin1) | **H** | Chimeric leucine-rich repeat/extensin protein that regulates root hair morphogenesis and elongation | GO:0005199: structural constituent of cell wall GO:0009505: plant-type cell wall GO:0009826: unidimensional cell growth GO:0009927: histidine phosphotransfer kinase activity GO:0000904: cell morphogenesis involved in differentiation GO:0010054: trichoblast differentiation GO:0005515: protein binding | Yes |
| AT1G12560 | **EXP7,** ATEXPA7, (Expansins A7) | **H** | Member of Alpha-Expansin Gene Family, containing a conserved root hair-specific cis-element RHE, expressed specifically in root hair cell | GO:0005576: extracellular region GO:0009831: plant-type cell wall modification during multidimensional cell growth GO:0009826: unidimensional cell growth GO:0009828: plant-type cell wall loosening GO:0012505: endomembrane system | Yes |
| AT1G12950 |  | **H** | MATE efflux family protein; antiporter activity | GO:0015238: drug transporter activity GO:0015297: antiporter activity GO:0016020: membrane GO:0005215: transporter activity GO:0006855: multidrug transport | Yes |
| AT1G14685 | **BPC2**, BBR/BPC2, ATBPC2 (Basic Pentacysteine 2) | **H** | Transcription Factor | GO:0003700: transcription factor activity GO:0006355: regulation of transcription, DNA-dependent GO:0003677: DNA binding GO:0005634: nucleus | No |
| AT1G15040 |  | **H** | Glutamine amidotransferase-related | GO:0016787: hydrolase activity GO:0006541: glutamine metabolic process GO:0005575: cellular component unknown | Yes |
| AT1G15880 | **GOS11**, atgos11 (Golgi SNARE 11) | **H** | Golgi snare protein | GO:0016021: integral to membrane GO:0000149: SNARE binding GO:0006944: membrane fusion GO:0006891: intra-Golgi vesicle-mediated transport | No |
| AT1G16260 |  | **H** | Protein kinase family protein | GO:0006468: protein amino acid phosphorylation GO:0016021: integral to membrane GO:0016301: kinase activity GO:0012505: endomembrane system | No |
| AT1G16360 |  | **N** | LEM3 (ligand-effect modulator 3) family protein / CDC50 family protein | GO:0016020: membrane GO:0008150: biological process unknown | No |
| AT1G16440 |  | **H** | Kinase | GO:0016301: kinase activity GO:0005886: plasma membrane GO:0006468: protein amino acid phosphorylation | Yes |
| AT1G18250 | **ATLP-1** | **N** | Thaumatin-like protein | GO:0012505: endomembrane system GO:0051707:response to other organism | No |
| AT1G18940 |  | **H** | Nodulin family protein | GO:0012505: endomembrane system | No |
| AT1G22500 |  | **H** | Zinc finger (C3HC4-type RING finger) family protein | GO:0008270: zinc ion binding GO:0005515: protein binding GO:0012505: endomembrane system | No |
| AT1G22570 |  | **H** | Proton-dependent oligopeptide transport (POT) family protein | GO:0006857: oligopeptide transport GO:0005215: transporter activity GO:0016020: membrane | No |
| AT1G25450 | **KCS5**, CER60 (3-Ketoacyl-CoA Synthase5) | **N** | 3-ketoacyl-CoA synthase familyprotein, involved in the biosynthesis of VLCFA (very long chain fatty acids) | GO:0009409: response to cold GO:0009922: fatty acid elongase activity GO:0048868: pollen tube development GO:0000038: very-long-chain fatty acid metabolic process GO:0016020: membrane GO:0042335: cuticle development GO:0005783: endoplasmic reticulum GO:0009416: response to light stimulus | No |
| AT1G25460 |  | **N** | Oxidoreductase family protein; involved in flavonoid biosynthetic process | GO:0009813: flavonoid biosynthetic process GO:0050662: coenzyme binding GO:0003824: catalytic activity GO:0016491: oxidoreductase activity GO:0005575: cellular_component_unknown GO:0044237: cellular metabolic process GO:0005488: binding GO:0008152: metabolic process | No |
| AT1G26770 | **EXP10**, (Expansin A10) | **N** | Expansin, involved in the formation of nematode-induced syncytia in roots of Arabidopsis thaliana. | GO:0005199: structural constituent of cell wall GO:0009828: plant-type cell wall loosening GO:0009826: unidimensional cell growth GO:0006949: syncytium formation GO:0009831: plant-type cell wall modification during multidimensional cell growth GO:0009505: plant-type cell wall | No |
| AT1G27740 | **bHLH054** | **H** | Basic helix-loop-helix (bHLH) family protein | GO:0003677: DNA binding GO:0045449: regulation of transcription GO:0005634: nucleus GO:0003700: transcription factor activity | No |
| AT1G27950 | **LTPG1** (Glycosylphos-phatidylinositol - anchored lipid transfer protein) | **N** | Lipid transfer protein with a predicted GPI (glycosylphosphatidyl-inositol) -anchor domain | GO:0006869:  lipid transport GO:0005886:  plasma membrane GO:0031225: anchored to membrane GO:0046658:anchored to plasma membrane | No |
| AT1G29050 |  | **N** | Unknown protein | GO:0005886: plasma membrane GO:0003674: molecular function unknown GO:0006869: lipid transport GO:0031225: anchored to membrane GO:0046658: anchored to plasma membrane GO:0008150: biological process unknown GO:0012505: endomembrane system | No |
| AT1G30850 |  | **H** | Unknown protein | GO:0008150: biological process unknown GO:0005575: cellular component unknown GO:0003674: molecular function unknown | Yes |
| AT1G30870 |  | **H** | Cationic peroxidase, involved in response to oxidative stress | GO:0004601: peroxidase activity GO:0012505: endomembrane system GO:0009055: electron carrier activity GO:0020037: heme binding GO:0006979: response to oxidative stress | No |
| AT1G30990 |  | **H** | Major latex protein-related / MLP-related | GO:0009607: response to biotic stimulus GO:0006952: defense response GO:0005575: cellular component unknown GO:0003674: molecular function unknown | No |
| AT1G32870 | **ANAC13**, ANAC013 (NAC domain protein 13) | **N** | Transcription factor; involved in multicellular organismal development | GO:0003700: transcription factor activity GO:0005575: cellular component unknown GO:0010114: response to red light GO:0010224: response to UV-B GO:0007275: multicellular organismal development | No |
| AT1G33700 |  | **H** | Catalytic/ glucosylceramidase; glucosylceramidase activity, involved sphingolipid metabolic process | GO:0004348: glucosylceramidase activity GO:0003824: catalytic activity GO:0005773: vacuole GO:0006665: sphingolipid metabolic process GO:0006680: glucosylceramide catabolic process | No |
| AT1G34330 |  | **H** | Pseudogene, putative peroxidase |  | No |
| AT1G34510 |  | **H** | Putative Peroxidase; electron carrier activity, involved in response to oxidative stress | GO:0012505: endomembrane system GO:0004601: peroxidase activity GO:0006979: response to oxidative stress GO:0020037: heme binding GO:0009055: electron carrier activity | No |
| AT1G34760 | **GRF11**, GF14 OMICRON (General regulatory factor 11) | **H** | 14-3-3 protein, binds H+-ATPase in response to blue light | GO:0005515: protein binding GO:0051117: ATPase binding GO:0016597: amino acid binding GO:0005575: cellular component unknown GO:0045309: protein phosphorylated amino acid binding | No |
| AT1G35330 |  | **H** | Zinc finger (C3HC4-type RING finger) family protein | GO:0005515: protein binding GO:0012505: endomembrane system GO:0008270: zinc ion binding | No |
| AT1G35670 | **CPK11,** ATCDPK2, (Calcium-dependent protein kinase 2) | **H** | Ca(2+)-dependent, calmodulin-dependent protein kinase, positive regulator of ABA signaling | GO:0006468: protein amino acid phosphorylation GO:0005886: plasma membrane GO:0006468: protein amino acid phosphorylation GO:0016301: kinase activity GO:0009789: positive regulation of abscisic acid mediated signaling pathway GO:0004683: calmodulin-dependent protein kinase activity | No |
| AT1G48640 |  | **H** | Lysine and histidine specific transporter, putative | GO:0015171: amino acid transmembrane transporter activity GO:0006865: amino acid transport GO:0016020: membrane | No |
| AT1G48930 | **GH9C1** (Glycosyl hydrolase 9C1) | **H** | Glycosyl hydrolase 9C1; involved in carbohydrate metabolic process | GO:0005975: carbohydrate metabolic process GO:0004553: hydrolase activity, hydrolyzing O-glycosyl compounds GO:0003824: catalytic activity GO:0012505: endomembrane system | No |
| AT1G50890 |  | **H** | Binding | GO:0008150: biological process unknown GO:0005488: binding | No |
| AT1G52400 | **BGL1**, BGLU18 (Beta-Glucosidase homolog 1/ Beta Glucosidase 18) | **N** | Member of glycosyl hydrolase family 1, required in inducible ER body formation | GO:0050832: defense response to fungus GO:0003824: catalytic activity GO:0004553 : hydrolase activity, hydrolyzing O-glycosyl compounds GO:0005773: vacuole GO:0009507: chloroplast GO:0043169: cation binding GO:0005777: peroxisome GO:0010168: ER body GO:0005634: nucleus | No |
| AT1G52660 |  | **H** | ATP binding; involved in defense response, apoptosis | GO:0005524: ATP binding GO:0006915: apoptosis GO:0006952: defense response | No |
| AT1G53680 | **GSTU28** (Glutathione S-Transferase Tau 28) | **H** | Glutathione transferase belonging to the tau class of GSTs | GO:0004364: glutathione transferase activity GO:0009407: toxin catabolic process GO:0005737: cytoplasm GO:0046686: response to cadmium ion | No |
| AT1G54970 | **PRP1** (Prolin-rich protein 1) | **H** | Proline-rich protein that is specifically expressed in the root | GO:0005618: cell wall GO:0005199: structural constituent of cell wall | Yes |
| AT1G55020 | **LOX1** (Lipoxygenase 1) | **H** | Lipoxygenase | GO:0009753: response to jasmonic acid stimulus GO:0009816: defense response to bacterium, incompatible interaction GO:0006952: defense response GO:0005575: cellular component unknown GO:0040007: growth GO:0009611: response to wounding GO:0009695: jasmonic acid biosynthetic process GO:0016165: lipoxygenase activity GO:0048364: root development GO:0009737: response to abscisic acid stimulus GO:0030397: membrane disassembly | No |
| AT1G62980 | **EXP18** (Expansin A18) | **H** | Alpha-expansin 18 | GO:0009831: plant-type cell wall modification during multidimensional cell growth GO:0009828: plant-type cell wall loosening GO:0005576: extracellular region GO:0009826: unidimensional cell growth GO:0012505: endomembrane system | Yes |
| AT1G63450 |  | **H** | Catalytic | GO:0016020: membrane GO:0003824: catalytic activity | Yes |
| AT1G63930 |  | **H** | Unknown protein | GO:0008150: biological process unknown GO:0003674: molecular function unknown | No |
| AT1G65180 |  | **H** | DC1 domain-containing protein; involved in intracellular signaling cascade | GO:0007242: intracellular signaling cascade GO:0008270: zinc ion binding GO:0005515: protein binding GO:0005622: intracellular | No |
| AT1G65310 | **XTH17** (Xyloglucan endotrans-glucosylase/ Hydrolase 17) | **N** | Putative xyloglucan endotransglycosylase/hydrolase | GO:0009505: plant-type cell wall GO:0006073: cellular glucan metabolic process GO:0004553: hydrolase activity, hydrolyzing O-glycosyl compounds GO:0005975: carbohydrate metabolic process GO:0016798: hydrolase activity, acting on glycosyl bonds GO:0016762: xyloglucan:xyloglucosyl transferase activity | No |
| AT1G66460 |  | **N** | Protein kinase family protein | GO:0016301: kinase activity GO:0005575: cellular component unknown GO:0006468: protein amino acid phosphorylation GO:0004674: protein serine/threonine kinase activity GO:0004672: protein kinase activity GO:0005524: ATP binding | No |
| AT1G66470 | **RHD6**, bHLH083 | **H** | Basic helix-loop-helix (bHLH) family protein, transcription factor | GO:0003677: DNA binding GO:0003700: transcription factor activity GO:0005739: mitochondrion GO:0045449: regulation of transcription | No |
| AT1G69240 | **MES15** (Methyl esterase 15) | **H** | Encodes a protein predicted to act as a carboxylesterase | GO:0009507: chloroplast GO:0016787: hydrolase activity | Yes |
| AT1G69930 | *GSTU11* (Glutathione S-Transferase Tau 11) | **N** | Glutathione transferase belonging to the tau class of GSTs | GO:0009407: toxin catabolic process GO:0004364: glutathione transferase activity GO:0005737: cytoplasm | No |
| AT1G70460 |  | **H** | Protein kinase, putative | GO:0006468: protein amino acid phosphorylation GO:0005524: ATP binding GO:0016020: membrane GO:0004674: protein serine/threonine kinase activity GO:0004713: protein tyrosine kinase activity GO:0004672: protein kinase activity | Yes |
| AT1G71530 |  | **H** | Protein kinase family protein | GO:0016301: kinase activity GO:0004674: protein serine/threonine kinase activity GO:0004713: protein tyrosine kinase activity GO:0004672: protein kinase activity GO:0006468: protein amino acid phosphorylation GO:0009507: chloroplast GO:0005524: ATP binding GO:0006499: N-terminal protein myristoylation | No |
| AT1G72970 | **HTH**, EDA17 (Hothead/ embryo sac development arrest 17) | **N** | Protein has similarity to the mandelonitrile lyase family of FAD containing oxidoreductases. Transmission of mutant alleles to the progeny shows non mendelian segregation | GO:0005576: extracellular region GO:0050660: FAD binding GO:0016832: aldehyde-lyase activity GO:0009553: embryo sac development GO:0010430: fatty acid omega-oxidation GO:0007267: cell-cell signaling GO:0046593: mandelonitrile lyase activity | No |
| AT1G73860 |  | **H** | ATP binding / microtubule motor; involved in microtubule-based movement | GO:0007018: microtubule-based movement GO:0005875: microtubule associated complex GO:0003777: microtubule motor activity GO:0005524: ATP binding GO:0009507: chloroplast | No |
| AT1G74100 | **SOT16**, CORI-7, ATST5A (Sulfotransferase 16/ Coronatine induced-7) | **N** | Desulfoglucosinolate sulfotransferase, involved in the final step of glucosinolate core structure biosynthesis | GO:0008146: sulfotransferase activity GO:0047364: desulfoglucosinolate sulfotransferase activity GO:0019761: glucosinolate biosynthetic process GO:0005575: cellular component unknown GO:0032260: response to jasmonic acid stimulus during jasmonic acid and ethylene-dependent systemic resistance | No |
| AT1G79320 | **MC6** (Metacaspase 6) | **H** | Cysteine-type endopeptidase activity | GO:0005575: cellular component unknown GO:0006508: proteolysis | Yes |
| AT1G79840 | **GL2** (Glabra 2) | **N** | Transcription factor, homeodomain protein affects epidermal cell identity including trichomes, root hairs, and seed coat | GO:0003700: transcription factor activity GO:0009957: epidermal cell fate specification GO:0005634: nucleus GO:0006355: regulation of transcription, DNA-dependent GO:0010062: negative regulation of trichoblast fate specification GO:000367 : DNA binding | No |
| AT1G79860 | **ROPGEF12** (Rho Guanyl-Nucleotide Exchange Factor 12) | **H** | KPP-like gene family (kinase partner protein) protein, member of the RopGEF (guanine nucleotide exchange factor) family | GO:0009793: embryonic development ending in seed dormancy GO:0005886: plasma membrane GO:0009860: pollen tube growth GO:0005089: Rho guanyl-nucleotide exchange factor activity | No |
| AT2G01090 |  | **H** | Ubiquinol-cytochrome C reductase complex 7.8 kDa protein, putative / mitochondrial hinge protein, putative | GO:0006122: mitochondrial electron transport, ubiquinol to cytochrome c GO:0008121: ubiquinol-cytochrome-c reductase activity GO:0005750: mitochondrial respiratory chain complex III | Yes |
| AT2G01540 |  | **H** | C2 domain-containing protein | GO:0003674: molecular function unknown GO:0005886: plasma membrane GO:0008150: biological process unknown GO:0005773: vacuole | No |
| AT2G02630 |  | **H** | DC1 domain-containing protein | GO:0005575: cellular component unknown GO:0003674: molecular function unknown GO:0008150: biological process unknown | No |
| AT2G03720 | **MRH6** (morphogenesis of root hair 6) | **H** | Involved in root hair development | GO:0048765: root hair cell differentiation GO:0005575: cellular component unknown | Yes |
| AT2G04680 |  | **H** | DC1 domain-containing protein, functions in protein binding, zinc ion binding | GO:0008270: zinc ion binding GO:0005515: protein binding | No |
| AT2G05160 |  | **H** | Zinc finger (CCCH-type) family protein / RNA recognition motif (RRM)-containing protein | GO:0005575: cellular component unknown GO:0008150: biological process unknown GO:0003723: RNA binding GO:0008270: zinc ion binding GO:0000166: nucleotide binding GO:0003676: nucleic acid binding | No |
| AT2G17590 |  | **H** | DC1 domain-containing protein | GO:0005575: cellular component unknown | No |
| AT2G18450 | **SDH1-2** (succinate dehydrogenase 1-2) | **H** | Nuclear encoded mitochondrial flavoprotein subunit of succinate dehydrogenase complex | GO:0005739: mitochondrion GO:0005749: mitochondrial respiratory chain complex II GO:0006121: mitochondrial electron transport, succinate to ubiquinone GO:0000104: succinate dehydrogenase activity | No |
| AT2G18690 |  | **H** | Unknown protein | GO:0016020: membrane GO:0003674: molecular function unknown GO:0008150: biological process unknown | No |
| AT2G20520 | FLA6 (Fascilin-like arabino- galactan 6) | **H** |  | GO:0031225: anchored to membrane | No |
| AT2G21850 |  | **H** | Involved in intracellular signaling cascade | GO:0005575: cellular component unknown GO:0007242: intracellular signaling cascade GO:0008270: zinc ion binding GO:0005515: protein binding | No |
| AT2G22560 |  | **H** | Unknown protein | GO:0005886: plasma membrane GO:0003674: molecular function unknown GO:0008150: biological process unknown | No |
| AT2G24180 | **CYP71B6** (Cytochrome P450 71B6) | **N** | Cytochrome P450 monooxygenase | GO:0016020: membrane GO:0020037: heme binding GO:0005739: mitochondrion GO:0009055: electron carrier activity GO:0004497: monooxygenase activity GO:0005783: endoplasmic reticulum GO:0005886: plasma membrane GO:0019825: oxygen binding GO:0005506: iron ion binding | No |
| AT2G24260 | **bHLH066** | **H** | Basic helix-loop-helix (bHLH) protein that regulates root hair development. | GO:0003700: transcription factor activity GO:0045449: regulation of transcription GO:0005634: nucleus GO:0003677: DNA binding | No |
| AT2G26870 |  | **N** | Phosphoesterase family proteins; involved in triglyceride biosynthetic process, phospholipid biosynthetic process | GO:0019432: triglyceride biosynthetic process GO:0008654: phospholipid biosynthetic process GO:0012505: endomembrane system GO:0016788: hydrolase activity, acting on ester bonds | No |
| AT2G28440 |  | **H** | Proline-rich family protein | GO:0012505: endomembrane system | No |
| AT2G29740 | **UGT71C2** (UDP-Glucosyl Transferase 71C2) | **H** | UDP-Glucosyl Transferase 71C2 | GO:0005575: cellular component unknown GO:0016757: transferase activity, transferring glycosyl groups GO:0008194: UDP-glycosyltransferase activity GO:0008152: metabolic process | No |
| AT2G32280 |  | **N** | Unknown protein | GO:0012505: endomembrane system GO:0003674: molecular function unknown GO:0008150: biological process unknown | No |
| AT2G34910 |  | **H** | Unknown protein | GO:0005575: cellular component unknown GO:0003674: molecular function unknown GO:0008150: biological process unknown | No |
| AT2G35585 |  | **N** | Unknown protein | GO:0005575: cellular component unknown GO:0003674: molecular function unknown GO:0008150: biological process unknown | No |
| AT2G37260 | **TTG2**, WRKY44 (Transparent Testa Glabra 2) | **N** | Protein similar to WRKY transcription factors | GO:0003700: transcription factor activity GO:0006355: regulation of transcription, DNA-dependent GO:0005634: nucleus GO:0010214: seed coat development GO:0009957: epidermal cell fate specification | No |
| AT2G37440 |  | **H** | Endonuclease/exonuclease/phosphatase family protein | GO:0008150: biological process unknown GO:0016787: hydrolase activity GO:0004437: inositol or phosphatidylinositol phosphatase activity | No |
| AT2G37670 |  | **H** | WD-40 repeat family protein; involved in signal transduction | GO:0007165: signal transduction GO:0004871: signal transducer activity GO:0005834: heterotrimeric G-protein complex | No |
| AT2G39690 |  | **H** | Unknown protein | GO:0005739: mitochondrion GO:0003674: molecular function unknown GO:0008150: biological process unknown | Yes |
| AT2G40010 |  | **H** | 60S acidic ribosomal protein P0 (RPP0A) | GO:0003735: structural constituent of ribosome GO:0022626: cytosolic ribosome GO:0042254: ribosome biogenesis GO:0006412: translation GO:0006414: translational elongation GO:0005840: ribosome | No |
| AT2G41380 |  | **N** | Embryo-abundant protein-related; functions in methyltransferase activity | GO:0046686: response to cadmium ion GO:0005739: mitochondrion GO:0008168: methyltransferase activity | No |
| AT2G41970 |  | **H** | Protein kinase, putative | GO:0005575: cellular component unknown GO:0006468: protein amino acid phosphorylation GO:001630: kinase activity 1 GO:0005524: ATP binding GO:0004713: protein tyrosine kinase activity GO:0004672: protein kinase activity | No |
| AT2G42060 |  | **N** | CHP-rich zinc finger protein, putative; involved in intracellular signaling cascade | GO:0005575: cellular component unknown GO:0007242: intracellular signaling cascade | No |
| AT2G45220 |  | **N** | Pectinesterase family protein; involved in cell wall modification | GO:0030599: pectinesterase activity GO:0004857: enzyme inhibitor activity GO:0009505: plant-type cell wall GO:0016020: membrane GO:0042545: cell wall modification | No |
| AT2G45750 |  | **H** | Dehydration-responsive family protein | GO:0008150: biological process unknown | No |
| AT2G45890 | **ROPGEF4** (RHO Guanyl-Nucleotide exchange factor 4) | **H** | KPP-like (kinase partner protein) protein, member of the RopGEF (guanine nucleotide exchange factor) family | GO:0005089: Rho guanyl-nucleotide exchange factor activity GO:0008150: biological process unknown GO:0009507: chloroplast | Yes |
| AT2G46860 | **PPa3** (Arabidopsis thaliana pyrophosphorylase 3) | **H** | Encodes a protein that might have inorganic pyrophosphatase activity | GO:0016462: pyrophosphatase activity GO:0004427: inorganic diphosphatase activity GO:0005737: cytoplasm GO:0006796: phosphate metabolic process GO:0016020: membrane GO:0008152: metabolic process | Yes |
| AT2G47540 |  | **H** | Pollen Ole e 1 allergen and extensin family protein | GO:0012505: endomembrane system GO:0003674: molecular function unknown GO:0008150: biological process unknown | No |
| AT2G48080 |  | **H** | Oxidoreductase, 2OG-Fe(II) oxygenase family protein | GO:0016491: oxidoreductase activity GO:0008150: biological process unknown GO:0005575: cellular component unknown | No |
| AT3G01930 |  | **N** | Nodulin family protein |  | No |
| AT3G03520 |  | **H** | Phosphoesterase family protein | GO:0016788: hydrolase activity, acting on ester bonds GO:0016020: membrane GO:0005773: vacuole GO:0008150: biological process unknown | No |
| AT3G04940 | **CYSD1** (Cysteine Synthase D1) | **N** | Cysteine synthase CysD1 | GO:0004124: cysteine synthase activity GO:0019344: cysteine biosynthetic process GO:0004124: cysteine synthase activity | No |
| AT3G05170 |  | **H** | Phosphoglycerate/bisphosphoglycerate mutase family protein | GO:0003824: catalytic activity GO:0008152: metabolic process GO:0005575: cellular component unknown | No |
| AT3G07070 |  | **H** | Protein kinase family protein | GO:0005524: ATP binding GO:0016301: kinase activity GO:0004672: protein kinase activity GO:0005575: cellular component unknown GO:0004674: protein serine/threonine kinase activity GO:0006468: protein amino acid phosphorylation | No |
| AT3G07880 |  | **H** | Rho GDP-dissociation inhibitor family protein | GO:0010053: root epidermal cell differentiation GO:0005737: cytoplasm GO:0005094: Rho GDP-dissociation inhibitor activity GO:0009932: cell tip growth | No |
| AT3G10710 |  | **H** | Pectinesterase family protein; involved in cell wall modification | GO:0042545: cell wall modification GO:0009505: plant-type cell wall GO:0004857: enzyme inhibitor activity GO:0005618: cell wall GO:0030599: pectinesterase activity | Yes |
| AT3G12540 |  | **H** | Unknown protein | GO:0008150: biological process unknown GO:0005575: cellular component unknown | Yes |
| AT3G13782 | NFA04, NAP1;4, NFA4 (Nucleosome assembly protein1;4) | **H** | Functions in chromatin binding, DNA binding; involved nucleosome assembly, nucleotide-excision repair | GO:0005634: nucleus GO:0046686: response to cadmium ion GO:0006289: nucleotide-excision repair GO:0003677: DNA binding GO:0003682: chromatin binding GO:0006334: nucleosome assembly GO:0005737: cytoplasm | No |
| AT3G15760 |  | **H** | Unknown protein | GO:0008150: biological process unknown GO:0005575: cellular component unknown GO:0012505: endomembrane system | No |
| AT3G16800 |  | **N** | Protein phosphatase 2C, putative / PP2C, putative | GO:0008150: biological process unknown GO:0005634: nucleus GO:0003824: catalytic activity GO:0004722: protein serine/threonine phosphatase activity GO:0005737: cytoplasm | No |
| AT3G19320 |  | **N** | Leucine-rich repeat family protein | GO:0005515: protein binding GO:0012505: endomembrane system | No |
| AT3G23190 |  | **H** | Lesion inducing protein-related | GO:0005783: endoplasmic reticulum GO:0003674: molecular function unknown GO:0008150: biological process unknown | No |
| AT3G26744 | **ICE1**, SCRM (Inducer of CBF expression) | **N** | Similar to basic helix-loop-helix (bHLH) family protein / F-box family protein from Arabidopsis thaliana; similar to transcription factor ICE1-like from Oryza sativa (japonica cultivar-group) | GO:0003677: DNA binding GO:0016567: protein ubiquitination GO:0003700: transcription factor activity GO:0005634: nucleus GO:0009409: response to cold GO:0045941: positive regulation of transcription GO:0010440: stomatal lineage progression GO:0016563: transcription activator activity GO:0050826: response to freezing | No |
| AT3G28910 | **MYB30**, ATMYB30 | **N** | Transcription factor myb homologue | GO:0009723: response to ethylene stimulus GO:0009617: response to bacterium GO:0009739: response to gibberellin stimulus GO:0009753: response to jasmonic acid stimulus GO:0003700: transcription factor activity GO:0009733: response to auxin stimulus GO:0003677: DNA binding GO:0009751: response to salicylic acid stimulus GO:0009626: plant-type hypersensitive response GO:0042761: very-long-chain fatty acid biosynthetic process | No |
| AT3G29410 |  | **N** | Terpene synthase/cyclase family protein | GO:0008152: metabolic process GO:0000287: magnesium ion binding GO:0016829: lyase activity | No |
| AT3G46760 |  | **H** | Protein kinase family protein | GO:0005524: ATP binding GO:0005575: cellular component unknown GO:0004672: protein kinase activity GO:0016301: kinase activity GO:0006468: protein amino acid phosphorylation GO:0004674: protein serine/threonine kinase activity | No |
| AT3G47340 | **ASN1**, DIN6 (Glutamine-dependent Asparagine Synthase 1) | **H** | Asparagine synthase (glutamine-hydrolyzing) | GO:0009063: cellular amino acid catabolic process GO:0009744: response to sucrose stimulus GO:0009416: response to light stimulus GO:0043617: cellular response to sucrose starvation GO:0004066: asparagine synthase (glutamine-hydrolyzing) activity GO:0009749: response to glucose stimulus GO:0009646response to absence of light GO:0009750: response to fructose stimulus | No |
| AT3G49220 |  | **N** | Pectinesterase family protein; involved in cell wall modification; | GO:0005618: cell wall GO:0042545: cell wall modification GO:0004857: enzyme inhibitor activity GO:0009505: plant-type cell wall GO:0030599: pectinesterase activity | No |
| AT3G49960 |  | **H** | Peroxidase, putative; involved in response to oxidative stress | GO:0004601: peroxidase activity GO:0012505: endomembrane system GO:0009055: electron carrier activity GO:0020037: heme binding GO:0006979: response to oxidative stress | No |
| AT3G54040 |  | **H** | Photoassimilate-responsive protein-related | GO:0008150: biological process unknown GO:0012505: endomembrane system GO:0003674: molecular function unknown | Yes |
| AT3G54140 | **PTR1**, ATPTR1, (Peptide Transporter 1) | **N** | Encodes a di- and tri-peptide transporter that recognizes a variety of different amino acid combinations. | GO:0005215: transporter activity GO:0005886: plasma membrane GO:0016020: membrane GO:0042938: dipeptide transport GO:0042936: dipeptide transporter activity GO:0042937: tripeptide transporter activity GO:0006857: oligopeptide transport GO:0042939: tripeptide transport GO:0006807: nitrogen compound metabolic process | No |
| AT3G54400 |  | **N** | Aspartyl protease family protein; functions in aspartic-type endopeptidase activity | GO:0005618: cell wall GO:0048046: apoplast GO:0006508: proteolysis GO:0009505: plant-type cell wall GO:0009507: chloroplast | No |
| AT3G54580 |  | **H** | Proline-rich extensin-like family protein; structural constituent of cell wall | GO:0012505: endomembrane system GO:0009664: plant-type cell wall organization GO:0005199: structural constituent of cell wall | No |
| AT3G54870 | **MRH2**, ARK1, CAE1 (Morphogenesis of root hair2) | **H** | Armadillo-repeat containing kinesin-related protein. Plays a role during transition to root-hair tip growth | GO:0003777: microtubule motor activity GO:0048768: root hair cell tip growth GO:0031110: regulation of microtubule polymerization or depolymerization GO:0005875: microtubule associated complex GO:0048765: root hair cell differentiation GO:0009507: chloroplast GO:0008017: microtubule binding GO:0003779: actin binding | No |
| AT3G56000 | **CSLA14**, (Cellulose synthase like A14) | **H** | Gene similar to cellulose synthase | GO:0008150 : biological process unknown GO:0016757: transferase activity, transferring glycosyl groups GO:0005575: cellular component unknown GO:0016759: cellulose synthase activity | No |
| AT3G60330 | **AHA7** (Arabidopsis H+ ATPase) | **H** | Arabidopsis H(+)-ATPase; ATP biosynthetic process | GO:0006812: cation transport GO:0008553: hydrogen-exporting ATPase activity, phosphorylative mechanism GO:0006754: ATP biosynthetic process GO:0005886: plasma membrane GO:0008152: metabolic process | No |
| AT3G61820 |  | **N** | Aspartyl protease family protein; aspartic-type endopeptidase activity | GO:0006508: proteolysis GO:0009505: plant-type cell wall GO:0006508: proteolysis | No |
| AT3G62680 | **PRP3** | **H** | PRP3 (PROLINE-RICH PROTEIN 3); structural constituent of cell wall | GO:0005618: cell wall GO:0005199: structural constituent of cell wall GO:0010054: trichoblast differentiation | Yes |
| AT4G00480 | **MYC1,** bHLH012 | **H** | MYC-related protein with a basic helix-loop-helix motif at the C-terminus and a region similar to the maize B/R family at the N-terminus | GO:0003700: transcription factor activity GO:0005634: nucleus GO:0003677: DNA binding GO:0045449: regulation of transcription | No |
| AT4G00680 | **ADF8** | **H** | Actin depolymerizing factor 8 (ADF8); functions in actin binding | GO:0005622: intracellular GO:0008150: biological process unknown GO:0003779: actin binding | No |
| AT4G02270 |  | **H** | Pollen Ole e 1 allergen and extensin family protein | GO:0008150: biological process unknown GO:0012505: endomembrane system GO:0003674: molecular function unknown | Yes |
| AT4G02390 | **APP,** PARP1, ( Poly (ADP-Ribose) Polymerase) | **H** | DNA dependent nuclear poly (ADP-ribose) polymerase, thought to be involved in post-translational modification | GO:0006471: protein amino acid ADP-ribosylation GO:0003950: NAD+ ADP-ribosyltransferase activity GO:0005634: nucleus GO:0006471: protein amino acid ADP-ribosylation GO:0003676: nucleic acid binding | Yes |
| AT4G03330 | **SYP123** (Syntaxin of plants 123) | **H** | Member of SYP12 Gene Family | GO:0006886: intracellular protein transport GO:0016020: membrane GO:0005484: SNAP receptor activity GO:0006944: membrane fusion | No |
| AT4G07960 | **CSLC12** (Cellulose-Synthase like C12) | **H** | Gene similar to cellulose synthase | GO:0016757: transferase activity, transferring glycosyl groups GO:0008150: biological process unknown GO:0016759: cellulose synthase activity GO:0005575: cellular component unknown | No |
| AT4G09990 |  | **H** | Unknown protein | GO:0008150: biological process unknown GO:0012505: endomembrane system GO:0003674: molecular function unknown | No |
| AT4G12330 | **CYP706A7** (Cytochrome P450, Family 706, Subfamily A, Polypeptide 7) | **H** | Member of CYP706A | GO:0009055: electron carrier activity GO:0004497: monooxygenase activity GO:0005575: cellular component unknown GO:0019825: oxygen binding GO:0020037: heme binding GO:0005506: iron ion binding | No |
| AT4G13390 |  | **H** | Proline-rich extensin-like family protein; structural constituent of cell wall | GO:0005199: structural constituent of cell wall GO:0009664: plant-type cell wall organization GO:0012505: endomembrane system | No |
| AT4G14610 |  | **H** | Pseudogene, disease resistance protein (CC-NBS-LRR class), putative | GO:0005515: protein binding GO:0006499: N-terminal protein myristoylation | No |
| AT4G14980 |  | **H** | Involved in intracellular signaling cascade | GO:0007242: intracellular signaling cascade | No |
| AT4G15390 |  | **H** | Transferase family protein | GO:0016747: transferase activity, transferring acyl groups other than amino-acyl groups GO:0005575: cellular component unknown GO:0016740: transferase activity GO:0008150: biological process unknown | No |
| AT4G16190 |  | **H** | Cysteine proteinase, putative | GO:0005773: vacuole GO:0006508: proteolysis | No |
| AT4G16350 | **CBL6**, SCABP2 (Calcineurin B-like Protein 6) | **H** | Calcium sensor protein. Binds CIPK14 | GO:0005509: calcium ion binding GO:0019722: calcium-mediated signaling GO:0012505: endomembrane system | No |
| AT4G17215 |  | **N** | Unknown protein | GO:0005575: cellular component unknown GO:0003674: molecular function unknown GO:0008150: biological process unknown | No |
| AT4G17970 |  | **N** | Unknown protein | GO:0005575: cellular component unknown GO:0008150: biological process unknown | No |
| AT4G18640 | **MRH1** (Morphogenesis of root hair 1) | **H** | Protein kinase, required for root hair elongation during tip growth | GO:0048765: root hair cell differentiation GO:0005515: protein binding GO:0005524: ATP binding GO:0007169: transmembrane receptor protein tyrosine kinase signaling pathway GO:0012505: endomembrane system GO:0004674: protein serine/threonine kinase activity GO:0006468: protein amino acid phosphorylation GO:0004713: protein tyrosine kinase activity GO:0004672: protein kinase activity | No |
| AT4G19680 | **IRT2** (iron regulated transporter 2) | **H** | Iron transporter | GO:0016020: membrane GO:0005385: zinc ion transmembrane transporter activity GO:0016021: integral to membrane GO:0005381: iron ion transmembrane transporter activity GO:0006826: iron ion transport GO:0006829: zinc ion transport GO:0012505: endomembrane system | No |
| AT4G20480 |  | **H** | Unknown protein | GO:0005575: cellular component unknown GO:0008150: biological process unknown | No |
| AT4G22280 |  | **N** | F-box family protein | GO:0005575: cellular component unknown GO:0003674: molecular function unknown GO:0008150: biological process unknown | No |
| AT4G22640 |  | **H** | Unknown protein | GO:0031225: anchored to membrane GO:0003674: molecular function unknown GO:0008150: biological process unknown | No |
| AT4G24580 | **REN1** (ROP1 Enhancer 1) | **H** | Rho GTPase-activating protein that interacts with ROP1 (a Rho GTPase) and regulates pollen tube development | GO:0005100: Rho GTPase activator activity GO:0045177: apical part of cell GO:0007165: signal transduction GO:0035091: phosphoinositide binding GO:0009865: pollen tube adhesion GO:0017048: Rho GTPase binding GO:0032862: activation of Rho GTPase activity GO:0035024: negative regulation of Rho protein signal transduction GO:0009846: pollen germination GO:0005938: cell cortex GO:0048868: pollen tube development | Yes |
| AT4G25160 |  | **H** | Protein kinase family protein | GO:0006468: protein amino acid phosphorylation GO:0004672: protein kinase activity GO:0000151: ubiquitin ligase complex GO:0004674: protein serine/threonine kinase activity GO:0006950: response to stress GO:0004842: ubiquitin-protein ligase activity GO:0016301: kinase activity GO:0005524: ATP binding GO:0016567: protein ubiquitination | No |
| AT4G25220 |  | **H** | Transporter, putative | GO:0006810: transport GO:0005215: transporter activity | Yes |
| AT4G25790 |  | **H** | Allergen V5/Tpx-1-related family protein | GO:0003674: molecular function unknown GO:0008150: biological process unknown GO:0005576: extracellular region GO:0012505: endomembrane system | No |
| AT4G25820 | **XTR9** (Xyloglucan endotransglycosylase 9) | **H** | Xyloglucan endotransglycosylase with a clear preference for non-fucosylated xyloglucan polymer | GO:0006073: cellular glucan metabolic process GO:0016762: xyloglucan:xyloglucosyl transferase activity GO:0012505: endomembrane system GO:0005975: carbohydrate metabolic process GO:0005618: cell wall GO:0048046: apoplast GO:0004553: hydrolase activity, hydrolyzing O-glycosyl compounds GO:0016798: hydrolase activity, acting on glycosyl bonds | No |
| AT4G26320 | **AGP13** (Arabinogalactan protein 13) | **N** |  | GO:0031225: anchored to membrane | No |
| AT4G26770 |  | **H** | Phosphatidate cytidylyltransferase/ transferase; involved in phospholipid biosynthetic process | GO:0008654: phospholipid biosynthetic process GO:0016020: membrane GO:0016772: transferase activity, transferring phosphorus-containing groups GO:0004605: phosphatidate cytidylyltransferase activity | No |
| AT4G27290 |  | **H** | ATP binding / protein kinase/ protein serine/threonine kinase/ protein tyrosine kinase/ sugar binding | GO:0005529: sugar binding GO:0004713: protein tyrosine kinase activity GO:0048544: recognition of pollen GO:0006468: protein amino acid phosphorylation GO:0005524: ATP binding GO:0004672: protein kinase activity GO:0012505: endomembrane system GO:0004672: protein kinase activity GO:0004674: protein serine/threonine kinase activity | No |
| AT4G28850 |  | **H** | Xyloglucan:xyloglucosyl transferase, putative / xyloglucan endotransglycosylase, putative / endo-xyloglucan transferase, putative | GO:0016798: hydrolase activity, acting on glycosyl bonds GO:0006073: cellular glucan metabolic process GO:0004553: hydrolase activity, hydrolyzing O-glycosyl compounds GO:0016762: xyloglucan:xyloglucosyl transferase activity GO:0012505: endomembrane system GO:0048046: apoplast GO:0005975: carbohydrate metabolic process GO:0005618: cell wall | No |
| AT4G29180 |  | **H** | Leucine-rich repeat protein kinase, putative | GO:0016301: kinase activity GO:0012505: endomembrane system GO:0006468: protein amino acid phosphorylation | Yes |
| AT4G29800 | **PLP8**, PLA IVD (Patatin like Protein 8) | **H** | Involved in lipid metabolic process | GO:0006629: lipid metabolic process GO:0005575: cellular component unknown GO:0008152: metabolic process | No |
| AT4G30560 | **CNGC9** , ATCNGC9, (Cyclic nucleotide gated channel )) | **H** | Member of Cyclic nucleotide gated channel family; ion channel | GO:0005516: calmodulin binding GO:0006813: potassium ion transport GO:0030551: cyclic nucleotide binding GO:0006811: ion transport GO:0005216: ion channel activity GO:0016020: membrane | No |
| AT4G31250 |  | **H** | Leucine-rich repeat transmembrane protein kinase | GO:0007169: transmembrane receptor protein tyrosine kinase signaling pathway GO:0012505: endomembrane system GO:0016301: kinase activity GO:0004674: protein serine/threonine kinase activity GO:0005524: ATP binding GO:0006468: protein amino acid phosphorylation | No |
| AT4G32460 |  | **N** | Unknown protein | GO:0003674: molecular function unknown GO:0008150: biological process unknown GO:0009505: plant-type cell wall | No |
| AT4G33730 |  | **H** | Pathogenesis-related protein, putative | GO:0003674: molecular function unknown GO:0008150: biological process unknown GO:0005576: extracellular region GO:0012505: endomembrane system | No |
| AT4G34580 | **COW1**, SRH1 (Can of Worms 1) | **H** | Phosphatidylinositol transfer protein essential for root hair tip growth | GO:0005215: transporter activity GO:0005622: intracellular GO:0008526: phosphatidylinositol transporter activity GO:0048768: root hair cell tip growth GO:0010053: root epidermal cell differentiation GO:0006810: transport GO:0009932: cell tip growth | No |
| AT4G34590 | **GBF6,** ATB2, BZIP11 (G-Box binding factor 6) | **N** | Basic domain leucine zipper (bZip) transcription factor | GO:0017148: negative regulation of translation GO:0003677: DNA binding GO:0003700: transcription factor activity GO:0006355: regulation of transcription, DNA-dependent GO:0005634: nucleus GO:0009744: response to sucrose stimulus GO:0046982: protein heterodimerization activity GO:0009507: chloroplast | No |
| AT4G40090 | AGP3 (Arabinogalactan-Protein 3) | **H** | Arabinogalactan-protein, involved in multicellular organismal development | GO:0007275: multicellular organismal development GO:0012505: endomembrane system | No |
| AT5G01610 |  | **H** | unknown protein | GO:0003674: molecular function unknown GO:0008150: biological process unknown | No |
| AT5G04960 |  | **H** | Pectinesterase family protein; involved in cell wall modification | GO:0004857: enzyme inhibitor activity GO:0030599: pectinesterase activity GO:0009505: plant-type cell wall GO:0042545: cell wall modification GO:0005618: cell wall | No |
| AT5G05400 |  | **H** | Disease resistance protein (CC-NBS-LRR class), putative; involved in N-terminal protein myristoylation, defense response, apoptosis | GO:0005515: protein binding GO:0009507: chloroplast GO:0005524: ATP binding GO:0006499: N-terminal protein myristoylation GO:0006915: apoptosis GO:0006952: defense response | No |
| AT5G05500 |  | **H** | Pollen Ole e 1 allergen and extensin family protein; | GO:0008150: biological process unknown GO:0012505: endomembrane system GO:0003674: molecular function unknown | No |
| AT5G06640 |  | **H** | Proline-rich extensin-like family protein; structural constituent of cell wall | GO:0005199: structural constituent of cell wall GO:0009664: plant-type cell wall organization | No |
| AT5G07080 |  | **H** | Transferase family protein | GO:0016747: transferase activity, transferring acyl groups other than amino-acyl groups GO:0005575: cellular component unknown GO:0008150: biological process unknown | No |
| AT5G10520 | **RBK1** (Rop Binding protein kinases 1) | **N** | Protein serine/threonine kinase, involved in defense response, incompatible interaction | GO:0009814: defense response, incompatible interaction GO:0004672: protein kinase activity GO:0005524: ATP binding GO:0004674: protein serine/threonine kinase activity GO:0005829: cytosol GO:0016301: kinase activity GO:0012505: endomembrane system | No |
| AT5G11020 |  | **N** | ATP binding / kinase/ protein kinase/ protein serine/threonine kinase/ protein tyrosine kinase | GO:0006468: protein amino acid phosphorylation GO:0005524: ATP binding GO:0004674: protein serine/threonine kinase activity GO:0004713: protein tyrosine kinase activity GO:0004672: protein kinase activity GO:0016301: kinase activity | No |
| AT5G13150 | **EXO70C1** (exocyst subunit EX070 family protein C1) | **H** | A member of EXO70 gene family, putative exocyst subunits | GO:0000145: exocyst GO:0006904: vesicle docking during exocytosis GO:0005515: protein binding GO:0006887: exocytosis | No |
| AT5G13990 | **EXO70C2** | **H** | A member of EXO70 gene family, putative exocyst subunits | GO:0005515: protein binding GO:0006887: exocytosis GO:0006904: vesicle docking during exocytosis GO:0000145: exocyst | Yes |
| AT5G15890 |  | **H** | Unknown protein | GO:0008150: biological process unknown | No |
| AT5G15950 |  | **H** | Adenosylmethionine decarboxylase family protein; involved in spermidine biosynthetic process, spermine biosynthetic process | GO:0004014: adenosylmethionine decarboxylase activity GO:0008295: spermidine biosynthetic process GO:0006597: spermine biosynthetic process GO:0005575: cellular component unknown GO:0006596: polyamine biosynthetic process | No |
| AT5G16030 |  | **N** | Unknown protein | GO:0005575: cellular component unknown GO:0003674: molecular function unknown GO:0008150: biological process unknown | No |
| AT5G16900 |  | **H** | Leucine-rich repeat protein kinase, putative | GO:0016301: kinase activity GO:0006468: protein amino acid phosphorylation GO:0012505: endomembrane system | No |
| AT5G19800 |  | **H** | Hydroxyproline-rich glycoprotein family protein | GO:0008150: biological process unknown GO:0012505: endomembrane system | No |
| AT5G22410 |  | **H** | Peroxidase, putative; involved in response to oxidative stress | GO:0009055: electron carrier activity GO:0012505: endomembrane system GO:0004601: peroxidase activity GO:0020037: heme binding GO:0006979: response to oxidative stress | Yes |
| AT5G23030 | **TET12** (Tetraspanin 12) | **H** | Member of TETRASPANIN family | GO:0007568: aging GO:0012505: endomembrane system GO:0003674: molecular function unknown GO:0016021: integral to membrane | No |
| AT5G24140 | **SQP2** (Squalene monoxygenases2) | **H** | Encodes a protein with similarity to squalene monoxygenases | GO:0004506: squalene monooxygenase activity GO:0016491: oxidoreductase activity GO:0016126: sterol biosynthetic process GO:0016021: integral to membrane GO:0012505: endomembrane system GO:0050660: FAD binding | No |
| AT5G24310 | **ABIL3** (ABL interactor-like protein 3) | **H** |  | GO:0005575: cellular component unknown GO:0003674: molecular function unknown GO:0008150: biological process unknown | No |
| AT5G35190 |  | **H** | Proline-rich extensin-like family protein; structural constituent of cell wall | GO:0012505: endomembrane system GO:0005199: structural constituent of cell wall GO:0009664: plant-type cell wall organization | No |
| AT5G40510 |  | **H** | Unknown protein | GO:0005575: cellular component unknown GO:0003674: molecular function unknown GO:0008150: biological process unknown | No |
| AT5G40860 |  | **H** | Unknown protein | GO:0003674: molecular function unknown GO:0008150: biological process unknown | No |
| AT5G40960 |  | **N** | Unknown protein | GO:0031225: anchored to membrane GO:0003674: molecular function unknown GO:0008150: biological process unknown | No |
| AT5G45800 | **MEE62** (maternal effect embryo arrest 62) | **N** | Protein kinase, involved in embryonic development ending in seed dormancy, transmembrane receptor protein tyrosine kinase signaling pathway | GO:0004674: protein serine/threonine kinase activity GO:0009793: embryonic development ending in seed dormancy GO:0005515: protein binding GO:0007169: transmembrane receptor protein tyrosine kinase signaling pathway GO:0005524: ATP binding GO:0006468: protein amino acid phosphorylation GO:0004713: protein tyrosine kinase activity GO:0004672: protein kinase activity | No |
| AT5G46250 |  | **H** | RNA recognition motif (RRM)-containing protein; involved in RNA processing | GO:0030529: ribonucleoprotein complex GO:0000166: nucleotide binding GO:0003676: nucleic acid binding GO:0006396: RNA processing GO:0005634: nucleus GO:0003723: RNA binding | No |
| AT5G46670 |  | **N** | CHP-rich zinc finger protein, putative | GO:0008270: zinc ion binding GO:0005575: cellular component unknown GO:0008150: biological process unknown GO:0005515: protein binding | No |
| AT5G47550 |  | **N** | Cysteine protease inhibitor, putative | GO:0008150: biological process unknown GO:0005618: cell wall GO:0004869: cysteine-type endopeptidase inhibitor activity | No |
| AT5G49080 |  | **H** | Transposable element gene; similar to proline-rich extensin-like family protein |  | No |
| AT5G49270 | **COBL9**, SHV2, DER9, MRH4, (Cobra like 9/ Shaven2) | **H** | Involved in successfully establishing tip growth in root hairs | GO:0031225: anchored to membrane GO:0010053: root epidermal cell differentiation GO:0048765: root hair cell differentiation GO:0009932: cell tip growth | No |
| AT5G53200 | **TRY** (Triptychon) | **N** | Transcription factor | GO:0003700: transcription factor activity GO:0010091: trichome branching GO:0003677: DNA binding GO:0045449: regulation of transcription | No |
| AT5G54840 | **SGP1**, ATSGP1 | **N** | Monomeric G protein | GO:0005525: GTP binding GO:0007264: small GTPase mediated signal transduction GO:0005622: intracellular | No |
| AT5G57530 |  | **H** | Xyloglucan:xyloglucosyl transferase, putative / xyloglucan endotransglycosylase, putative / endo-xyloglucan transferase, putative; involved in carbohydrate metabolic process, cellular glucan metabolic process | GO:0004553: hydrolase activity, hydrolyzing O-glycosyl compounds GO:0006073: cellular glucan metabolic process GO:0016798: hydrolase activity, acting on glycosyl bonds GO:0005975: carbohydrate metabolic process GO:0005618: cell wall GO:0005737: cytoplasm GO:0016762: xyloglucan:xyloglucosyl transferase activity | No |
| AT5G57540 |  | **H** | Xyloglucan:xyloglucosyl transferase, putative / xyloglucan endotransglycosylase, putative / endo-xyloglucan transferase, putative; involved in carbohydrate metabolic process, cellular glucan metabolic process | GO:0016798: hydrolase activity, acting on glycosyl bonds GO:0006073: cellular glucan metabolic process GO:0016762: xyloglucan:xyloglucosyl transferase activity GO:0004553: hydrolase activity, hydrolyzing O-glycosyl compounds GO:0005975: carbohydrate metabolic process GO:0005618: cell wall GO:0012505: endomembrane system GO:0048046: apoplast | No |
| AT5G58010 | **bHLH082** | **H** | Basic helix-loop-helix (bHLH) protein that regulates root hair developmen | GO:0045449: regulation of transcription GO:0003700: transcription factor activity GO:0005634: nucleus GO:0003677: DNA binding | No |
| AT5G61350 |  | **H** | Protein kinase family protein | GO:0016301: kinase activity GO:0012505: endomembrane system GO:0006468: protein amino acid phosphorylation | No |
| AT5G61550 |  | **H** | Protein kinase family protein; ubiquitin-protein ligase activity, protein serine/threonine kinase activity | GO:0004672: protein kinase activity GO:0004674: protein serine/threonine kinase activity GO:0006468: protein amino acid phosphorylation GO:0004842: ubiquitin-protein ligase activity GO:0005524: ATP binding GO:0000151: ubiquitin ligase complex GO:0016301: kinase activity GO:0016567: protein ubiquitination | No |
| AT5G61650 | **CYCP4;2**, CYCP4 | **H** | The P-type cyclins (CYCPs), cyclin-dependent protein kinase | GO:0005575: cellular component unknown GO:0051726: regulation of cell cycle GO:0004693: cyclin-dependent protein kinase activity | No |
| AT5G62310 | **IRE** (Incomplete root hair elongation) | **H** | Protein with a serine/threonine kinase domain | GO:0005575: cellular component unknown GO:0009826: unidimensional cell growth GO:0016301: kinase activity GO:0004674: protein serine/threonine kinase activity | No |
| AT5G63590 | **FLS3** (Flavonol Synthase 3) | **N** | Flavonol synthase involved in response to light stimulus, response to sucrose stimulus, flavonoid biosynthetic process | GO:0005575: cellular component unknown GO:0009416: response to light stimulus GO:0045431: flavonol synthase activity GO:0009813: flavonoid biosynthetic process | No |
| AT5G65090 | **MRH3**, BST1, DER4 (Morphology of root hairs 3/ Bristled 1) | **H** | Protein involved in root hair morphogenesis and tip growth. Required for restricting both the size of the root-hair initiation site and the width of the root hairs during the transition to tip growth. | GO:0005575: cellular component GO:0004437: inositol or phosphatidylinositol phosphatase activity GO:0009932: cell tip growth GO:0010053: root epidermal cell differentiation GO:0016787: hydrolase activity GO:0048765: root hair cell differentiation | No |
| AT5G65160 |  | **H** | Tetratricopeptide repeat (TPR)-containing protein | GO:0005488: binding GO:0005575: cellular component unknown GO:0008150: biological process unknown | Yes |
| AT5G66590 |  | **N** | Allergen V5/Tpx-1-related family protein | GO:0005576: extracellular region GO:0012505: endomembrane system GO:0003674: molecular function unknown GO:0008150: biological process unknown | Yes |
| AT5G67400 | **PER73** (Peroxidase 73) | **H** | Proxidase involved in response to oxidative stress | GO:0004601: peroxidase activity GO:0006979: response to oxidative stress GO:0012505: endomembrane system GO:0009055: electron carrier activity GO:0020037: heme binding | Yes |

* An “H” indicates preferential transcript accumulation in the hairy mutants; an “N” indicates preferential transcript accumulation in the hairless mutant (*cpc try*).

** “Yes” indicates that an RHE exists within 1kb upstream of the gene’s AUG start codon, based on data from Won et al 2009.
