# Supplementary material for: A Gene Regulatory Network for Root Epidermis Cell Differentiation in Arabidopsis
Source: PLoS Genet. 2012 Jan 12;8(1):e1002446. doi: 10.1371/journal.pgen.1002446 (PMC3257299; doi:10.1371/journal.pgen.1002446)
Supplement: Table S6 — Gene Ontology (GO) terms overrepresented among the genes significantly affected by mutations in downstream genes. (DOCX) [file pgen.1002446.s014.docx]

**Table S6.** Gene Ontology (GO) Terms Overrepresented Among the Genes Significantly Affected by Mutations in Downstream Genes*.

| **Mutant** | **p-value** | **Gene Ontology (GO) Class** | **Genes** |
| --- | --- | --- | --- |
| *bhlh66* | 0.0052 | 0005618 cell wall | AT5G47550, AT3G54400, AT4G28850 |
| *cobl9* | 6.20E-07 | 0006073 cellular glucan metabolic process | AT5G57530, AT4G28850, AT5G57540, AT1G10550 |
|  | 3.90E-06 | 0016798 hydrolase activity, acting on glycosyl bonds | AT5G57530, AT4G28850, AT5G57540, AT1G10550 |
|  | 0.0025 | 0005618 cell wall | AT5G57530, AT4G28850, AT5G57540, AT1G10550 |
|  | 0.0026 | 0005975 carbohydrate metabolic process | AT5G57530, AT4G28850, AT5G57540, AT1G10550 |
|  | 0.0239 | 0048046 apoplast | AT4G28850, AT5G57540, AT1G10550 |
| *cow1* | 6.60E-04 | 0007242 intracellular signaling cascade | AT1G65180, AT4G14980, AT2G42060 |
| *csld3* | 4.50E-09 | 0006073 cellular glucan metabolic process | AT5G57530, AT1G65310, AT4G28850, AT5G57540, AT4G25820, AT1G10550 |
|  | 1.10E-07 | 0016798 hydrolase activity, acting on glycosyl bonds | AT5G57530, AT1G65310, AT4G28850, AT5G57540, AT4G25820, AT1G10550 |
|  | 1.10E-05 | 0012505 endomembrane system | AT4G02270, AT1G34510, AT1G10550, AT4G40090, AT5G61350, AT5G57540, AT4G18640, AT1G18250, AT1G22500, AT1G30870, AT3G54040, AT3G54580, AT1G12560, AT4G13390, AT1G62980, AT4G19680, AT4G29180, AT5G10520, AT5G22410, AT4G28850, AT5G19800, AT3G49960, AT5G67400, AT4G31250, AT4G25820, AT3G15760, AT1G35330 |
|  | 8.20E-05 | 0004601 peroxidase activity | AT1G34510, AT1G30870, AT5G67400, AT3G49960, AT5G22410 |
|  | 2.80E-04 | 0006468 protein amino acid phosphorylation | AT5G61350, AT4G18640, AT3G46760, AT1G04700, AT1G71530, AT3G07070, AT5G61550, AT4G31250, AT4G29180, AT1G16440, AT5G11020 |
|  | 2.90E-04 | 0016301 kinase activity | AT5G61350, AT5G62310, AT3G46760, AT1G71530, AT3G07070, AT5G61550, AT4G31250, AT4G29180, AT5G10520, AT1G16440, AT5G11020 |
|  | 3.70E-04 | 0048765 root hair cell differentiation | AT2G03720, AT4G18640, AT3G54870 |
|  | 0.0025 | 0005576 extracellular region | AT4G25790, AT1G12560, AT1G62980, AT4G33730 |
|  | 0.0036 | 0006979 response to oxidative stress | AT1G34510, AT1G30870, AT5G67400, AT3G49960, AT5G22410 |
|  | 0.0039 | 0005975 carbohydrate metabolic process | AT5G57530, AT1G65310, AT4G28850, AT5G57540, AT4G25820, AT1G10550 |
|  | 0.0192 | 0005618 cell wall | AT5G57530, AT4G28850, AT5G57540, AT4G25820, AT1G10550 |
|  | 0.0257 | 0048768 root hair cell tip growth | AT3G54870, AT4G34580 |
|  | 0.034 | 0009826 unidimensional cell growth | AT5G62310, AT1G12560, AT1G62980 |
|  | 0.0377 | 0005215 transporter activity | [AT1G12950, AT3G54140, AT4G25220, AT4G34580](http://www.arabidopsis.org/servlets/TairObject?type=locus&name=AT1G12950) |
|  | 0.0397 | 0005089 Rho guanyl-nucleotide exchange factor activity | AT1G79860, AT2G45890, |
| *ire1* | 1.10E-07 | 0006073 cellular glucan metabolic process | AT5G57530, AT4G28850, AT5G57540, AT1G10550 |
|  | 3.40E-07 | 0005618 cell wall | AT5G57530, AT5G47550, AT4G28850, AT5G57540, AT3G54400, AT1G10550 |
|  | 7.30E-07 | 0016798 hydrolase activity, acting on glycosyl bonds | [AT5G57530, AT4G28850, AT5G57540, AT1G10550](http://www.arabidopsis.org/servlets/TairObject?type=locus&name=AT5G57530) |
|  | 3.40E-04 | 0048046 apoplast | AT3G54400, AT4G28850, AT5G57540, AT1G10550 |
|  | 5.20E-04 | 0005975 carbohydrate metabolic process | AT5G57530, AT4G28850, AT5G57540, AT1G10550 |
|  | 0.0356 | 0004601 peroxidase activity | AT1G34510, AT5G22410 |
| *lrx1* | 1.80E-07 | 0006073 cellular glucan metabolic process | AT5G57530, AT4G28850, AT5G57540, AT1G10550 |
|  | 1.20E-06 | 0016798 hydrolase activity, acting on glycosyl bonds | AT5G57530, AT4G28850, AT5G57540, AT1G10550 |
|  | 7.80E-04 | 0005618 cell wall | AT5G57530, AT4G28850, AT5G57540, AT1G10550 |
|  | 8.10E-04 | 0005975 carbohydrate metabolic process | AT5G57530, AT4G28850, AT5G57540, AT1G10550 |
|  | 0.0114 | 0048046 apoplast | AT4G28850, AT5G57540, AT1G10550 |
|  | 0.0409 | 0004601 peroxidase activity | AT1G34510, AT5G22410 |
|  | 0.048 | 0005576 extracellular region | AT5G66590, AT4G25790 |
| *mrh1* | 0.0015 | 0006073 cellular glucan metabolic process | AT4G28850, AT1G10550 |
|  | 0.0028 | 0016798 hydrolase activity, acting on glycosyl bonds | AT4G28850, AT1G10550 |
|  | 0.0218 | 0048046 apoplast | AT4G28850, AT1G10550 |
|  | 0.0247 | 0005618 cell wall | AT4G28850, AT1G10550 |
|  | 0.0251 | 0005975 carbohydrate metabolic process | AT4G28850, AT1G10550 |
|  | 0.2466 | 0012505 endomembrane system | AT4G28850, AT1G10550 |
| *mrh3* | 3.10E-06 | 0006073 cellular glucan metabolic process | AT5G57530, AT4G28850, AT5G57540, AT1G10550 |
|  | 1.90E-05 | 0016798 hydrolase activity, acting on glycosyl bonds | AT5G57530, AT4G28850, AT5G57540, AT1G10550 |
|  | 9.10E-05 | 0005618 cell wall | AT5G57530, AT5G47550, AT4G28850, AT5G57540, AT3G54400, AT1G10550 |
|  | 0.0048 | 0004601 peroxidase activity | AT1G34510, AT3G49960, AT5G22410 |
|  | 0.0066 | 0005576 extracellular region | AT4G25790, AT1G62980, AT4G33730 |
|  | 0.0077 | 0048046 apoplast | AT3G54400, AT4G28850, AT5G57540, AT1G10550 |
|  | 0.0113 | 0005975 carbohydrate metabolic process | AT5G57530, AT4G28850, AT5G57540, AT1G10550 |
|  | 0.0189 | 0012505 endomembrane system | AT4G28850, AT4G13390, AT3G49960, AT4G02270, AT1G62980, AT1G34510, AT3G54040, AT1G10550, AT5G57540, AT5G61350, AT5G22410 |
|  | 0.0325 | 0006979 response to oxidative stress | AT1G34510, AT3G49960, AT5G22410 |
|  | 0.0379 | 0031225 anchored to membrane | AT2G20520, AT4G22640, AT5G40960 |
| *myc1* | 0.0114 | 0048046 apoplast | AT3G54400, AT4G28850, AT1G10550 |
|  | 0.0116 | 0006073 cellular glucan metabolic process | AT4G28850, AT1G10550 |
|  | 0.0145 | 0005618 cell wall | AT3G54400, AT4G28850, AT1G10550 |
|  | 0.0209 | 0016798 hydrolase activity, acting on glycosyl bonds | AT4G28850, AT1G10550 |
| *rhd2* | 4.60E-07 | 0006073 cellular glucan metabolic process | AT5G57530, AT4G28850, AT5G57540, AT1G10550 |
|  | 2.90E-06 | 0016798 hydrolase activity, acting on glycosyl bonds | AT5G57530, AT4G28850, AT5G57540, AT1G10550 |
|  | 9.70E-05 | 0005618 cell wall | AT5G57530, AT5G47550, AT4G28850, AT5G57540, AT1G10550 |
|  | 0.0019 | 0005975 carbohydrate metabolic process | AT5G57530, AT4G28850, AT5G57540, AT1G10550 |
|  | 0.0199 | 0048046 apoplast | AT4G28850, AT5G57540, AT1G10550 |
|  | 0.0395 | 0012505 endomembrane system | AT4G28850, AT4G13390, AT1G34510, AT1G10550, AT4G40090, AT5G22410, AT5G57540 |
|  | 0.0467 | 0005737 cytoplasm | AT5G57530, AT1G69930, AT3G16800 |

*Mutants not shown did not yield gene sets with significant overrepresentation of any GO classes.
