# Supplementary material for: A Gene Regulatory Network for Root Epidermis Cell Differentiation in Arabidopsis
Source: PLoS Genet. 2012 Jan 12;8(1):e1002446. doi: 10.1371/journal.pgen.1002446 (PMC3257299; doi:10.1371/journal.pgen.1002446)
Supplement: Table S7 — Mean root hair length in root epidermis mutants and wild type. (DOCX) [file pgen.1002446.s015.docx]

**Table S7.** Mean Root Hair Length in Root Epidermis Mutants and Wild Type.

| **Genotype** | **Mean Root Hair Length (mm)** | **Standard Deviation * (mm)** |  |
| --- | --- | --- | --- |
| WT (Columbia) | 0.37 | 0.19 |  |
| *cobl9* | 0.25 | 0.12 |  |
| *cow1* | n.d. | n.d. |  |
| *cpctry* | n.d. | n.d. |  |
| *csld3* | 0.08 | 0.04 |  |
| *gl2* | 0.40 | 0.19 |  |
| *gl3 egl3* | 0.44 | 0.21 |  |
| *ire* | 0.20 | 0.11 |  |
| *lrx* | 0.28 | 0.13 |  |
| *mrh1* | 0.25 | 0.12 |  |
| *mrh2* | 0.21 | 0.12 |  |
| *mrh3* | 0.17 | 0.11 |  |
| *rhd2* | 0.07 | 0.04 |  |
| *rhd6* | n.d. | n.d. |  |
| *ttg1* | 0.61 | 0.19 |  |
| *wer myb23* | 0.28 | 0.17 |  |

* n.d. = not determined, due to difficulty in measuring the infrequent or unusually shaped hairs.
