# Supplementary material for: A Gene Regulatory Network for Root Epidermis Cell Differentiation in Arabidopsis
Source: PLoS Genet. 2012 Jan 12;8(1):e1002446. doi: 10.1371/journal.pgen.1002446 (PMC3257299; doi:10.1371/journal.pgen.1002446)
Supplement: Table S9 — Gene Ontology (GO) terms overrepresented among genes in the gene clusters in Figure 8. (DOCX) [file pgen.1002446.s017.docx]

**Table S9.** Gene Ontology (GO) Terms Overrepresented Among Genes in the Gene Clusters in Figure 8.

| **Gene Cluster** | **p-value** | **Gene Ontology (GO) Class** | **Genes** |
| --- | --- | --- | --- |
| Cluster A | 0.01182 | 0009505 plant-type cell wall | AT1G26770, AT3G54400, AT4G32460 |
| Cluster B | 0.0036 | 0009505 plant-type cell wall | AT1G65310, AT3G49220, AT3G61820 |
|  | 0.0077 | 0006073 cellular glucan metabolic process | AT1G65310, AT1G10550 |
|  | 0.014 | 0016798 hydrolase activity, acting on glycosyl bonds | AT1G65310, AT1G10550 |
| Cluster F | 0.0932 | 0016787 hydrolase activity | AT2G37440, AT1G15040 |
| Cluster H | 7.7e-07 | 0048765 root hair cell differentiation | AT5G49270, AT2G03720, AT5G65090, AT3G54870 |
|  | 4.8e-05 | 0006468 protein amino acid phosphorylation | AT2G41970, AT4G27290, AT5G61350, AT3G46760, AT1G04700, AT3G07070, AT4G25160, AT1G35670, AT5G61550, AT1G16440 |
|  | 3.1e-04 | 0016301 kinase activity | AT2G41970, AT5G61350, AT3G46760, AT5G62310, AT3G07070, AT4G25160, AT1G35670, AT5G61550, AT1G16440 |
|  | 0.0039 | 0005875 microtubule associated complex | AT1G73860, AT3G54870, AT1G09170 |
|  | 0.007 | 0003777 microtubule motor activity | AT1G73860, AT3G54870, AT1G09170 |
|  | 0.0263 | 0005089 Rho guanyl-nucleotide exchange factor activity | AT1G79860, AT2G45890 |
|  | 0.0282 | 0009932 cell tip growth | AT5G49270, AT5G65090 |
|  | 0.0429 | 0010053 root epidermal cell differentiation | AT5G49270, AT5G65090 |
|  | 0.0465 | 0006904 vesicle docking during exocytosis | AT5G13150, AT5G13990 |
| Cluster I | 6.3e-12 | 0005199 structural constituent of cell wall | AT5G06640, AT3G54580, AT4G13390, AT1G12040, AT3G62680, AT1G54970, AT5G35190 |
|  | 4.2e-06 | 0009664 plant-type cell wall organization | AT5G06640, AT3G54580, AT4G13390, AT5G35190 |
|  | 1.1e-04 | 0012505 endomembrane system | AT3G54580, AT4G13390, AT4G18640, AT5G67400, AT4G02270, AT1G18940, AT1G62980, AT4G25820, AT4G19680, AT3G54040, AT5G35190, AT4G29180, AT4G40090, AT1G35330 |
|  | 0.0045 | 0010054 trichoblast differentiation | AT1G12040, AT3G62680 |
|  | 0.0049 | 0005576 extracellular region | AT4G25790, AT1G62980, AT4G33730 |
|  | 0.0351 | 0009505 plant-type cell wall | AT1G12040, AT5G04960, AT3G10710 |
| Cluster J | 0.0128 | 0007242 intracellular signaling cascade | AT1G65180, AT4G14980 |
| Cluster N | 5.7e-06 | 0006073 cellular glucan metabolic process | AT4G28850, AT5G57530, AT5G57540 |
|  | 1.9e-05 | 0016798 hydrolase activity, acting on glycosyl bonds | AT4G28850, AT5G57530, AT5G57540 |
|  | 0.0015 | 0005618 cell wall | AT4G28850, AT5G57530, AT5G57540 |
|  | 0.0016 | 0005975 carbohydrate metabolic process | AT4G28850, AT5G57530, AT5G57540 |
|  | 0.0138 | 0004601 peroxidase activity | AT1G34510, AT5G22410, AT5G22410 |
|  | 0.0187 | 0012505 endomembrane system | AT1G34510, AT4G28850, AT5G22410, AT5G57540 |
|  | 0.0376 | 0006979 response to oxidative stress | AT1G34510, AT5G22410 |
